# Global network analysis in *Schizosaccharomyces pombe* reveals three distinct consequences of the common 1-kb deletion causing juvenile CLN3 disease

**DOI:** 10.1038/s41598-021-85471-4

**Published:** 2021-03-18

**Authors:** Christopher J. Minnis, StJohn Townsend, Julia Petschnigg, Elisa Tinelli, Jürg Bähler, Claire Russell, Sara E. Mole

**Affiliations:** 1 MRC Laboratory for Molecular Cell Biology and Great Ormond Street, Institute of Child Health, University College London, London, WC1E 6BT UK; 2 Department of Comparative Biomedical Sciences, Royal Veterinary College, Royal College Street, London, NW1 0TU UK; 3 Institute of Healthy Ageing, Department of Genetics, Evolution and Environment, University College London, London, WC1E 6BT UK; 4 The Molecular Biology of Metabolism Laboratory, The Francis Crick Institute, London, NW1 1AT UK

**Keywords:** High-throughput screening, Fungal genetics, Functional genomics, Genetic interaction, Eye diseases, Metabolic disorders, Neurological disorders, Disease genetics, Fungal genomics, Lipid-storage diseases, Neurodegeneration

## Abstract

Juvenile CLN3 disease is a recessively inherited paediatric neurodegenerative disorder, with most patients homozygous for a 1-kb intragenic deletion in *CLN3*. The *btn1* gene is the *Schizosaccharomyces pombe* orthologue of *CLN3*. Here, we have extended the use of synthetic genetic array (SGA) analyses to delineate functional signatures for two different disease-causing mutations in addition to complete deletion of *btn1*. We show that genetic-interaction signatures can differ for mutations in the same gene, which helps to dissect their distinct functional effects. The mutation equivalent to the minor transcript arising from the 1-kb deletion (*btn1*^*102–208del*^) shows a distinct interaction pattern. Taken together, our results imply that the minor 1-kb deletion transcript has three consequences for CLN3: to both lose and retain some inherent functions and to acquire abnormal characteristics. This has particular implications for the therapeutic development of juvenile CLN3 disease. In addition, this proof of concept could be applied to conserved genes for other mendelian disorders or any gene of interest, aiding in the dissection of their functional domains, unpacking the global consequences of disease pathogenesis, and clarifying genotype–phenotype correlations. In doing so, this detail will enhance the goals of personalised medicine to improve treatment outcomes and reduce adverse events.

## Introduction

Yeast provide useful model systems for many human diseases due their genetic tractability. We have developed yeast models to understand the neuronal ceroid lipofuscinoses (NCL), also known as Batten disease, a group of rare childhood inherited neurodegenerative disorders^1^. Most are inherited in an autosomal recessive manner, with mutations in 13 known human genes^2^, some of which are conserved in yeast. These diseases share the characteristic hallmarks of accumulation of autofluorescent material, lipofuscin/ceroid, in the lysosome^3^. The most prevalent NCL is juvenile CLN3 disease, with most patients carrying an intragenic 1-kb deletion of the gene *CLN3* on at least one of the two disease alleles^4,5^.

In this study, we extended application of the powerful synthetic genetic array technique using *Schizosaccharomyces pombe* to perform three independent systematic screens for three mutant strains of the orthologue of *CLN3, btn1*. These strains were (1) *btn1* encoding a mutation mimicking one transcriptional effect of the 1-kb deletion (*btn1*^*102–208del*^)^6,7^, (2) *btn1* encoding the equivalent of disease-associated missense mutation p.Asp416Gly (*btn1*^*D363G*^)^6^, (3), and complete deletion of *btn1* (*btn1*Δ)^8^.

Our objectives were to explore the genetic interactions of two disease-causing mutations, compare them to complete loss of the gene, identify proteins and key pathways that are involved in Btn1 function, and thereby indicate novel human orthologues that may contribute to CLN3 disease pathology and provide novel therapeutic targets. By dissecting functional regions, we have shown differences in the genetic interaction signatures for three distinct mutations in the same gene. The mutant equivalent to the most common mutation, the ‘1-kb deletion’, does not cause complete loss of function, but produces a complex partially functional Btn1 protein with a unique gain of new characteristics. This approach could be extended to understand functional consequences of disease-associated alleles in other genes with yeast orthologues, in addition to more generally dissecting the functional domains in any protein of interest.

## Results

### SGA analysis reveals overlapping genetic interactions for strains *btn1*Δ and *btn1*^*D363G*^, and many novel genetic interactions for *btn1*^*102–208del*^

#### Genetic interactions of three *btn1* mutant strains

**Figure 1:**
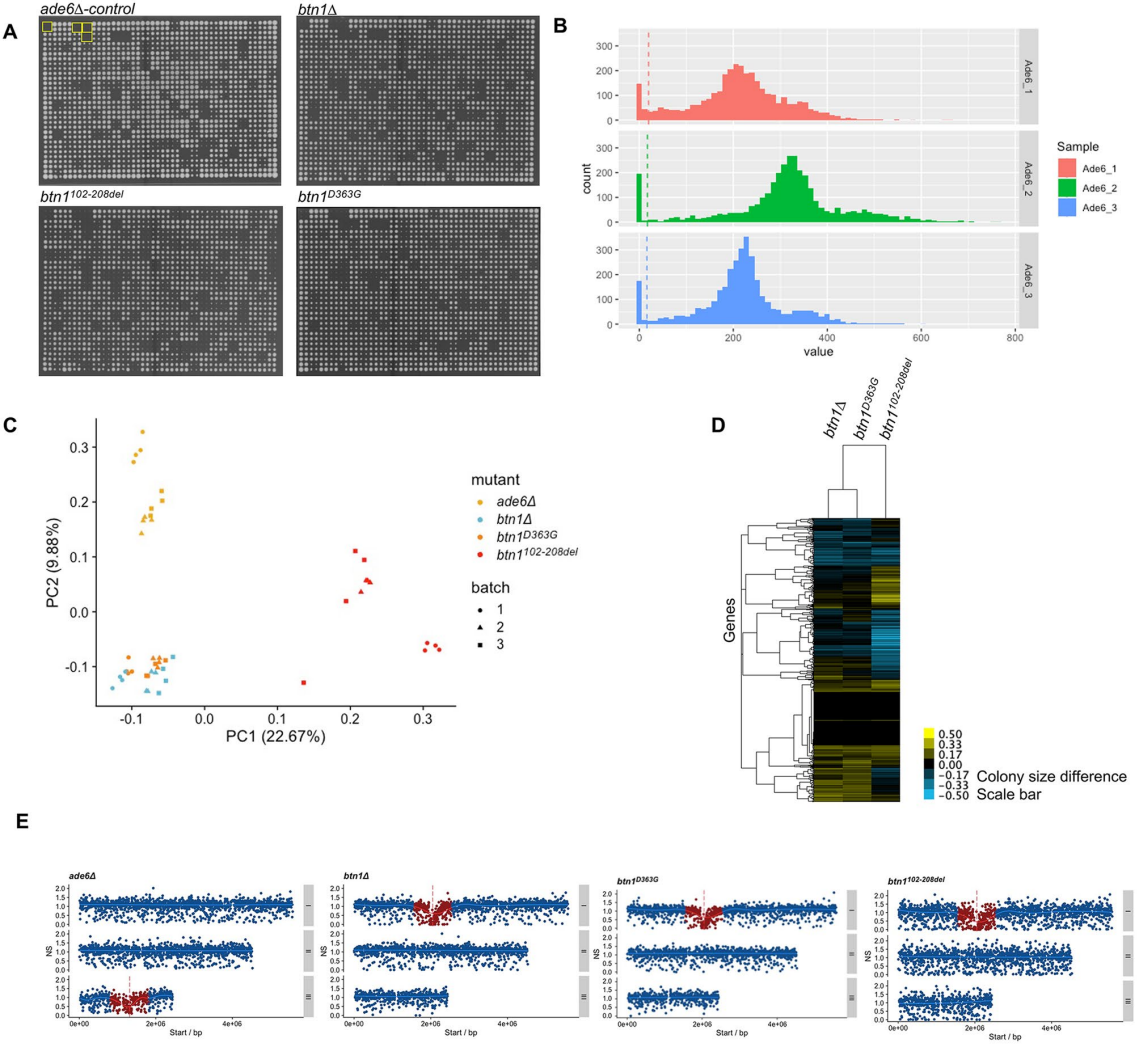
General overview of SGA analysis of *btn1* mutants versus *ade6* control. (**A**) Representative images of the SGA plates for control (*ade6*) and query mutants (*btn1∆, btn1*^*D363G*^*, btn1*^*102–208del*^), with empty control quadrants shown for *ade6* (yellow boxes). (**B**) Exclusion of small colonies for *ade6* control across batches as they represent high variability therefore reducing noise. (**C**) Principle component biplot of the variance within the SGA data for *ade6* control (yellow*)* and query-mutants *btn1*∆ (blue), *btn1*^*D363G*^ (orange), *btn1*^*102–208del*^ (red), with experimental batch indicated. (**D**) Cluster analysis for each strain and all the genes with their normalised colony size difference against *ade6* control with batch effects removed. Interactions are coloured in blue for negative interactions (< − 0.5) and yellow for positive interactions (> 0.5). (**E**) Gene linkage of normalised fitness score for *ade6* control and query mutants *btn1∆*, *btn1*^*D363G*^, *btn1*^*102–208del*^ from one experiment. Vertical dashed line represents *ade6* or *btn1* gene location, red points represent interaction scores excluded from data since less than 500 kb/500,000 bps from query gene location.

SGA screening was performed for the *ade6* control and three *btn1* mutants (*btn1∆*, *btn1*^*D363G*^ and *btn1*^*102–208del*^). Each Bioneer library mutant was crossed with each query strain independently 3 times and pinned in quadruplicate, providing a total of 12 replicate colonies for each double mutant (Fig. 1A). The *ade6∆* control showed similar growth distributions across all three independent experiments (Fig. 1B). Principal component analysis revealed good separation of query strains representative of their biological-signatures following normalisation of plate and batch effects (Fig. 1C). Cluster analysis of the *btn1* strains show similarities between *btn1*∆ and *btn1*^D363G^ and greater separation for *btn1*^*102–208del*^ for double mutant colony sizes after normalising for batch effects (Fig. 1D). Distribution of normalised colony sizes show decreased colony fitness close to the loci of query strains due to genetic linkage, genes within ± 500 kb range of the loci were excluded from our data (Fig. 1E).

**Figure 2:**
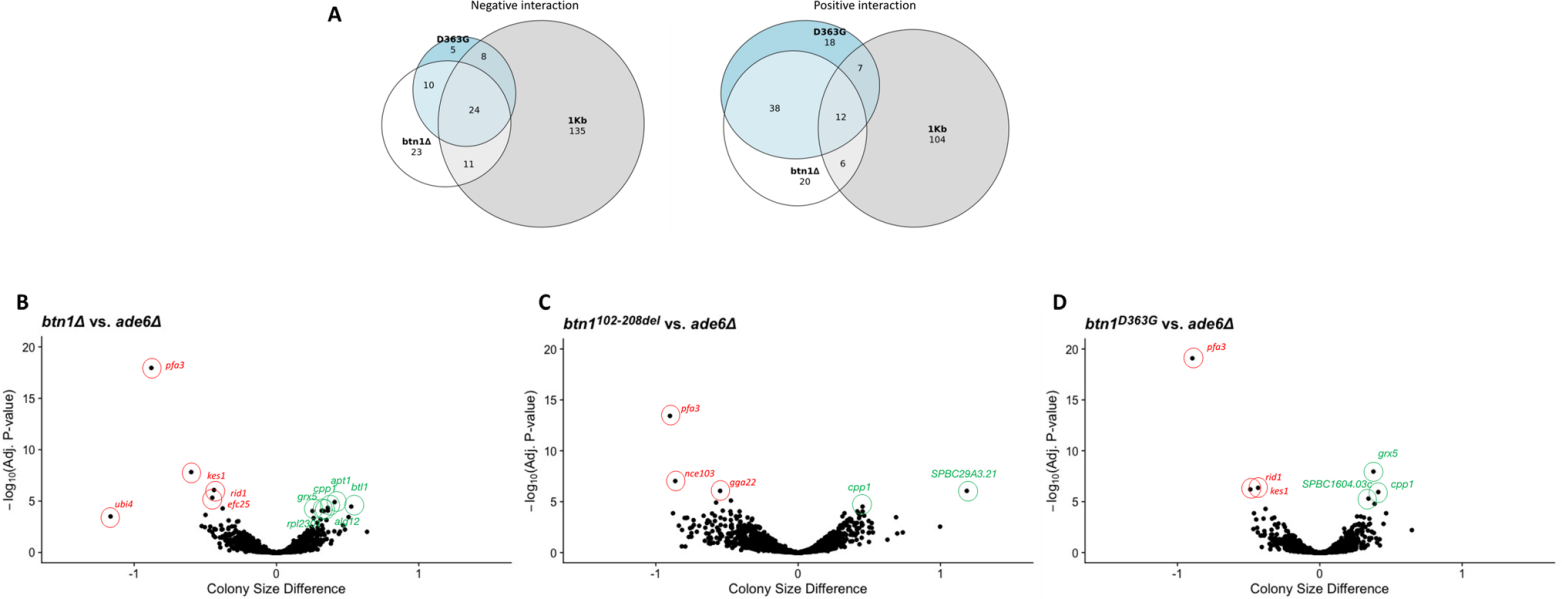
Venn diagrams and comparative volcano plots for strains *btn1∆, btn1*^*102–208del*^ (1-kb) and *btn1*^*D363G*^ (D363G) against control *ade6*. (**A**) Numbers represent robust hits across three independent SGAs for each query mutant. These include the subsets of genes that are shared between two or all mutants as well as their unique interactions. (**B**–**D**) Colony size difference plotted for each gene and each strain against the *ade6* control respectively (**B**) *btn1∆*, (**C**) *btn1*^*102–208del*^ (**D**) *btn1*^*D363G*^. Every gene had quad intra-repeats and the experiment was done in triplicate. All genes are plotted on the adjusted p-value on a log scale. The strongest interacting genes are highlighted as negative (red) or positive (green) interactors.

Interactions > 0.05 adjusted p-value across triplicate experiments were considered significant and placed into subsets of positive and negative interactions; the number of interactions is summarised in Table 1 and Fig. 2A (complete lists in supplementary tables S2, S3 and S4). *btn1*Δ has 76 positive interactions and 68 negative interactions, *btn1*^*102–208del*^ has 129 positive interactions and 178 negative interactions, and *btn1*^*D363G*^ has 75 positive interactions and 47 negative interactions. There are 84 interactions shared between *btn1∆* and *btn1*^*D363G*^ but only 36 consistent are robust hits across all three query strains (24 negative and 12 positive interactions). A summary of the differences between strains is presented in S5: supplementary tables.

**Table 1:** Summary of the number of significant gene interactions represented in the data for each mutant. Data is displayed as positive and negative interactions and shows numbers of hits common between all three *btn1* strains.

	*btn1*∆	*btn1*^*102–208del*^	*btn1*^*D363G*^	Shared between all strains
Positive	76	129	75	12
Negative	68	178	47	24
Total	144	307	122	36
Unique	43	239	23

As expected, there is considerable overlap between *btn1*∆ and *btn1*^*D363G*^ for their top interactions (Table 2). In contrast, the strongest interactors of *btn1*^*102–208del*^ overlap only two negative and one positive interaction with *btn1∆* and *btn1*^*D363G*^ (*pfa3, kes1* and *cpp1*, respectively)*.* The remaining strongest interactions for *btn1*^*102–208del*^ are unique.

**Table 2:** Top five negative and positive genetic interactions for *btn1* strains versus *ade6* control. Genes are listed in order of adjusted significant p-value (lowest first) with interactions shared by all three *btn1* strains shown in bold.

Strains	Interaction type	Top 5 genes
*btn1*∆	Negative	***pfa3****, ****kes1***, *rid1, ****efc25***, **ivn1**
	Positive	***apt1***, *blt1*, ***cpp1****, rpl2301, ***alg12**
*btn1*^*D363G*^	Negative	***pfa3****, rid1, ****kes1****, ****efc25, ****fhn1*
	Positive	*grx5, ****cpp1***,* SPBC1604.03c, ****apt1****, blt1*
*btn1*^*102–208del*^	Negative	***pfa3***, *nce103, gga22, clg1, ****kes1***
	Positive	*SPBC29A3.21, ****cpp1****, gfh1, tfx1, pub3*

#### Asp363 in the C terminus of *Btn1* is a critical residue for function

**Figure 3:**
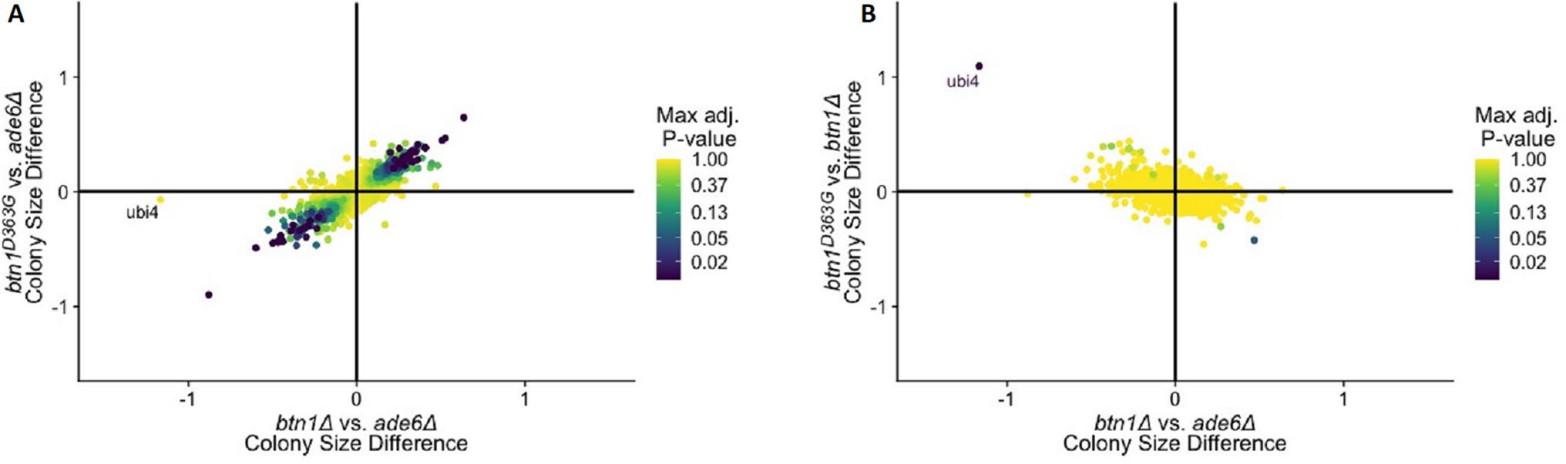
Comparative 2D-volcano plots for SGA queries of strains *btn1∆* and *btn1*^*D363G*^. (**A**) Biplot of colony size difference between *btn1∆* vs *ade6∆* on the x-axis and *btn1*^*D363G*^ vs *ade6∆* on the y-axis for each gene. Clustering of genes along the diagonal line highlights the similarities between the strains. Upper right quadrant represents shared positive interactions, lower left quadrant represents shared negative interactions. (**B**) Biplot of colony size difference between *btn1∆* vs *ade6∆* on the x-axis and *btn1*^*D363G*^ vs *btn1∆* on the y-axis. Clustering of most genes around the centre is due to the similarities between the two strains. Upper left and lower right quadrants represent residual functionalities. Genes away from the centre/at the extremes represent biological differences between the two strains. Gene points are represented by their max adjusted p-value score, with a logarithmic colour distribution.

The most significant interacting genes for *btn1*^*D363G*^ considerably overlap with those of *btn1∆*, with few interactions unique to each strain (Tables 1 and 2, Fig. 2). This is visualised by the PCA biplot (Figs. 1C, 3A). The shared interactions include 34 negative and 50 positive interactions. This suggests that the C-terminal missense mutation p.Asp363Gly leads to a near non-functional protein similar to complete lack of Btn1, indicating that Asp363 is a key functional residue. The equivalent human mutation, p.Asp416Gly may have the same drastic loss of function effect on CLN3^9^. Although other small differences cannot be ruled out, the main difference between the strains *btn1∆* and *btn1*^*D363G*^ may be the lack of a transcribed protein in *btn1∆* in contrast to production of a non-functional protein in *btn1*^*D363G*^. Indeed, there is a relative increase in transcriptional levels for *btn1* in both *btn1*^*D363G*^ and *btn1*^*102–208del*^ compared to WT (S1: supplementary figure 4C)*.*

#### Novel functionality is associated with the 1-kb deletion mutant protein

In contrast, the *btn1*^*102–208del*^ strain gives an overall global genetic interaction signature that is markedly distinct from the strains *btn1*Δ and *btn1*^*D363G*^*,* visualised in the separation between this strain and *btn1*Δ or *btn1*^*D363G*^ in the PCA biplot (Fig. 1C). There are many more interacting genes identified (more than double that for *btn1*Δ or *btn1*^*D363G*^), and most of these are unique for this strain (Table 1). These interacting genes are therefore not a simple subset of those highlighted when the function of *btn1* is lost but primarily comprise a large and novel set of genes, with only 17% of all *btn1*^*102–208del*^ hits overlapping with *btn1∆* (split 66% for negative and 33% for positive interactions). In contrast, 68% of *btn1*^*D363G*^ interactions overlap with *btn1*Δ. The direction of movement away from *btn1∆* and towards *ade6∆* in the PCA plot, suggests strongly that *btn1*^*102–208del*^ generates a mutant Btn1 protein that may retain some functionality associated with Btn1, while the tandem moving away from both *ade6∆* and *btn1∆* strains, and little overlap in genetic interactors with either of these strains, suggests the gain of new functionality (Fig. 1C).

### SGA analysis highlights multiple biological processes associated with *Btn1* function

#### *Btn1* function supports protein translation and trafficking

Considering all positive interactions for *btn1∆* and *btn1*^*D363G*^, there is an enrichment of GO terms for ribosomes (Table 3), suggesting that a reduction of translation is beneficial when Btn1 function is lost. However, only one gene encoding a ribosomal protein, *rpl230*1, is identified in the top five strong interactors. The remaining interactions for *btn1∆* are drawn from other cellular physiology pathways, particularly trafficking through the Golgi apparatus and endosomal compartments (*apt1, blt1, cpp1, grx5, cfr1* and *alg12*). There is no particular enrichment in GO terms for negative interactions of *btn1∆* and *btn1*^*D363G*^*.* In contrast, *btn1*^*102–208del*^ negative interactions are enriched for mitophagy in yeast while positive interactions are enriched for autophagy and pyruvate metabolism (all terms associated with strains are listed in the S6: supplementary tables).

**Table 3:** Enriched KEGG pathways for *btn1* strains versus *ade6* control^52^.

Strains	Interaction type	Enriched KEGG pathways	KEGG ID	No. of genes
*btn1*∆	Negative	*–*	*–*	*–*
	Positive	*Ribosome*	*KEGG:03010*	15
*btn1*^*D363G*^	Negative	*–*	*–*	–
	positive	*Ribosome, various types of N-glycan biosynthesis*	*KEGG:03010, KEGG:00,513*	16, 3
*btn1*^*102–208del*^	Negative	*Mitophagy—yeast*	*KEGG:04139*	5
	Positive	*Autophagy—other, pyruvate metabolism*	*KEGG:04136, KEGG:00620*	4, 4

G-profiler: Benjamini-Hotchberg FDR < threshold 0.1).

*KEGG* Kyoto Encyclopaedia of Genes and Genomes.

#### Palmitoylation becomes an essential function in the absence of full *Btn1* functionality

The strongest negative interactor of *btn1∆*, *pfa3,* is shared by all three strains (Fig. 2B–D), indicating that loss of function of this gene is detrimental both in the absence of Btn1 function or the presence of mutant Btn1^102–208del^. *pfa3* encodes a palmitoyltransferase that catalyses post-translational attachment of fatty acid palmitate to proteins via a cysteine residue. There can be diverse and severe consequences from dysfunctional palmitoylation and depalmitoylation, and insufficiency of the palmitoyl thioesterase Ppt1, defective in CLN1 disease^10^, has been observed in cells from CLN3 disease patients and a CLN3 mouse model^11,12^.

#### *Btn1* connects with the ubiquitin protease system

Although *btn1∆* and *btn1*^*D363G*^ strains have a similar genetic interaction pattern (Fig. 3), *ubi4* is a strong (synthetically lethal) negative interaction for *btn1∆* but not for *btn1*^*D363G*^ (Fig. 3). The expression of the non-functional Btn1^D363G^ protein is able to rescue the negative interaction between *btn1*∆ and *ubi4*∆. *ubi4* encodes a polyubiquitin protein precursor required in the response to stress and whose absence affects many pathways including meiosis^13,14^.

### Unpacking the complexity of *btn1*^*102–208del*^ to shed light on juvenile CLN3 disease

**Figure 4:**
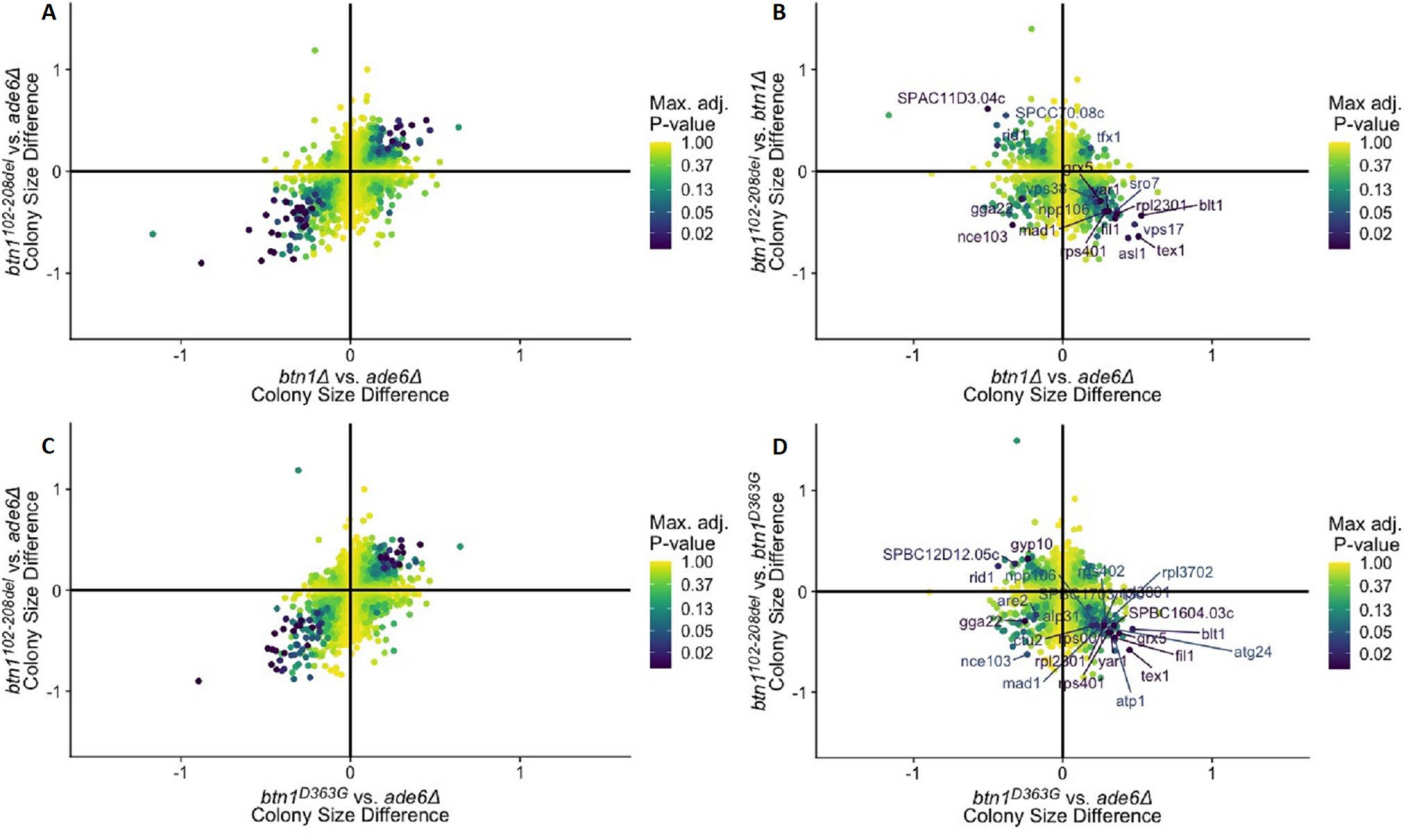
Comparative 2D-volcano plots for SGA queries of strains *btn1*^*102–208del*^ against *btn1∆* and *btn1*^*D363G*^. (**A**) Biplot of colony size difference between *btn1∆* vs *ade6∆* on the x-axis and *btn1*^*102–208del*^ vs *ade6∆* on the y-axis. Upper right quadrant represents shared positive interactions, lower left quadrant represents shared negative interactions. (**B**) Biplot of colony size difference between *btn1∆* vs *ade6∆* on the x-axis and *btn1*^*102–208del*^ vs *btn1∆* on the y-axis. Upper left and lower right quadrants represent residual functionalities in *btn1*^*102–208del*^ relative to *btn1∆*. Upper right and lower left quadrants represent gain of functionalities in *btn1*^*102–208del*^ relative to *btn1∆.* (**C**) Biplot of colony size difference between *btn1*^*D363G*^ vs *ade6∆* on the x-axis and *btn1*^*102–208del*^ vs *ade6∆* on the y-axis. Upper right quadrant represents shared positive interactions, lower left quadrant represents shared negative interactions. (**D**) Biplot of colony size difference between *btn1*^*D363G*^ vs *ade6∆* on the x-axis and *btn1*^*102–208del*^ vs *btn1*^*D363G*^ on the y-axis. Upper left and lower right quadrants represent residual functionalities in *btn1*^*102–208del*^ relative to *btn1*^*D363G*^. Upper right and lower left quadrants represent gain of function relative to *btn1*^*D363G*^. Gene points are represented by their max adjusted p-value score and a logarithmic colour distribution with significant genes annotated. For (**A**) and (**C**) genes away from the diagonal represent biological differences between the two strains. For (**B**) and (**D**) genes away from the centre/at the extremes represent biological differences between the two strains.

Comparison of *btn1*^*102–208del*^ to the *ade6*Δ control alone reveals only a portion of the likely complexity of the 1-kb deletion on CLN3 function. By additionally comparing *btn1*^*102–208del*^ to *btn1*Δ (Fig. 4), we are able to dissect the interactions into specific subsets; this new information reveals the consequence of this mutation and is relevant to understanding the impact of the 1-kb deletion in CLN3 disease. These include shared positive and negative interactions and gain of function of *btn1*^*102–208del*^ relative to strains *btn1∆* or *btn1*^*D363G*^.

#### *Btn1*^*102–208del*^ loses functions that are also missing in *btn1∆*

The consequences driven by the loss of amino acids 102–208 of Btn1 can be compared with those of *btn1∆*. The same effect on colony size indicates interactions that are shared between *btn1*^*102–208del*^ and *btn1∆* (Fig. 4A). Therefore, these genetic interactions must be associated with the loss of functionality caused by deletion of amino acids 102–208. Positive interactions shared by *btn1*^*102–208del*^ and *btn1∆* are enriched for N-glycan biosynthesis (*alg9*, *alg12*), and protein farnesylation (*cpp1*) (S7: supplementary tables for common lost hits for 1-kb). Shared negative interactions are enriched for sterol lipids and trafficking (*pfa3* and *kes1)*.

#### *Btn1*^*102–208del*^ loses functions that are also missing in expressed *Btn1*^*D363G*^

As above, the consequences driven by *btn1*^*102–208del*^ can be compared with those of *btn1*^*D363G*^ (Fig. 4C). Both strains express mutant Btn1 proteins with p.Asp363Gly nearly equivalent to complete absence of Btn1 function (see above). As expected, the same effect on colony size is shared by many of interactions of *btn1∆* and *btn1*^*102–208del*^ and the interactions of *btn1*^*D363G*^ and *btn1*^*102–208del*^. This indicates loss of the same functions for Btn^102–208del^ and Btn1^D363G^.

#### *Btn1*^*102–208del*^ exhibits functions that are missing in *btn1∆*

Through characterising differences between *btn1*^*102–208del*^ and *btn1∆,* we observe a broad set of genes that show synthetic sickness in *btn1∆*, but for which fitness is at least partially restored in the *btn1*^*102–208del*^ (Fig. 1D). The converse is true for a set of *btn1*Δ positive interaction (Fig. 1D). That some of the interactions of *btn1*Δ are reversed in *btn1*^*102–208del*^ indicates that there is a partially functional Btn1 protein in the *btn1*^*102–208del*^ mutant. To identify this set of genes we use comparative 2D volcano plots to separate residual restorative functionality and increased sensitivity in the *btn1*^*102–208del*^ strain (Fig. 4B). *btn1*^*102–208del*^ maintains residual Btn1 functionality, represented by opposing genetic interactions from the *btn1∆* perspective. These genes are clustered in the top left and bottom right quadrants of Fig. 4B and show the restorative positive and negative interactions by *btn1*^*102–208del*^ (full list of genes in S8: supplementary tables). We postulate that these observations are a consequence of normal functional domains remaining in the Btn1^102–208del^ protein, thus effectively reverting colony fitness back to that of the *ade6* control. Similar observations are seen when *btn1*^*102–208del*^ was compared with *btn1*^*D363G*^ (Fig. 4D).

The gene, *kgd1*, encoding 2-oxoglutarate dehydrogenase^15^, has the strongest negative statistical difference between *btn1*^*102–208del*^ and *btn1∆* (and one of the largest differences when comparing to *btn1*^*D363G*^). Indicative of the complexity of the 1-kb deletion it exemplifies a complete reversal of interactions between *btn1*^*102–208del*^ and *btn1∆* on protein function.

#### Expressed *Btn1*^*102–208del*^ gains novel functions that are not present in wildtype *Btn1*

**Figure 5:**
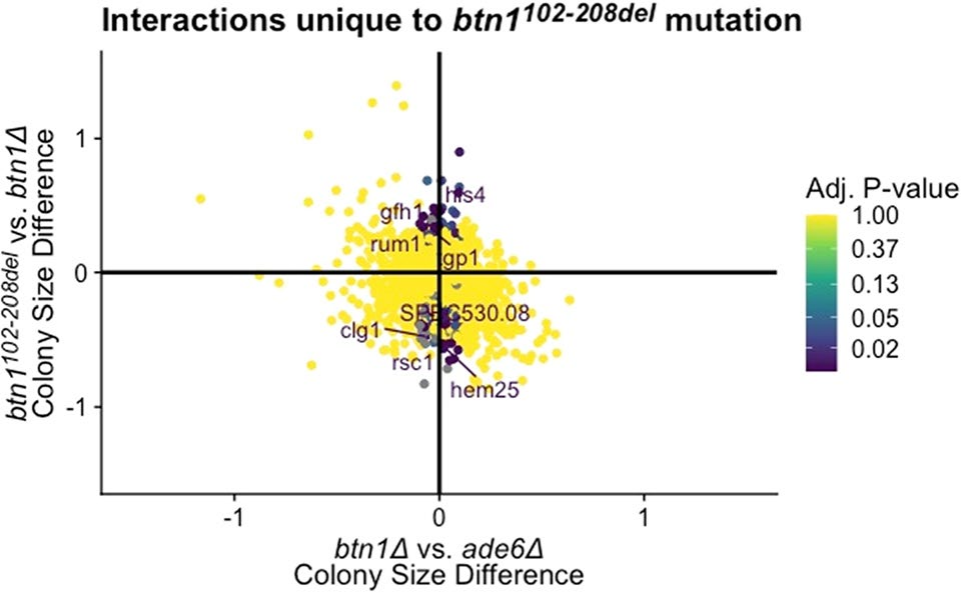
Gain of function interactions unique to *btn1*^*102–208del*^. This 2D volcano plot of colony size difference between *btn1∆* and *ade6∆* (x-axis) against colony size difference between *btn1∆* vs *btn1*^*102–208del*^ (y-axis) highlights the gain of function interactions unique to *btn1*^*102–208del*^. Points highlighted around the vertical axis represent genes that had no colony difference between *btn1∆* vs *ade6∆* control, however were significant for *btn1*^*102–208del*^*.* For simplicity any interaction that did not meet the criteria for unique gain of function was changed to 1 adjusted p-value (yellow). Gene points are represented by their adjusted p-value score for interaction *btn1*^*102–208del*^ vs *ade6∆,* colour represented by a logarithmic colour distribution with significant genes (adjusted p-value < 1e^−3^) annotated. Full list of genetic interactions in S10: supplementary tables.

There are two main approaches to determining gain of functions: (1) interactions that show increased severity in one mutant compared to the other, and (2) interactions which are not present in the ablated query but are present in the other mutant query. A marked feature of our dataset is the complex nature of the loss of amino acids 102–208 on Btn1 protein function, revealed in Fig. 4B, contrasting with the effect of the missense mutation p.Asp363Gly displayed in Fig. 3B. We confirm *btn1*^*102–208del*^ increases sensitivity for genetic interactions compared to those of *btn1∆* within the bottom left and top right quadrants. For example, our data sets reveal two negative interactions within this subset, (*gga22, nce103*) and one positive interaction (*tfx1*) S9: supplementary tables. These represent a potential gain of function by loss of amino acids 102–208. In addition, we identify interactions that are unique to *btn1*^*102–208del*^ by using the criteria representing a significant difference in colony size between *btn1*^*102–208del*^ and *btn1∆* but no difference in colony size for *btn1∆* vs *ade6∆* interactions (Fig. 5). We can conclude that the Btn1^102–208del^ mutant protein has a clear gain of function represented by both unique positive and negative interactions (summarised in Fig. 5).

#### *Btn1*^*102–208del*^ links to genes implicated in other diseases

Many of the interactions for the three *btn1* strains link to human disease (Table 4). For the unique negative interactions of *btn1*^*102–208del*^, where introduction of this mutation into cells deleted for another gene causes these cells to become synthetically sick, the interacting genes are linked to eye diseases (*gal1, aim22, fab1, ath1, rad24*), epilepsies (*tef103, rad24*), inherited inborn mitochondrial (*hem25, mss1*), neurological (*sst2, rav1*) immune system (*ski3*) and lipid metabolism (*erg32*) disorders. For the unique positive interactions, where introduction of *btn1*^*102–208del*^ into cells deleted for another gene causes cells to grow better, these genes are also linked to eye disorders (*gfh1, hnt3*) as well as lysosomal disorders (*npc2, SPBC713.07c, SPBC1683.12*), monogenic diseases (*hri2, kms1, cds1*), neurological (*hhp2, SPCC1827.07c*), and inborn mitochondrial metabolomic (*cyt1, fsf1, SPBC8D2.18c*) disorders. This suggests a shared compensatory network between these varied disorders and CLN3 disease (Table 5).

**Table 4:** Summary of human diseases and their corresponding yeast genes for the interactions of* btn1*^*102–208del*^ mutant*, btn1*^*D363G*^ and* btn1∆,* versus the *ade6* control (Full list of genes against each MONDO ID in S11: supplementary table). The unique interactions of btn1^102–208del^ are summarised in Table 5.

Disease terms	MONDO ID	No. of genes		
		Strains		
		*btn1*^*102–208del*^	*btn1*^*D363G*^	*btn1∆*
Kidney disease	MONDO:0005240	16	4	6
Eye disease	MONDO:0005328	15	6	6
Inborn mitochondrial metabolism disorder	MONDO:0004069	13	4	3
Epilepsy	MONDO:0005027	12	6	7
Autosomal recessive disease	MONDO:0006025	12	5	6
Cancer	MONDO:0004992	11	8	7
Skin disease	MONDO:0005093	11	5	3
Autosomal dominant disease	MONDO:0000426	10	1	3
Hematologic disease	MONDO:0005570	10	6	2
Carbohydrate metabolism disease	MONDO:0037792	3	0	2
Immune system disease	MONDO:0005046	8	4	2
Peripheral neuropathy	MONDO:0005244	6	0	2
Inherited lipid metabolism disorder	MONDO:0002525	7	3	3
Anaemia (disease)	MONDO:0002280	6	5	2
Bone development disease	MONDO:0005497	6	3	1
Digestive system disease	MONDO:0004335	6	3	3
Heart disease	MONDO:0005267	6	1	0
Neurodegenerative disease	MONDO:0005559	5	0	3
Cognitive disorder	MONDO:0002039	4	0	2
Lysosomal storage disease	MONDO:0002561	4	0	1
Myopathy	MONDO:0005336	4	0	0
Premature aging syndrome	MONDO:0019303	4	4	2
DNA repair disease	MONDO:0021190	3	0	0
Inborn disorder of purine or pyrimidine metabolism	MONDO:0019254	2	4	1
Inherited amino acid metabolic disorder	MONDO:0004736	3	2	2
Nonsyndromic genetic deafness	MONDO:0019497	3	0	2
Diabetes mellitus (disease)	MONDO:0005015	2	0	0
Hepatobiliary disease	MONDO:0002515	1	1	1
Dystonic disorder	MONDO:0003441	0	1	2
Peroxisomal disease	MONDO:0019053	0	1	0

Subnote: One gene may cause more than one disease.

**Table 5:** Summary of human diseases and their corresponding yeast genes for the unique interactions of btn1^102–208del^ mutant. (Full list of diseases and genes associated can be found in S12: supplementary table).

Strain	Disease	Genes
*btn1*^*102–208del*^	Autosomal disease	*ath1, SPBC1683.12, cds1, rav1, hnt3, hri2, kms1, hem25, npc2, rav1, SPBC713.07c*
	Epilepsy	*rad24, SPBC1683.12, tef103, SPBC713.07c*
	Eye disease	*fab1, hnt3, ath1, gal1, gfh1, aim22, SPBC713.07c*
	Inborn mitochondrial metabolism disorder	*cyt1, aim22, hem25, fsf1, mss1*
	Lysosomal storage disease	*SPBC1683.12, npc2, SPBC713.07c*
	Peripheral neuropathy	*ath1, aim22, rav1, SPBC713.07c*

Subnote: One gene may cause more than one disease.

#### *Btn1*^*102–208del*^ novel functions link with multiple pathways

**Figure 6:**
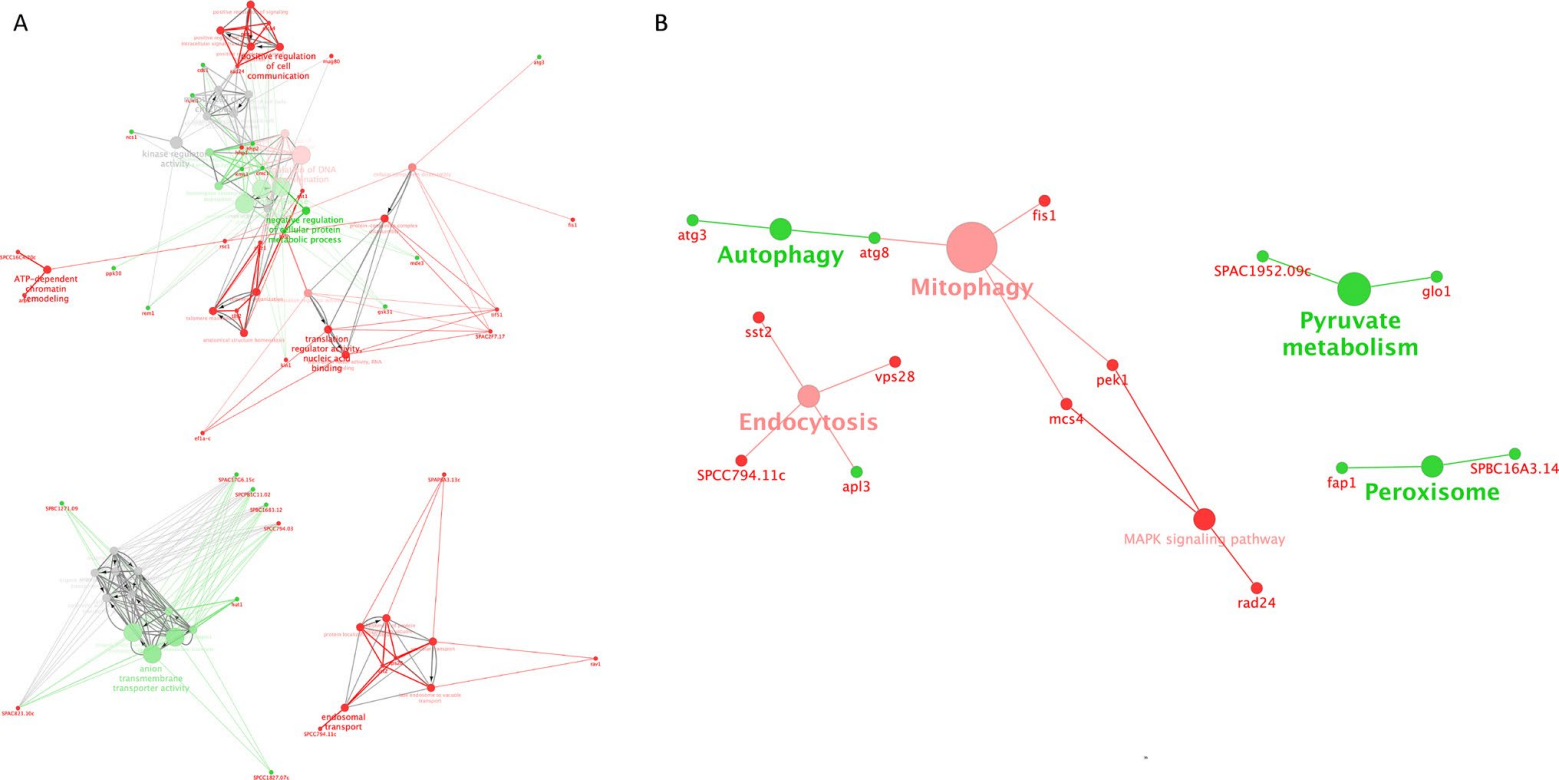
Genetic network of unique *btn1*^*102–208del*^ interactions. (**A**) Genetic networks of unique *btn1*^*102–208del*^ interactions represented in terms of positive (green) and negative (red) interacting gene and their corresponding biological processes GO term. Grey GO terms represent terms with both interactions, with a scale of red or green dependent on the number of interactions of either side associated with that specific GO term. (**B**) Genetic networks of unique *btn1*^*102–208del*^ interactions represented in terms of positive (green) and negative (red) interacting gene and their corresponding KEGG GO term. Kappa score = 0.4. Generate using ClueGO^51^.

We investigated the KEGG networks for the *btn1*^*102–208del*^ unique gain of function interactions, generating cytoscape networks with ClueGO integration (Fig. 6B). Negative interactions localise in endocytosis (*sst2, vps28, SPCC794.11c*), mitophagy (*fis1, mcs4, pek1*), and the MAPK signalling pathway (*rad24, mcs4, pek1*). Positive interactions involve autophagy (*atg3, atg8*), pyruvate metabolism (*glo1, SPAC1952.09c*) and peroxisome function (*fap1, SPBC16A3.14*). In addition, there is significant enrichment for GO cellular compartment terms that relate to integral components for peroxisomes for the negative interactions (*fis1, inp2*). Biological processes for positive interactions enrich for regulation of ion transmembrane transport (*ncs1, gti1*), divalent inorganic cation transport (*fet4, SPAC17A2.14*), and anion transporter activity (*SPCC1827.07c, SPBC1683.12, SPBC1271.09, hut1, SPAC17G6.15c, SPCPB1C11.02*); those for negative interactions include protein tyrosine kinase activity (*hhp1, hhp2 pek1*) and ATP dependent chromatin remodelling (*arp5, SPCC16C4.20c, rsc1*). Some GO terms have both positive and negative interactions: these relate to cell cycle checkpoint [positive (*cds1, rum1, hhp2*)/negative (*hhp1, rad24*)] and kinase activity (positive (*ncs1, rum*1)/negative (*tif51, mug80, mcs4*) (Fig. 6A).

## Discussion

Through a novel application of SGAs we compared genetic-interaction signatures and the global effects of two different disease-causing mutations (*btn1*^*102–208del*^ and *btn1*^*D363G*^) with complete loss of *btn1*. Our analysis reveals that genetic-interaction signatures can be specific for mutations in the same gene, which helps in the dissection of their distinct functional effects.

We had already demonstrated a role for Btn1 in TOR signalling and vacuole homeostasis, and clear involvement in multiple pathways in *S. pombe*^6,8,16,17^. This work confirms these previous findings and additionally reveals that Btn1 contributes to trafficking and could be important for ubiquitin–proteasome system (UPS) regulation. We also demonstrate that the residue Asp363 (in *H.s* CLN3 Asp416) is crucial, as a mutant protein containing p.Asp363Gly is equivalent to complete absence of *btn1*.

A significant finding is that the mutant protein Btn1^102–208del^, which is equivalent to a transcript of the most common pathogenic mutation in *CLN3*, the '1-kb deletion', has lost significant Btn1 functions but is not equivalent to complete absence of all Btn1 functions. Further, this mutant protein, which is missing amino acid residues 102–208 (equivalent to CLN3 residues 154–259), also acquires novel functions, as well as retaining some functions of Btn1.

These findings have important implications in terms of understanding the consequences of the 1-kb deletion on juvenile CLN3 disease pathogenic mechanisms and in designing therapeutic interventions. There is much variation in transcription in the brain^18^, the site of significant CLN3 disease pathology, and this can have functional consequences^19^. Variant CLN3 transcripts are known that lack exons, some of which affect phasing, and further novel isoforms arise in the presence of the 1-kb deletion^7^. Therefore, questions remain as to whether the observed disease pathogenesis is due to the specific partial loss of CLN3 function associated with a particular mutant transcript or includes the acquisition of new characteristics, as implied by this work, and whether the disease severity correlates with the prevalence of particular variant mutant transcripts. This is not known for the CLN3 human disease, but an increased level of *btn1* mutant transcripts were observed in both *btn1*^*D363G*^ and *btn1*^*102–208del*^ compared to wildtype *btn1*. If CLN3 disease is directly related to increased expression of one or more mutant variant transcripts, it may be that manipulation of their concentration could reduce or exacerbate the disease. Therapeutically, introduction of a full length CLN3 transcript may compensate only for the loss of function. Novel strategies would need to be developed for aspects of disease arising from acquired characteristics. It cannot yet be ruled out that high expression of the mutant transcripts is a compensatory mechanism due to less functional or non-functional transcripts. It is possible that having high levels of the partially-functional 1-kb protein is responsible for the gain of function. Introducing a full-length transcript might remove these gains of functions by repressing transcription of the native 1-kb transcript.

Through the enhanced use of the SGA with *S. pombe* we have extended the utilisation of this powerful technique beyond complete loss of function. The advantages of conducting in-depth analysis of multiple mutants within a single gene are clear with the delineation of novel distinctions between *btn1*^*D363G*^*, btn1*^*102–208del*^ and *btn1*∆. Here, comparative analyses of the interactions of all three query strains show a trifecta of consequences of the Btn1^102–208del^ protein that can be confidently divided into three distinct categories: partial loss of Btn1 functions, retention of functions and gain of novel functions. There is an advantage in using *S. pombe* to delineate genetic interactions as there is less functional redundancy in its genome compared to *S. cerevisiae*^20^, which will have contributed to cleaner phenotypic consequences and aided interpretation; such functional insights may otherwise have been obscured through the use of other comparative experimental approaches such as transcriptomics or proteomics. Further, this approach avoids the constraints of these methods where it is not always possible to correlate transcript isoforms and protein expression levels with phenotype.

In terms of understanding the fundamental function of Btn1, genes that are positive phenotypic suppressors for both *btn1*Δ and *btn1*^*D363G*^, but not *btn1*^*102–208del*^, can reflect physical interactions with Btn1. Of interest, we identified *sdo1* as a positive interaction. This encodes a protein involved in ribosome maturation and is the orthologue of *SBDS* which causes Shwachman–Bodian–Diamond syndrome^21^. It has previously been reported that *S. cerevisiae* Btn1p protein binds the protein Sdo1p and overexpression of *BTN1* could compensate for loss of *SDO1*^22^.

This work has highlighted that *btn1* is essential for the survival of several genetically deficient cells. One of these is *ubi4∆*, yet crossing with *btn1*^*D363G*^, a mutant strain with a very similar genetic-interaction signature to *btn1∆* (thereby inferring that Btn1^D363G^ protein is non-functional), was sufficient to rescue this strain. The polyubiquitin protein precursor *ubi4* is key to normal cellular physiological processes and to stress survival^14^. The multi-unit structure of Ubi4 enables the fast release of free ubiquitin and rapid response to acute stress such as oxidative stress, heat stress or toxicity that activates the unfolded protein response (UPR)^13,23,24^. It is therefore intriguing that expression of Btn1^D363G^ is able to ameliorate this synthetically sick interaction arising from complete loss of the *btn1* gene in *btn1∆*. Expression of Btn1^D363G^, even though essentially non-functional, could explain differences observed between the pleiotropic phenotypes of *btn1∆* and *btn1*^*D363G*^ strains (data unpublished). Further, this unsuspected role of Btn1, that we suggest is secondary to its main functional role, may be mirrored in its human orthologue.

One of the strongest interactors of all the *btn1* mutants was *pfa3,* which encodes a protein-cysteine S-palmitoyltransferase*.* Palmitoylation is essential for many biological processes such as neuronal development, particularly relevant to the NCLs^25,26^. Disruption of palmitoylation can lead to neurological defects^27^. Pfa3 has at least four homologues in humans, the zinc finger DHHC domain-containing palmitoyltransferases, ZDHHC2, ZDHHC20, ZDHHC15, and ZDHHC21, none of which are well characterised^27^. However, ZDHHC15 is highly expressed in many central nervous system cell types, with neurons exhibiting shorter dendrites when ZDHHC15 is depleted^28^. Intriguingly, ZDHHC2 was highlighted in a recent translatome-regulatory network analysis as a neuroprotective protein when overexpressed in line with our findings and is down-regulated in incipient Parkinson's disease^29^.

The full and complex consequences of the ‘1-kb deletion’-equivalent *btn1*^*102–208del*^ are revealed for the first time. This conceptual increment in genetic understanding allows inference of the likely consequences of the 1-kb deletion in juvenile CLN3 disease. No existing mouse or zebrafish CLN3 models mimic only the acquisition of novel dominant functionality by 1-kb transcripts; *btn1*^*102–208del*^ is the first to mimic the minor transcript seen in a patient’s fibroblasts^7^. Further, many unique genetic interactions of *btn1*^*102–208del*^ correspond to conserved human orthologues, including genes related to eye and neurological diseases, for example, *npc2* (Niemann–Pick C disease), linking these diseases with the pathogenesis of CLN3 disease. Our enrichment of unique negative interactions of *btn1*^*102–208del*^ suggests that the ‘1-kb deletion’ may be detrimental to cells sensitive to aberrant mitophagy. This is not surprising given the accumulation of subunit c of mitochondrial ATP synthase, a hallmark of CLN3 disease and other NCLs^30,31^.

Our results also pinpoint potential novel interactions specific to the minor 1 kb transcript. Positive interactions in this subset of genes may be useful in developing specific therapeutic targets that would ameliorate phenotypes solely arising from this mutated transcript. Disease caused by other mutations in CLN3 may not respond to these tailored therapeutics.

Notably, our analysis has identified several genes of interest for further study, such as *kgd1* and *glo1*. Many of these reveal a web of interlocking connections common across multiple neurological diseases^32,33^. When we compared our results with the orthologues of biomarkers for juvenile CLN3 disease collated from murine microarray data^34,35^, patients homozygous for the 1-kb deletion^36^ and SH-SY5Y Human Neuroblastoma cells^37^ (S13: supplementary table), five genes, *pfa3, kes1, myo52, efc25* and *cox17*, were found to be common.

## Conclusion

This proof of concept extension of the SGA described here has revealed novel and unexpectedly complex findings for *btn1* with implications for therapeutic development for CLN3 disease. The use of SGA in this manner, focusing on an individual gene with mutations selected for their functional or pathogenic consequences could reveal novel findings for many genes. This approach applied to conserved genes, particularly those related to rare mendelian diseases but also genes associated to more common diseases such as Parkinson’s and Alzheimer’s, will aid the dissection of their functional domains and consequences of disease pathogenesis. Moreover, it will enable further correlations with genotype and enhance the goals of personalised medicine to improve treatment outcomes and reduce adverse events.

## Methods

### Routine yeast methods

Yeast cells were grown in YES broth (Formedium) and incubated for a period of 24hrs at 30 °C. Strains were also grown on YES Agar (Formedium) plates and stored at 2–8 °C. Query strains generated and used: *btn1∆*, *btn1*^*102–208del*^ and *btn1*^*D363G*^.

### Generation of query strains

*Schizosaccharomyces pombe* query strains were generated as follows using primers in Table 6. For *btn1∆* (*h-, leu1-31, ura4-D18, ade6-M210, btn1::NatMX*), a wildtype strain (ALU-) was used as parental strain and the NatMX cassette was introduced through homologous recombination by transformation of a purified PCR product containing NatMX and flanking regions. Primers were designed (using pFa6a-NatMX plasmid as a PCR template) so that the endogenous *btn1* gene was swapped for the PCR-amplified NatMX cassette, containing 50 bp homologous regions flanking *btn1*. For *btn1*^*102–208del*^, a *btn1::btn1*^*102–208del*^ strain (ALU-) was used as parental strain and the NatMX cassette was introduced at the C-terminus via homologous recombination. For *btn1*^*D363G*^ query strain, a *btn1::btn1*^*D363G*^ parental strain (ALU-) was transformed with a PCR-product containing the NatMX-cassette and 50 bp flanking regions of the C-terminus of *btn1*. All strains were confirmed through agarose gel size comparison with wildtype btn1-strain. Genomic DNA was extracted from all query strains and the btn1-regions were PCR-amplified with primers upstream of *btn1* and in the NatMX cassette. PCR products were separated on an agarose gel and compared to wildtype-*btn1*. Additionally, strains were confirmed through sequencing: briefly, primers were designed upstream of the mutated btn1 genes and in the NatMX cassette and resulting PCR fragments were sequenced with various sequencing primers to cover the D363G mutation and the 102–208del regions. Transformations of the parental strains were performed using standard protocols using lithium acetate^38^, followed by selection of positive clones on YES plates containing ClonNat (100 μg/ml).

**Table 6:** Primers used to integrate and generate *btn1* mutants, *btn1*^*D363G*^ and *btn1*^*102–28del*^.

Primers		Sequences
Upstream btn1 control primers	5′UTR_F_ + 150	GAAGTCATCGATAGAAGGGCT
	sequ_R	GTCGTAAAGCAAACTTGATATTG
Downstream btn1 control primers	sequ_F	CTACTATCGTATTGCCGGTTC
	3′UTR_R_-240	TCGCATAGAATGCACAGCAG
Btn1^102–208del^ control primers	btn1_120-208_F	AAGCATTTTCGAAGGGACGG
	btn1_120-208_R	TTCAATTTCGTTAATTCTTTGAAGCAAAC
Btn1^D363G^ mutation primers	D363G_F	ttagtactgttggctcttcaGGTagctctggaatttttttagcttc
	D363G_R	gaagctaaaaaaattccagagctACCtgaagagccaacagtactaa
Ura4 integration primers	ura4-knockin_F	actttacaaaagtctactatcgtattgccggttcaaactaaaatttatgacctcagtttctactaaatatagtacgaatcATGGATGCTAGAGTATTTCAA
	ura4-knockin_R	tattgaagcacttagcacactaatggaagaaacgtcataggttaactctcaatataatggagatgtgcgcactcactattTTAATGCTGAGAAAGTCTTTG
Ura4 control primers	ura4_F	ATGGATGCTAGAGTATTTCAAAGC
	ura4_R	TTAATGCTGAGAAAGTCTTTGC

### Yeast RNA extraction

After overnight growth in 15 ml of YES media, cells were broken up using glass beads (0.5 mm diameter) with buffer made of RTL buffer + beta-mercaptoethanol (10 μl in 1 ml of RLT buffer). Following this the cells were vortexed in the cold room at 4 °C for 20 min. To elute the cells contents into a new microfuge tube, the side and bottom of the tube was pierced, followed by centrifugation for 5 s. Spin for 2 min for 10,000 rpm. Transfer 350 μl of lysate was transferred to the RNA column (Qiagen) and the Qiagen protocol (RNeasy mini kit) was followed to purify the RNA in 30 μl Rnase free water.

**Table 7:** Primers for yeast qPCR. Used to explore the expression levels of *btn1* in WT, *btn1*∆, *btn1*^*102–208del*^ and *btn1*^*D363G*^.

Primers qPCR		Sequences
btn1 pair 1	Forward	TGCGAAAGTAGGGTGTTGCT
	Reverse	GAAGAATTGATGCTGACAAC
btn1 pair 2	Forward	TTGTTCCTTCTTTGGCGTGC
	Reverse	AGCATTTTCGAAGGGACGGA
btn1 pair 3	Forward	ATGTTTCCGCAGTTCCTTGT
	Reverse	CGCGATAGGAAAACACCAAT
Actin pair 1	Forward	GTTATGTCTGGTGGTACCACT
	Reverse	GATCCACCAATCCAGACAGA

### RNA reverse transcription

Extracted RNA was retro-transcribed using the QuantiTect reverse transcription kit (Qiagen**).** Reaction tubes were incubated at 42 °C for 15 min and inactivated at 95 °C for 3 min. Samples were used immediately. Regular PCR was performed to verify primers worked efficiently.

### RT-qPCR

cDNA obtained from the reverse transcription of RNA extracted was submitted to RT-qPCR to see the levels of btn1 mutant transcripts using primers in Table 7. The housekeeping gene actin is used as a reference. Data was analysed using the 2^−∆∆C(T)^ method^39^.

### Generation of double mutant libraries

Three independent biological replicates of each SGA were performed with the Bioneer V5 deletion collection^40^ using a ROTOR HDA pinning robot (Singer Instruments) as previously described^41^. Briefly, the three query strains *h-btn1::NatMX ura-D18 leu1-32 ade6-M21*0, *h-btn1*^*102–208del*^*::NatMX ura-D18 leu1-32 ade6-M210*, *h-btn1*^*D363G*^*::NatMX ura-D18 leu1-32 ade6-M210* and a control query strain, *h-ade6::NatMX ura-D18 leu1-32 ade6-M210*^42^ were mated with the library on Edinburgh Minimal Medium without nitrogen (Formedium). Following sporulation for 3 days at 25 °C and spore selection for 3 days at 42 °C, spores were pinned onto YES agar for 2 days at 32 °C to recover. Double mutant haploids were selected by growing cells on YES agar with ClonNat (Jena Bioscience; AB-102XL; 100 μg/ml) and G418 (Formedium; 500 μg/ml) for 2 days at 32 °C. Double mutant libraries were then grown on YES agar in quadruplicate (1536-well format) for 2 days at 32 °C.

### Image analysis/processing

Images of colonies were acquired as shown (Fig. 1A). Plates were imaged using an EPSON Expression 1680 Pro transmission scanner. The size of each colony was determined using the *gitter* package in R^43^.

Mutants were excluded from the analysis if they failed to grow on the control *ade6* plates. This was determined independently for each batch. Colony size was normalised to plate median, row and column median and for spatial effects as described previously^44^. Library mutants which were located with 500 kb of a query mutation were also excluded in order to remove spurious interactions as a result of linkage. Batch effects were removed using the *limma* package prior to Principal Component Analysis^45^. Differential fitness analysis was performed using *limma*^45^, instead using the plate normalised colony sizes and including batch as a term in the model. In order to account for differences in variance as a function of colony size, we model variance as a function of colony size in an approach akin to *limma-voom*^46^. Given that each library mutant was pinned in quadruplicate on the same plate and are hence not truly independent, each quadruplicate was treated as a technical replicate. The correlation between technical replicates was calculated, akin to calculating the correlation between duplicate spots in microarrays, and a mixed linear model was fitted to the data^47^. For each contrast of interest, p-values were corrected according to Benjamini–Hochberg^48^.

Cluster analysis was performed using *Cluster3.0* for normalised colony sizes of each strain (http://bonsai.hgc.jp/~mdehoon/software/cluster/software.htm#ctv)^49^ and visualised with *Java-Treeview* (http://jtreeview.sourceforge.net)^50^.

### Network analysis

Using Cytoscape version 3.4.0 (http://www.cytoscape.org/), gene lists of the most consistent hits were further analysed using add on macros for ClueGO version 2.3.3 (http://apps.cytoscape.org/apps/cluego) and CluePedia version 1.3.3 (http://apps.cytoscape.org/apps/cluepedia) for GO annotations, biological processes and KEGG pathways^51,52^. Kappa score of 0.4. Significant genetic interactions were compared to pombase’s slim Monarch Disease Ontology (Mondo) (https://www.pombase.org/browse-curation/disease-slim) for genes annotated to human disease.

## Supplementary Information

S1: Supplementary Figures 1.

S2: Supplementary Tables 2.

S3: Supplementary Tables 3.

S4: Supplementary Tables 4.

S5: Supplementary Tables 5.

S6: Supplementary Tables 6.

S7: Supplementary Tables 7.

S8: Supplementary Tables 8.

S9: Supplementary Tables 9.

S10: Supplementary Tables 10.

S11: Supplementary Table 11.

S12: Supplementary Table 12.

S13: Supplementary Table 13.
