# Author Correction: Global network analysis in *Schizosaccharomycespombe* reveals three distinct consequences of the common 1-kb deletion causing juvenile CLN3 disease

**DOI:** 10.1038/s41598-021-93446-8

**Published:** 2021-07-05

**Authors:** Christopher J. Minnis, StJohn Townsend, Julia Petschnigg, Elisa Tinelli, Jürg Bähler, Claire Russell, Sara E. Mole

**Affiliations:** 1 MRC Laboratory for Molecular Cell Biology and Great Ormond Street, Institute of Child Health, University College London, London, WC1E 6BT UK; 2 Department of Comparative Biomedical Sciences, Royal Veterinary College, Royal College Street, London, NW1 0TU UK; 3 Institute of Healthy Ageing, Department of Genetics, Evolution and Environment, University College London, London, WC1E 6BT UK; 4 The Molecular Biology of Metabolism Laboratory, The Francis Crick Institute, London, NW1 1AT UK

Correction to: *Scientific Reports* 10.1038/s41598-021-85471-4, published online 18 March 2021

The original version of this Article contained errors in Figure 1c, where data points in the bar graph plot appeared incorrectly in green colour. As a results, the Figure legend,

“General overview of SGA analysis of *btn1* mutants versus *ade6* control. (**A**) Representative images of the SGA plates for control (*ade6*) and query mutants (*btn1Δ, btn1*^*D363G*^*, btn1*^*102–208del*^), with empty control quadrants shown for *ade6* (yellow boxes). (**B**) Exclusion of small colonies for *ade6* control across batches as they represent high variability therefore reducing noise. (**C**) Principle component biplot of the variance within the SGA data for *ade6* control (yellow*)* and query-mutants *btn1*Δ (blue), *btn1*^*D363G*^ (green), *btn1*^*102–208del*^ (red), with experimental batch indicated. (**D**) Cluster analysis for each strain and all the genes with their normalised colony size difference against *ade6* control with batch effects removed. Interactions are coloured in blue for negative interactions (< − 0.5) and yellow for positive interactions (> 0.5). (**E**) Gene linkage of normalised fitness score for *ade6* control and query mutants *btn1Δ*, *btn1*^*D363G*^, *btn1*^*102–208del*^ from one experiment. Vertical dashed line represents *ade6* or *btn1* gene location, red points represent interaction scores excluded from data since less than 500 kb/500,000 bps from query gene location.”

now reads:

“General overview of SGA analysis of *btn1* mutants versus *ade6* control. (**A**) Representative images of the SGA plates for control (*ade6*) and query mutants (*btn1Δ, btn1*^*D363G*^*, btn1*^*102–208del*^), with empty control quadrants shown for *ade6* (yellow boxes). (**B**) Exclusion of small colonies for *ade6* control across batches as they represent high variability therefore reducing noise. (**C**) Principle component biplot of the variance within the SGA data for *ade6* control (yellow*)* and query-mutants *btn1*Δ (blue), *btn1*^*D363G*^ (orange), *btn1*^*102–208del*^ (red), with experimental batch indicated. (**D**) Cluster analysis for each strain and all the genes with their normalised colony size difference against *ade6* control with batch effects removed. Interactions are coloured in blue for negative interactions (< − 0.5) and yellow for positive interactions (> 0.5). (**E**) Gene linkage of normalised fitness score for *ade6* control and query mutants *btn1∆*, *btn1*^*D363G*^, *btn1*^*102–208del*^ from one experiment. Vertical dashed line represents *ade6* or *btn1* gene location, red points represent interaction scores excluded from data since less than 500 kb/500,000 bps from query gene location.”

The original Figure 1 and accompanying legend appear below.

Furthermore, Table 2 contained errors, where genes for the strains *btn1∆, btn1*^*D363G*^ and *btn1*^*102–208del*^ in the column ‘Top 5 genes’ appeared incorrectly in bold font. The incorrect and correct values appear below.

Incorrect:

**Table:** 

Strains	Interaction type	Top 5 genes
*btn1*∆	negative	***pfa3****, ****kes1***, ***rid1, efc25***, *ivn1*
	positive	***apt1***, ***blt1*, *cpp1****, rpl2301, alg12*
*btn1*^*D363G*^	negative	***pfa3, rid1, kes1, efc25, fhn1***
	positive	*grx5, cpp1, SPBC1604.03c, apt1, ****blt1***
*btn1*^*102-208del*^	negative	***pfa3, nce103, gga22, clg1, kes1***
	positive	*SPBC29A3.21, cpp1, gfh1, tfx1, pub3*

Correct:

**Table:** 

Strains	Interaction type	Top 5 genes
*btn1*∆	negative	***pfa3****, ****kes1***, *rid1, ****efc25***, ***ivn1***
	positive	***apt1***, *blt1*, ***cpp1****, rpl2301, ****alg12***
*btn1*^*D363G*^	negative	***pfa3****, rid1, ****kes1****, ****efc25****, fhn1*
	positive	*grx5, ****cpp1****, SPBC1604.03c, ****apt1****, blt1*
*btn1*^*102-208del*^	negative	***pfa3****, nce103, gga22, clg1, ****kes1***
	positive	*SPBC29A3.21, ****cpp1****, gfh1, tfx1, pub3*

**Figure 1:**
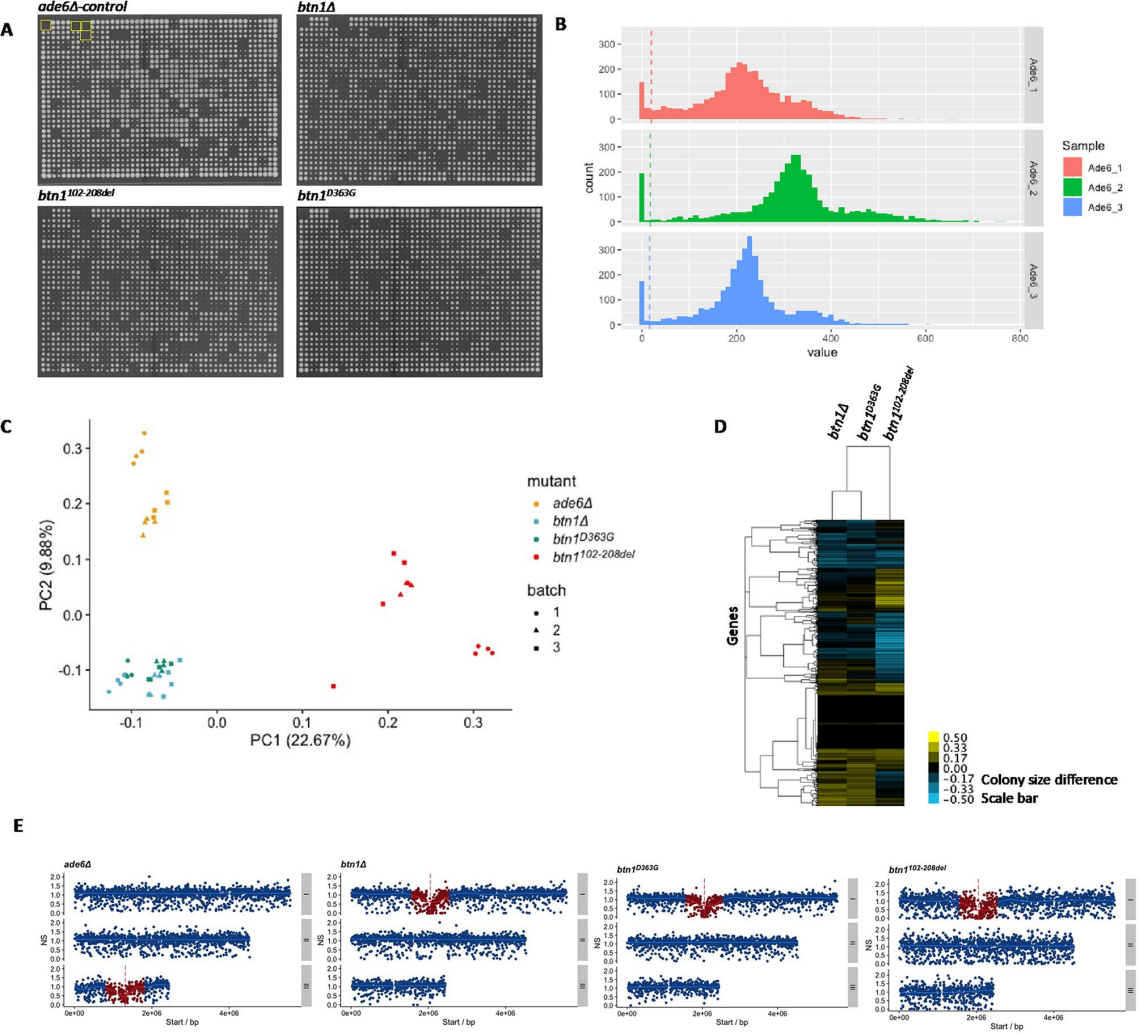
General overview of SGA analysis of *btn1* mutants versus *ade6* control. (**A**) Representative images of the SGA plates for control (*ade6*) and query mutants (*btn1Δ, btn1*^*D363G*^*, btn1*^*102–208del*^), with empty control quadrants shown for *ade6* (yellow boxes). (**B**) Exclusion of small colonies for *ade6* control across batches as they represent high variability therefore reducing noise. (**C**) Principle component biplot of the variance within the SGA data for *ade6* control (yellow*)* and query-mutants *btn1*Δ (blue), *btn1*^*D363G*^ (green), *btn1*^*102–208del*^ (red), with experimental batch indicated. (**D**) Cluster analysis for each strain and all the genes with their normalised colony size difference against *ade6* control with batch effects removed. Interactions are coloured in blue for negative interactions (< − 0.5) and yellow for positive interactions (> 0.5). (**E**) Gene linkage of normalised fitness score for *ade6* control and query mutants *btn1∆*, *btn1*^*D363G*^, *btn1*^*102–208del*^ from one experiment. Vertical dashed line represents *ade6* or *btn1* gene location, red points represent interaction scores excluded from data since less than 500 kb/500,000 bps from query gene location.

The original Article has been corrected.

