# Supplementary material for: Global network analysis in Schizosaccharomyces pombe reveals three distinct consequences of the common 1-kb deletion causing juvenile CLN3 disease
Source: Sci Rep. 2021 Mar 18;11:6332. doi: 10.1038/s41598-021-85471-4 (PMC7973434; doi:10.1038/s41598-021-85471-4)
Supplement: Supplementary file 1 — S1: Supplementary Figures 1. [file 41598_2021_85471_MOESM1_ESM.docx]

Global network analysis in *Schizosaccharomyces pombe* reveals three distinct consequences of the common 1-kb deletion causing juvenile CLN3 disease

Christopher J. Minnis^1,2^, StJohn Townsend^3,4^, Julia Petschnigg^1^, Elisa Tinelli^1^, Jürg Bähler^3^, Claire Russell^2^, Sara E. Mole^1^

*^1^MRC Laboratory for Molecular Cell Biology and Great Ormond Street Institute of Child Health, University College London, London WC1E 6BT, UK*

*^2^Dept. Comparative Biomedical Sciences, Royal Veterinary College, Royal College Street, London NW1 0TU, UK*

*^3^Institute of Healthy Ageing, Department of Genetics, Evolution and Environment, University College London, London WC1E 6BT, UK*

*^4^The Molecular Biology of Metabolism Laboratory, The Francis Crick Institute, London, NW1 1AT, United Kingdom*

*Corresponding author: [christopher.minnis.15@ucl.ac.uk](mailto:christopher.minnis.15@ucl.ac.uk)

Figure 1: **SGA workflow to cross the query gene *btn1*** **mutants with the Bioneer deletion library of non-essential genes**. **A**) A query strain (*h^-^*) of interest *btn1* mutant is mated to the Bioneer deletion library of non-essential gene strains (*h^+^*) in an ordered array. **B**) Diploids are allowed to sporulate and germinate through a number of culturing and transferring steps. **C**) Double mutant progeny are selected by antibiotic resistance selection imparted by cassettes present in the query gene btn1 strain (*btn1::NatMX* cassette) and Bioneer library (*gene::G418* cassette). **D**) Double mutants are then allowed to grow for two days before plates are imaged and scored for their colony fitness. **E, F**) Genetic interactions are identified using colony size as a proxy for altered growth rates of the meiotic progeny. The fitness of the progeny is determined to be either positive/suppressors (bigger colony; green), negative synthetic lethality/sickness enhancers (absent/small colony; red) or no effect (same size). Created with BioRender.com


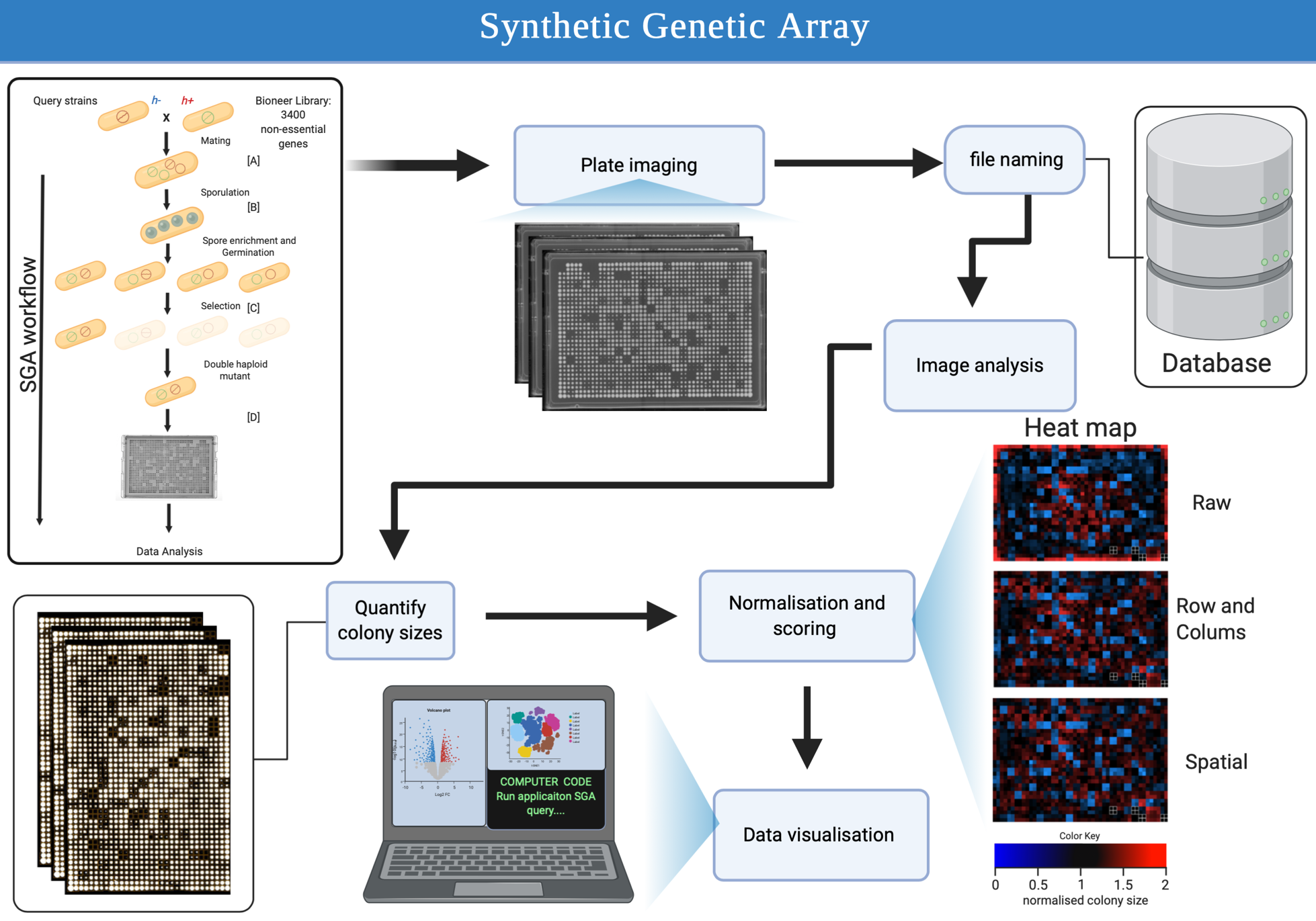
Figure 2: **SGA processing workflow**: **A)** SGA experiment. **B)** Digitization of plates and orientation adjustments in ImageJ. **C)** Quantification using R script Gitter for colony sizes. **D)** Normalization of plates and fitness scoring. **E)** Data analysis and visualization of results. (Adapted from <http://sgatools.ccbr.utoronto.ca>), created with BioRender.com

Figure 3: Genetic network of *btn1^102-208del^* and GO term enrichment for positive and negative interactions against ade6 control. A) Genetic networks of *ade6* control and *btn1^102-208del^* represented in terms of positive (green) and negative (red) interactions. Grey GO terms represent terms with both interactions, with a scale of red or green dependent on the number of interactions associated with that specific GO term. B) Represents the significant GO term ribosome within this genetic network between *ade6* control and *btn1^102-208del^*. C-D) Represents a sideways bar-chart of negative and positive interactions respectively between *ade6* and *btn1^102-208del^*, the graphs correspond to the contribution of the genes associated with that specific term in percentiles along with the number of genes enriched in the analysis, C’-D’) A pie chart representing the number of associated GO terms per similar groups. Generated with ClueGO in Cytoscape.

Figure 4: **Schematic of the predicted topology of Btn1 protein, transcript sequence alignment and mutant expression profile**. **A)** This predicted topology is based on how the mutants were generated. Btn1 is a transmembrane protein with the N- & C- terminals protruding into the cytoplasm. *Btn1^102-208del^* and *btn1^D363G^* mutation location are annotated in red and orange respectively, created with BioRender.com. **B)** Transcriptional sequence of btn1, with mutation loci on the sequence 1kb deletion equivalent to 102-208aa deletion (turquoise) and missense mutation (Asp363Gly), in addition locations of primers used to assess RNA expression. **C)** Comparison of expression levels of Btn1 proteins in mutant lines *btn1∆*, *btn1^D363G^* and *btn1^102-208del^* against WT strain using the 2^-∆∆C(T)^ method with actin as our control. Both Btn1^D363G^ and Btn1^102-208del^ are expressed at higher levels than endogenous Btn1.
