# Supplementary material for: Global network analysis in Schizosaccharomyces pombe reveals three distinct consequences of the common 1-kb deletion causing juvenile CLN3 disease
Source: Sci Rep. 2021 Mar 18;11:6332. doi: 10.1038/s41598-021-85471-4 (PMC7973434; doi:10.1038/s41598-021-85471-4)
Supplement: Supplementary file 2 — S2: Supplementary Tables 2. [file 41598_2021_85471_MOESM2_ESM.pdf]

Global network analysis in *Schizosaccharomyces pombe* reveals three distinct consequences of the common 1-kb deletion causing juvenile CLN3 disease

Christopher J. Minnis[1,2], StJohn Townsend[3,4], Julia Petschnigg[1], Elisa Tinelli[1], Jürg Bähler[3], Claire Russell[2], Sara E. Mole[1]

[1]MRC Laboratory for Molecular Cell Biology and Great Ormond Street Institute of Child Health, University College London, London WC1E 6BT, UK
[2]Dept. Comparative Biomedical Sciences, Royal Veterinary College, Royal College Street, London NW1 0TU, UK
[3]Institute of Healthy Ageing, Department of Genetics, Evolution and Environment, University College London, London WC1E 6BT, UK
[4]The Molecular Biology of Metabolism Laboratory, The Francis Crick Institute, London, NW1 1AT, United Kingdom

*Corresponding author: christopher.minnis.15@ucl.ac.uk

Supplymentary table 1: Negative genetic interactions for *btn1*Δ vs *ade6*Δ control

Systematic ID	Gene name	Product description	Colony Size Difference	t	P Value	Adjusted P Value
SPBC2F12.15c	pfa3	palmitoyltransferase Pfa3 (predicted)	-0.88	-17.46	4.28E-22	1.11E-18
SPBC1271.12	kes1	sterol transfer protein Kes1 (predicted)	-0.6	-8.93	1.15E-11	1.50E-08
SPBC1709.12	rid1	GTPase binding protein Rid1 (predicted)	-0.44	-7.63	9.63E-10	8.34E-07
SPBC336.03	efc25	Ras1 GEF Efc25	-0.45	-7.04	7.27E-09	4.73E-06
SPBC11B10.07c	ivn1	plasma membrane phospholipid-translocating ATPase complex Lem3 family subunit Ivn1 (predicted)	-0.38	-6.16	1.60E-07	5.21E-05
SPCC576.13	swc5	Swr1 complex subunit Swc5	-0.5	-5.6	1.11E-06	2.22E-04
SPBC337.08c	ubi4	protein modifier, ubiquitin	-1.17	-5.48	1.69E-06	3.13E-04
SPAC11G7.01	mtl2	plasma membrane-associated serine-rich cell wall sensor Mtl2	-0.34	-5.12	5.65E-06	7.73E-04
SPCP1E11.05c	are2	acyl-coA-sterol acyltransferase Are2	-0.27	-5.06	7.01E-06	9.11E-04
SPBC25H2.16c	gga22	Golgi localized Arf binding gamma-adaptin ortholog Gga22	-0.28	-5.01	8.21E-06	1.02E-03
SPBC13A2.02	nup82	nucleoporin, WD repeat Nup82	-0.39	-4.72	2.20E-05	2.29E-03
SPAC4H3.13	pcc1	EKC/KEOPS complex subunit Pcc1 (predicted)	-0.53	-4.66	2.69E-05	2.69E-03
SPAC4G8.10	gos1	SNARE Gos1 (predicted)	-0.29	-4.61	3.09E-05	2.87E-03
SPBC3F6.01c	SPBC3F6.01c	TPR repeat serine/threonine protein phosphatase (predicted)	-0.31	-4.61	3.09E-05	2.87E-03
SPCC126.15c	sec65	signal recognition particle subunit Sec65 (predicted)	-0.47	-4.59	3.36E-05	3.02E-03
SPAC11D3.04c	SPAC11D3.04c	polyketide cyclase SnoaL-like domain protein	-0.5	-4.53	4.09E-05	3.43E-03
SPBP8B7.05c	nce103	carbonic anhydrase (predicted)	-0.34	-4.47	4.96E-05	3.91E-03
SPACUNK4.11c	mpp6	nuclear exosome-associated RNA binding protein Mpp6	-0.44	-4.38	6.73E-05	5.00E-03
SPBC106.02c	srx1	sulfiredoxin	-0.34	-4.35	7.25E-05	5.23E-03
SPBC26H8.14c	cox17	mitochondrial copper chaperone for cytochrome c oxidase Cox17 (predicted)	-0.38	-4.33	7.83E-05	5.50E-03
SPBC646.13	sds23	PP2A-type phosphatase inhibitor Sds23/Moc1	-0.43	-4.3	8.67E-05	5.85E-03
SPCC548.04	urm1	ubiquitin-like protein modifier Urm1 (predicted)	-0.39	-4.27	9.42E-05	5.85E-03
SPAC458.05	pik3	phosphatidylinositol 3-kinase Pik3	-0.47	-4.17	1.31E-04	7.11E-03
SPBC2D10.06	rep1	MBF transcription factor activator Rep1	-0.29	-4.19	1.23E-04	7.11E-03
SPBC902.06	mto2	gamma tubulin complex linker Mto2	-0.29	-4.17	1.29E-04	7.11E-03
SPBC428.03c	pho4	thiamine-repressible acid phosphatase Pho4	-0.28	-4.05	1.92E-04	9.43E-03
SPBC337.09	erg28	Erg28 protein (predicted)	-0.23	-4.06	2.08E-04	9.82E-03
SPBC26H8.08c	grn1	GTPase Grn1	-0.45	-3.99	2.32E-04	1.04E-02
SPBC23E6.01c	cxr1	splicing factor Cxr1	-0.26	-3.96	2.56E-04	1.11E-02
SPBC12D12.05c	SPBC12D12.05c	mitochondrial carrier, ATP:ADP antiporter (predicted)	-0.24	-3.89	3.18E-04	1.25E-02
SPBC1734.05c	spf31	DNAJ protein, splicing factor Spf31 (predicted)	-0.3	-3.89	3.13E-04	1.25E-02
SPBC216.06c	swi1	replication fork protection complex subunit Swi1	-0.36	-3.79	4.27E-04	1.55E-02
SPBC31F10.07	lsb5	actin cortical patch component Lsb5 (predicted)	-0.33	-3.8	4.19E-04	1.55E-02
SPAC3H8.09c	nab3	poly(A) binding protein Nab3 (predicted)	-0.26	-3.74	4.97E-04	1.68E-02
SPBC13E7.08c	leo1	RNA polymerase II associated Paf1 complex subunit Leo1	-0.21	-3.74	5.00E-04	1.68E-02
SPAC139.01c	mkt1	post-transcriptional RNA stability regulator Mkt1	-0.26	-3.72	5.31E-04	1.69E-02
SPBC11C11.02	imp2	F-BAR domain protein Imp2	-0.31	-3.72	5.33E-04	1.69E-02
SPAC1486.08	cox16	mitochondrial copper chaperone for cytochrome c oxidase Cox16 (predicted)	-0.26	-3.62	7.33E-04	2.21E-02

SPAC823.03	ppk15	serine/threonine protein kinase Ppk15 (predicted)	-0.44	-3.6	7.65E-04	2.26E-02
SPCC1620.04c	fzr3	meiotic fizzy-related APC coactivator Fzr3	-0.21	-3.59	7.87E-04	2.28E-02
SPBC25H2.08c	mrs2	mitochondrial inner membrane magnesium ion transmembrane transporter Mrs2 (predicted)	-0.19	-3.57	8.43E-04	2.36E-02
SPAC4C5.04	rad31	SUMO activating enzyme E1-type Rad31	-0.35	-3.55	8.81E-04	2.41E-02
SPAC2C4.07c	dis32	3'-5'-exoribonuclease activity Dis3L2	-0.2	-3.54	9.32E-04	2.46E-02
SPBC21C3.11	ubx4	UBX domain protein Ubx4 (predicted)	-0.33	-3.56	9.30E-04	2.46E-02
SPBC26H8.05c	ppe2	serine/threonine protein phosphatase, PP4 complex subunit Ppe2	-0.24	-3.53	9.35E-04	2.46E-02
SPAC1071.03c	sil1	nucleotide exchange factor for the ER lumenal Hsp70 chaperone, Sil1 (predicted)	-0.19	-3.53	9.45E-04	2.46E-02
SPAC922.05c	SPAC922.05c	transmembrane transporter (predicted)	-0.24	-3.52	9.65E-04	2.48E-02
SPACUNK4.10	gor1	glyoxylate reductase (predicted)	-0.38	-3.49	1.05E-03	2.63E-02
SPAC22H12.05c	fsc1	fasciclin domain protein Fsc1	-0.25	-3.48	1.09E-03	2.68E-02
SPAC26A3.14c	SPAC26A3.14c	DUF1748 family protein	-0.23	-3.45	1.18E-03	2.85E-02
SPAC513.03	mfm2	M-factor precursor Mfm2	-0.43	-3.4	1.39E-03	3.23E-02
SPAC630.04c	SPAC630.04c	Schizosaccharomyces specific protein	-0.26	-3.39	1.43E-03	3.23E-02
SPBC11B10.10c	pht1	histone H2A variant H2A.Z, Pht1	-0.2	-3.39	1.43E-03	3.23E-02
SPBC29A3.07c	sap14	U2 snRNP-associated protein SF3B14 Sap14	-0.28	-3.39	1.44E-03	3.23E-02
SPBC16E9.09c	erp5	COPII vesicle coat component Erp5/Erp6 (predicted)	-0.24	-3.38	1.46E-03	3.25E-02
SPBC800.05c	atb2	tubulin alpha 2	-0.27	-3.35	1.59E-03	3.47E-02
SPBC30D10.13c	pdb1	pyruvate dehydrogenase e1 component beta subunit Pdb1	-0.4	-3.35	1.61E-03	3.48E-02
SPAC3G6.04	rnp24	RNA-binding protein Rnp24	-0.35	-3.33	1.69E-03	3.59E-02
SPCC70.08c	SPCC70.08c	methyltransferase (predicted)	-0.38	-3.31	1.78E-03	3.73E-02
SPAC19G12.08	scs7	ER sphingosine hydroxylase Scs7	-0.26	-3.29	1.94E-03	3.84E-02
SPAC23C4.09c	SPAC23C4.09c	DNA-binding TFAR19-related protein, human Programmed cell death protein 5 ortholog (predicted)	-0.2	-3.29	1.93E-03	3.84E-02
SPAC4C5.02c	ryh1	GTPase Ryh1	-0.29	-3.27	2.01E-03	3.93E-02
SPCC1494.08c	SPCC1494.08c	cortical variant C2 domain protein, human FAM102A and FAM102B ortholog, implicated in signalling or endocytosis	-0.17	-3.27	2.03E-03	3.93E-02
SPAC24H6.13	SPAC24H6.13	calcium permeable stress-gated cation channel (predicted)	-0.29	-3.26	2.09E-03	3.99E-02
SPAC25B8.06c	dia4	mitochondrial serine-tRNA ligase (predicted)	-0.36	-3.26	2.09E-03	3.99E-02
SPAC3A11.08	pcu4	cullin 4	-0.36	-3.25	2.13E-03	4.02E-02
SPAC23G3.08c	ubp7	ubiquitin C-terminal hydrolase Ubp7	-0.19	-3.23	2.39E-03	4.39E-02
SPAC637.03	SPAC637.03	DUF1774 family multi-spanning conserved fungal membrane protein	-0.2	-3.18	2.60E-03	4.72E-02

Supplymentary table 2: Positive genetic interactions for *btn1*Δ vs *ade6*Δ control

Systematic ID	Gene name	Product description	Colony Size Difference	t	P Value	Adjusted P Value
SPAC23A1.03	apt1	adenine phosphoribosyltransferase (APRT) Apt1	0.41	6.83	2.39E-08	1.24E-05
SPBC1A4.05	blt1	ubiquitin domain-like protein Blt1	0.53	6.36	7.90E-08	3.42E-05
SPAC17G6.04c	cpp1	protein farnesyltransferase beta subunit Cpp1	0.36	6.23	1.22E-07	4.53E-05
SPAC3G9.03	rpl2301	60S ribosomal protein L23	0.36	5.98	3.00E-07	8.63E-05
SPBC1734.12c	alg12	dolichyl pyrophosphate Man7GlcNAc2 alpha-1,6-mannosyltransferase Alg12 (predicted)	0.32	5.95	3.32E-07	8.63E-05
SPAPB2B4.02	grx5	mitochondrial [2Fe-2S] cluster assembly and transfer glutaredoxin Grx5	0.25	5.99	3.91E-07	9.24E-05
SPAC6G9.12	cfr1	exomer complex BRCT domain subunit Cfr1	0.33	5.87	4.31E-07	9.35E-05
SPCC18B5.10c	tex1	TREX complex subunit Tex1 (predicted)	0.51	5.41	2.08E-06	3.60E-04
SPAC1486.04c	alm1	nucleoporin Alm1	0.26	5.4	2.78E-06	4.51E-04
SPCC320.06	SPCC320.06	conserved fungal protein	0.31	5.22	4.00E-06	6.11E-04
SPCC1393.08	fil1	transcription factor, zf-GATA type	0.35	5.16	4.91E-06	7.10E-04
SPAC144.11	rps1102	40S ribosomal protein S11 (predicted)	0.29	4.93	1.08E-05	1.27E-03
SPAC16.03c	ura2	dihydroorotase Ura2	0.32	4.73	2.10E-05	2.27E-03
SPBC887.17	SPBC887.17	nucleobase transmembrane transporter (predicted)	0.47	4.74	2.04E-05	2.27E-03
SPAC1834.05	alg9	mannosyltransferase complex subunit Alg9 (predicted)	0.25	4.62	3.57E-05	3.09E-03
SPBC19F8.08	rps401	40S ribosomal protein S4 (predicted)	0.29	4.49	4.66E-05	3.79E-03
SPCC613.03	SPCC613.03	endoplasmic reticulum EF hand protein (predicted)	0.33	4.4	6.24E-05	4.77E-03
SPAPJ696.01c	vps17	retromer complex subunit Vps17	0.48	4.27	9.44E-05	5.85E-03
SPBC839.13c	rpl1601	60S ribosomal protein L13/L16 (predicted)	0.26	4.28	9.20E-05	5.85E-03
SPCPB1C11.01	amt1	plasma membrane ammonium transmembrane transporter Amt1	0.32	4.29	9.00E-05	5.85E-03
SPAC323.05c	mtq2	eRF1 methyltransferase Mtq2 (predicted)	0.22	4.22	1.12E-04	6.75E-03
SPAC19B12.11c	bud20	zinc finger ribosome biogenesis protein Bud20 (predicted)	0.25	4.21	1.28E-04	7.11E-03
SPBP16F5.05c	yar1	ribosome biogenesis protein Yar1 (predicted)	0.31	4.18	1.25E-04	7.11E-03
SPBC1711.05	srp40	nucleocytoplasmic transport chaperone Srp40 (predicted)	0.41	4.1	1.65E-04	8.77E-03
SPAC16E8.01	shd1	cytoskeletal protein binding protein Sla1 family, Shd1 (predicted)	0.25	4.08	1.74E-04	9.05E-03
SPCC330.14c	rpl2402	60S ribosomal protein L24 (predicted)	0.28	4.06	1.83E-04	9.33E-03
SPBP4H10.09	rsv1	transcription factor Rsv1	0.45	4.06	1.87E-04	9.35E-03
SPBC1711.03	emc3	ER membrane protein complex subunit Emc3 (predicted)	0.64	4.03	2.02E-04	9.74E-03
SPAC144.04c	spe1	ornithine decarboxylase Spe1 (predicted)	0.25	3.98	2.35E-04	1.04E-02
SPAC1F3.03	sro7	Lgl family protein Sro7 (predicted)	0.36	4	2.27E-04	1.04E-02
SPCC330.19c	SPCC330.19c	Schizosaccharomyces pombe specific protein	0.25	3.98	2.35E-04	1.04E-02
SPCC1919.10c	myo52	myosin type V	0.33	3.93	2.81E-04	1.20E-02
SPBC577.02	rpl3801	60S ribosomal protein L38 (predicted)	0.23	3.92	3.14E-04	1.25E-02
SPBP35G2.08c	air1	TRAMP complex zinc knuckle subunit Air1	0.23	3.9	3.10E-04	1.25E-02
SPCC794.15	SPCC794.15	Schizosaccharomyces specific protein	0.27	3.92	3.12E-04	1.25E-02
SPBC1604.03c	SPBC1604.03c	conserved fungal protein, implicated in vesicle trafficking or lipid metabolism	0.2	3.86	3.44E-04	1.33E-02
SPCC1235.03	cue2	no go decay endonuclease Cue2	0.23	3.82	3.94E-04	1.51E-02
SPAC13G6.10c	asl1	cell wall protein Asl1, predicted O-glucosyl hydrolase	0.44	3.79	4.29E-04	1.55E-02
SPCC594.06c	vsl1	vacuolar SNARE Vsl1/Vam7	0.26	3.8	4.22E-04	1.55E-02
SPCC330.11	btb1	BTB/POZ domain protein Btb1	0.29	3.78	4.40E-04	1.57E-02
SPBC3D6.04c	mad1	mitotic spindle checkpoint protein Mad1	0.28	3.77	4.64E-04	1.63E-02
SPCC1223.05c	rpl3702	60S ribosomal protein L37 (predicted)	0.28	3.76	4.79E-04	1.66E-02

SPCC285.15c	rps2802	40S ribosomal protein S28, Rps2802	0.28	3.74	5.04E-04	1.68E-02
SPAC31G5.03	rps1101	40S ribosomal protein S11 (predicted)	0.23	3.73	5.20E-04	1.69E-02
SPAPB17E12.08	eos1	N-glycosylation protein Eos1 (predicted)	0.24	3.75	5.22E-04	1.69E-02
SPBC1711.15c	SPBC1711.15c	Schizosaccharomyces pombe specific protein	0.31	3.69	6.26E-04	1.96E-02
SPBC16H5.12c	SPBC16H5.12c	DUF2433 metallo phosphatase superfamily conserved fungal protein	0.34	3.65	6.51E-04	2.02E-02
SPBC2G2.01c	liz1	plasma membrane pantothenate transmembrane transporter Liz1	0.19	3.64	6.89E-04	2.11E-02
SPBC1734.13	atp3	F1-FO ATP synthase gamma subunit (predicted)	0.19	3.6	7.57E-04	2.26E-02
SPBC1734.08	hse1	STAM like protein Hse1	0.24	3.59	7.89E-04	2.28E-02
SPCC1739.14	npp106	nucleoporin Npp106	0.23	3.58	8.06E-04	2.30E-02
SPAC926.05c	dph4	diphthamide biosynthesis protein Dph4 (predicted)	0.22	3.58	8.16E-04	2.30E-02
SPAC30D11.10	rad52	DNA recombination protein, Rad51 mediator Rad52 (previously Rad22)	0.21	3.56	8.59E-04	2.38E-02
SPBC776.11	rpl2801	60S ribosomal protein L27/L28	0.22	3.54	9.07E-04	2.46E-02
SPAC30D11.05	aps3	AP-3 adaptor complex subunit Aps3 (predicted)	0.19	3.52	9.79E-04	2.50E-02
SPCC594.04c	SPCC594.04c	steroid oxidoreductase superfamily protein (predicted)	0.29	3.51	9.92E-04	2.50E-02
SPBC9B6.03	SPBC9B6.03	zf-FYVE type zinc finger protein, involved in endosomal transport	0.28	3.48	1.09E-03	2.68E-02
SPBC8D2.04	hht2	histone H3 h3.2	0.22	3.46	1.17E-03	2.85E-02
SPBC16C6.03c	rsa1	ribosome assembly protein Rsa1 (predicted)	0.16	3.43	1.27E-03	3.02E-02
SPAC19D5.11c	ctf8	Ctf18 RFC-like complex subunit Ctf8	0.22	3.43	1.37E-03	3.23E-02
SPCC320.05	SPCC320.05	sulfate transmembrane transporter (predicted)	0.25	3.39	1.44E-03	3.23E-02
SPCC757.02c	SPCC757.02c	dehydrogenase (predicted)	0.17	3.39	1.42E-03	3.23E-02
SPBC1921.01c	rpl35b	60S ribosomal protein L35a (predicted)	0.2	3.37	1.53E-03	3.38E-02
SPCP20C8.02c	SPCP20C8.02c	S. pombe specific UPF0321 family protein 1	0.24	3.34	1.64E-03	3.51E-02
SPAC328.10c	rps502	40S ribosomal protein S5 (predicted)	0.19	3.35	1.70E-03	3.59E-02
SPAC644.15	rpp101	60S acidic ribosomal protein A1	0.18	3.31	1.83E-03	3.74E-02
SPCC1682.14	rpl1902	60S ribosomal protein L19	0.25	3.31	1.81E-03	3.74E-02
SPCP31B10.07	eft202	translation elongation factor 2 (EF-2) Eft2,B	0.17	3.31	1.83E-03	3.74E-02
SPCC736.09c	tfx1	TRAX	0.19	3.3	1.86E-03	3.78E-02
SPCC553.01c	dbl2	DNA recombination protein Dbl2	0.19	3.29	1.89E-03	3.81E-02
SPAC13G6.02c	rps101	40S ribosomal protein S3a	0.2	3.28	1.98E-03	3.89E-02
SPAC29A4.18	prw1	Clr6 histone deacetylase complex subunit Prw1	0.22	3.25	2.11E-03	4.01E-02
SPBC18H10.19	vps38	phophatidylinositol 3-kinase complex subunit Vps38	0.19	3.22	2.35E-03	4.39E-02
SPBC215.14c	vps20	ESCRT III complex subunit Vps20	0.2	3.21	2.40E-03	4.39E-02
SPCC1235.06	get2	GET complex (ER membrane insertion) subunit Get2	0.24	3.21	2.39E-03	4.39E-02
SPAC26H5.05	mga2	IPT/TIG ankyrin repeat gene-specific transcription coactivator Mga2	0.35	3.16	2.74E-03	4.96E-02
