# Supplementary material for: Global network analysis in Schizosaccharomyces pombe reveals three distinct consequences of the common 1-kb deletion causing juvenile CLN3 disease
Source: Sci Rep. 2021 Mar 18;11:6332. doi: 10.1038/s41598-021-85471-4 (PMC7973434; doi:10.1038/s41598-021-85471-4)
Supplement: Supplementary file 3 — S3: Supplementary Tables 3. [file 41598_2021_85471_MOESM3_ESM.pdf]

Global network analysis in *Schizosaccharomyces pombe* reveals three distinct consequences of the common 1-kb deletion causing juvenile CLN3 disease

Christopher J. Minnis[1,2], StJohn Townsend[3,4], Julia Petschnigg[1], Elisa Tinelli[1], Jürg Bähler[3], Claire Russell[2], Sara E. Mole[1]

[1]MRC Laboratory for Molecular Cell Biology and Great Ormond Street Institute of Child Health, University College London, London WC1E 6BT, UK
[2]Dept. Comparative Biomedical Sciences, Royal Veterinary College, Royal College Street, London NW1 0TU, UK
[3]Institute of Healthy Ageing, Department of Genetics, Evolution and Environment, University College London, London WC1E 6BT, UK
[4]The Molecular Biology of Metabolism Laboratory, The Francis Crick Institute, London, NW1 1AT, United Kingdom

*Corresponding author: christopher.minnis.15@ucl.ac.uk

Supplementary table 1: Negative genetic interactions for *btn1D363G* vs *ade6*Δ control

Systematic ID	Gene name	Product description	Colony Size Difference	t	P Value	Adjusted P Value
SPBC2F12.15c	pfa3	palmitoyltransferase Pfa3 (predicted)	-0.9	-18.61	3.14E-23	8.15E-20
SPBC1709.12	rid1	GTPase binding protein Rid1 (predicted)	-0.43	-7.82	5.03E-10	4.36E-07
SPBC1271.12	kes1	sterol transfer protein Kes1 (predicted)	-0.49	-7.63	9.52E-10	6.19E-07
SPBC336.03	efc25	Ras1 GEF Efc25	-0.38	-6.16	1.56E-07	5.07E-05
SPBC1685.13	fhn1	eisosome assembly protein Fhn1	-0.46	-5.81	5.25E-07	1.36E-04
SPBC12D12.05c	SPBC12D12.05c	mitochondrial carrier, ATP:ADP antiporter (predicted)	-0.32	-5.43	1.95E-06	3.63E-04
SPAC11G7.01	mtl2	plasma membrane-associated serine-rich cell wall sensor Mtl2	-0.34	-5.33	2.76E-06	4.61E-04
SPBC1734.05c	spf31	DNAJ protein, splicing factor Spf31 (predicted)	-0.4	-5.32	2.83E-06	4.61E-04
SPCC576.13	swc5	Swr1 complex subunit Swc5	-0.45	-5.23	3.87E-06	5.59E-04
SPBC11B10.07c	ivn1	plasma membrane phospholipid-translocating ATPase complex Lem3 family subunit Ivn1 (predicted)	-0.29	-4.95	1.02E-05	1.40E-03
SPBC25H2.16c	gga22	Golgi localized Arf binding gamma-adaptin ortholog Gga22	-0.25	-4.78	1.78E-05	1.78E-03
SPBC3F6.01c	SPBC3F6.01c	TPR repeat serine/threonine protein phosphatase (predicted)	-0.32	-4.78	1.76E-05	1.78E-03
SPAC2C4.07c	dis32	3'-5'-exoribonuclease activity Dis3L2	-0.25	-4.56	3.76E-05	3.05E-03
SPBC2D10.06	rep1	MBF transcription factor activator Rep1	-0.3	-4.5	4.48E-05	3.43E-03
SPCC126.15c	sec65	signal recognition particle subunit Sec65 (predicted)	-0.44	-4.46	5.09E-05	3.67E-03
SPAC3A11.08	pcu4	cullin 4	-0.47	-4.36	7.12E-05	4.87E-03
SPBC13A2.02	nup82	nucleoporin, WD repeat Nup82	-0.34	-4.29	8.87E-05	5.77E-03
SPBC651.03c	gyp10	GTPase activating protein Gyp10	-0.24	-4.18	1.28E-04	7.22E-03
SPAC4C5.04	rad31	SUMO activating enzyme E1-type Rad31	-0.39	-4.15	1.37E-04	7.60E-03
SPBC337.09	erg28	Erg28 protein (predicted)	-0.22	-4.16	1.52E-04	8.08E-03
SPAC458.05	pik3	phosphatidylinositol 3-kinase Pik3	-0.44	-4.01	2.19E-04	1.04E-02
SPBC29A3.07c	sap14	U2 snRNP-associated protein SF3B14 Sap14	-0.3	-3.84	3.68E-04	1.59E-02
SPAC3G6.04	rnp24	RNA-binding protein Rnp24	-0.39	-3.82	3.94E-04	1.65E-02
SPBC216.06c	swi1	replication fork protection complex subunit Swi1	-0.34	-3.79	4.33E-04	1.68E-02
SPBC21C3.11	ubx4	UBX domain protein Ubx4 (predicted)	-0.33	-3.83	4.21E-04	1.68E-02
SPAC23G3.08c	ubp7	ubiquitin C-terminal hydrolase Ubp7	-0.22	-3.77	4.97E-04	1.85E-02
SPBC1347.06c	cki1	serine/threonine protein kinase Cki1	-0.21	-3.73	5.14E-04	1.88E-02
SPBC13E7.08c	leo1	RNA polymerase II associated Paf1 complex subunit Leo1	-0.2	-3.7	5.70E-04	2.03E-02
SPBC26H8.08c	grn1	GTPase Grn1	-0.4	-3.67	6.20E-04	2.10E-02
SPCC4F11.03c	SPCC4F11.03c	Schizosaccharomyces specific protein	-0.19	-3.67	6.29E-04	2.10E-02
SPAC25B8.06c	dia4	mitochondrial serine-tRNA ligase (predicted)	-0.39	-3.65	6.52E-04	2.12E-02
SPAC22G7.05	kri1	ribosome biogenesis protein Kri1 (predicted)	-0.42	-3.64	6.76E-04	2.17E-02
SPBC16E9.09c	erp5	COPII vesicle coat component Erp5/Erp6 (predicted)	-0.24	-3.58	8.06E-04	2.48E-02
SPCC1494.08c	SPCC1494.08c	cortical variant C2 domain protein, human FAM102A and FAM102B ortholog, implicated in signalling or endocytosis	-0.17	-3.57	8.51E-04	2.57E-02
SPAC630.07c	SPAC630.07c	Schizosaccharomyces specific protein	-0.24	-3.56	8.77E-04	2.62E-02
SPCP1E11.05c	are2	acyl-coA-sterol acyltransferase Are2	-0.18	-3.53	9.55E-04	2.73E-02
SPBC405.02c	SPBC405.02c	Schizosaccharomyces specific protein	-0.25	-3.4	1.40E-03	3.59E-02
SPAC15A10.06	SPAC15A10.06	CPA1 sodium ion/proton antiporter (predicted)	-0.31	-3.41	1.42E-03	3.62E-02
SPAC26F1.10c	pyp1	tyrosine phosphatase Pyp1	-0.35	-3.38	1.58E-03	3.83E-02
SPAC11D3.07c	toe4	transcription factor, zf-fungal binuclear cluster type(predicted)	-0.21	-3.36	1.67E-03	3.96E-02
SPBP8B7.05c	nce103	carbonic anhydrase (predicted)	-0.24	-3.32	1.77E-03	4.14E-02
SPAC4F10.15c	wsp1	WASp homolog	-0.33	-3.29	1.92E-03	4.41E-02

SPBC4F6.16c	ero11	ER oxidoreductin Ero1a	-0.32	-3.28	1.95E-03	4.45E-02
SPAC1486.08	cox16	mitochondrial copper chaperone for cytochrome c oxidase Cox16 (predicted)	-0.23	-3.26	2.06E-03	4.65E-02
SPAC23A1.19c	hrq1	RecQ type DNA helicase Hrq1 (predicted)	-0.28	-3.24	2.18E-03	4.77E-02
SPCC1620.04c	fzr3	meiotic fizzy-related APC coactivator Fzr3	-0.18	-3.24	2.18E-03	4.77E-02
SPBC11C11.02	imp2	F-BAR domain protein Imp2	-0.26	-3.24	2.24E-03	4.81E-02

Supplementary table 2: Positive genetic interactions for *btn1D363G* vs *ade6*Δ control

Systematic ID	Gene name	Product description	Colony Size Difference	t	P Value	Adjusted P Value
SPAPB2B4.02	grx5	mitochondrial [2Fe-2S] cluster assembly and transfer glutaredoxin Grx5	0.38	9.28	8.44E-12	1.10E-08
SPAC17G6.04c	cpp1	protein farnesyltransferase beta subunit Cpp1	0.41	7.39	2.17E-09	1.13E-06
SPBC1604.03c	SPBC1604.03c	conserved fungal protein, implicated in vesicle trafficking or lipid metabolism	0.34	6.91	1.14E-08	4.93E-06
SPAC23A1.03	apt1	adenine phosphoribosyltransferase (APRT) Apt1	0.38	6.64	4.39E-08	1.63E-05
SPBC1A4.05	blt1	ubiquitin domain-like protein Blt1	0.47	5.84	4.73E-07	1.36E-04
SPAC1486.04c	alm1	nucleoporin Alm1	0.26	5.63	1.30E-06	2.83E-04
SPBC1734.12c	alg12	dolichyl pyrophosphate Man7GlcNAc2 alpha-1,6-mannosyltransferase Alg12 (predicted)	0.29	5.55	1.29E-06	2.83E-04
SPCC320.06	SPCC320.06	conserved fungal protein	0.31	5.46	1.79E-06	3.58E-04
SPCC1393.08	fil1	transcription factor, zf-GATA type	0.35	5.3	3.10E-06	4.75E-04
SPBC577.02	rpl3801	60S ribosomal protein L38 (predicted)	0.28	4.97	1.15E-05	1.49E-03
SPCPB1C11.01	amt1	plasma membrane ammonium transmembrane transporter Amt1	0.35	4.9	1.20E-05	1.49E-03
SPBC19F8.08	rps401	40S ribosomal protein S4 (predicted)	0.3	4.87	1.31E-05	1.52E-03
SPCC18B5.10c	tex1	TREX complex subunit Tex1 (predicted)	0.45	4.86	1.35E-05	1.52E-03
SPCC613.03	SPCC613.03	endoplasmic reticulum EF hand protein (predicted)	0.34	4.79	1.72E-05	1.78E-03
SPAC3G9.03	rpl2301	60S ribosomal protein L23	0.28	4.75	1.96E-05	1.85E-03
SPAC6G9.12	cfr1	exomer complex BRCT domain subunit Cfr1	0.26	4.75	1.99E-05	1.85E-03
SPCC1235.03	cue2	no go decay endonuclease Cue2	0.28	4.72	2.16E-05	1.93E-03
SPAC1834.05	alg9	mannosyltransferase complex subunit Alg9 (predicted)	0.24	4.74	2.39E-05	2.07E-03
SPBP16F5.05c	yar1	ribosome biogenesis protein Yar1 (predicted)	0.32	4.56	3.75E-05	3.05E-03
SPBC1734.13	atp3	F1-FO ATP synthase gamma subunit (predicted)	0.23	4.52	4.26E-05	3.36E-03
SPCC794.15	SPCC794.15	Schizosaccharomyces specific protein	0.3	4.52	4.85E-05	3.60E-03
SPBC1703.13c	SPBC1703.13c	mitochondrial carrier, inorganic phosphate (predicted)	0.17	4.4	6.32E-05	4.44E-03
SPBC21B10.10	rps402	40S ribosomal protein S4 (predicted)	0.29	4.29	8.87E-05	5.77E-03
SPAC644.15	rpp101	60S acidic ribosomal protein A1	0.22	4.26	9.77E-05	6.16E-03
SPBC1711.03	emc3	ER membrane protein complex subunit Emc3 (predicted)	0.65	4.26	9.95E-05	6.16E-03
SPAC16.03c	ura2	dihydroorotase Ura2	0.27	4.22	1.13E-04	6.84E-03
SPBC1778.06c	fim1	fimbrin	0.26	4.2	1.18E-04	6.89E-03
SPCC1919.10c	myo52	myosin type V	0.34	4.2	1.19E-04	6.89E-03
SPCC330.14c	rpl2402	60S ribosomal protein L24 (predicted)	0.28	4.12	1.52E-04	8.08E-03
SPCC1223.05c	rpl3702	60S ribosomal protein L37 (predicted)	0.3	4.11	1.58E-04	8.19E-03
SPAC323.05c	mtq2	eRF1 methyltransferase Mtq2 (predicted)	0.2	4.08	1.75E-04	8.71E-03
SPBC16H5.12c	SPBC16H5.12c	DUF2433 metallo phosphatase superfamily conserved fungal protein	0.36	4.07	1.78E-04	8.71E-03
SPCC1235.02	bio2	biotin synthase	0.26	4.08	1.76E-04	8.71E-03
SPAC144.11	rps1102	40S ribosomal protein S11 (predicted)	0.23	4.02	2.08E-04	1.00E-02
SPBC1921.03c	mex67	mRNA export receptor, Tap, nucleoporin Mex67	0.18	3.99	2.34E-04	1.09E-02
SPAC13C5.05c	SPAC13C5.05c	N-acetylglucosamine-phosphate mutase (predicted)	0.21	3.96	2.56E-04	1.17E-02
SPBC1711.05	srp40	nucleocytoplasmic transport chaperone Srp40 (predicted)	0.38	3.95	2.62E-04	1.17E-02
SPCP20C8.02c	SPCP20C8.02c	S. pombe specific UPF0321 family protein 1	0.26	3.85	3.55E-04	1.56E-02
SPAC19D5.11c	ctf8	Ctf18 RFC-like complex subunit Ctf8	0.24	3.86	3.75E-04	1.60E-02
SPAC637.06	gmh5	alpha-1,2-galactosyltransferase (predicted)	0.22	3.81	4.03E-04	1.66E-02
SPBC839.13c	rpl1601	60S ribosomal protein L13/L16 (predicted)	0.22	3.79	4.33E-04	1.68E-02
SPCC548.07c	ght1	plasma membrane high-affinity glucose:proton symporter Ght1	0.22	3.78	4.40E-04	1.68E-02

SPCC569.02c	SPCC569.02c	S. pombe specific UPF0321 family protein 2	0.21	3.78	4.39E-04	1.68E-02
SPBC9B6.03	SPBC9B6.03	zf-FYVE type zinc finger protein, involved in endosomal transport	0.3	3.78	4.47E-04	1.68E-02
SPBC19C2.13c	ctu2	cytosolic thiouridylase subunit Ctu2	0.2	3.71	5.52E-04	1.99E-02
SPAC1B3.15c	SPAC1B3.15c	transmembrane transporter (predicted)	0.19	3.67	6.29E-04	2.10E-02
SPBC685.06	rps001	40S ribosomal protein S0A (p40)	0.23	3.67	6.22E-04	2.10E-02
SPCC320.07c	mde7	RNA-binding protein Mde7	0.22	3.68	6.09E-04	2.10E-02
SPCC553.01c	dbl2	DNA recombination protein Dbl2	0.2	3.66	6.38E-04	2.10E-02
SPBC18H10.13	rps1402	40S ribosomal protein S14 (predicted)	0.19	3.64	6.84E-04	2.17E-02
SPCC757.07c	ctt1	catalase	0.23	3.62	7.14E-04	2.24E-02
SPBC839.05c	rps1701	40S ribosomal protein S17 (predicted)	0.19	3.58	8.11E-04	2.48E-02
SPAC144.04c	spe1	ornithine decarboxylase Spe1 (predicted)	0.21	3.55	8.91E-04	2.62E-02
SPAC19B12.11c	bud20	zinc finger ribosome biogenesis protein Bud20 (predicted)	0.2	3.57	8.97E-04	2.62E-02
SPAC30D11.05	aps3	AP-3 adaptor complex subunit Aps3 (predicted)	0.18	3.54	9.08E-04	2.62E-02
SPBP35G2.08c	air1	TRAMP complex zinc knuckle subunit Air1	0.2	3.51	9.91E-04	2.80E-02
SPAC6F6.12	atg24	autophagy associated PX/BAR domain sorting nexin Atg24	0.32	3.5	1.03E-03	2.87E-02
SPAC8E11.07c	alp31	tubulin specific chaperone cofactor A, Alp31	0.19	3.52	1.05E-03	2.87E-02
SPBC776.11	rpl2801	60S ribosomal protein L27/L28	0.21	3.5	1.04E-03	2.87E-02
SPBC725.15	ura5	orotate phosphoribosyltransferase Ura5	0.23	3.49	1.08E-03	2.93E-02
SPBC1921.01c	rpl35b	60S ribosomal protein L35a (predicted)	0.2	3.47	1.12E-03	3.01E-02
SPAC11G7.03	idh1	isocitrate dehydrogenase (NAD+) subunit 1 Idh1	0.2	3.4	1.39E-03	3.59E-02
SPBC3D6.04c	mad1	mitotic spindle checkpoint protein Mad1	0.24	3.4	1.38E-03	3.59E-02
SPCC1223.01	SPCC1223.01	ubiquitin-protein ligase E3 involved in rescue of stalled ribosomes (predicted)	0.22	3.4	1.38E-03	3.59E-02
SPCC970.05	rpl3601	60S ribosomal protein L36	0.15	3.38	1.47E-03	3.70E-02
SPAC14C4.14	atp1	F1-FO ATP synthase alpha subunit	0.35	3.37	1.51E-03	3.77E-02
SPAC26H5.05	mga2	IPT/TIG ankyrin repeat gene-specific transcription coactivator Mga2	0.36	3.37	1.53E-03	3.78E-02
SPCC330.19c	SPCC330.19c	Schizosaccharomyces pombe specific protein	0.21	3.36	1.56E-03	3.82E-02
SPCC594.04c	SPCC594.04c	steroid oxidoreductase superfamily protein (predicted)	0.27	3.34	1.65E-03	3.96E-02
SPCC613.12c	raf1	CLRC ubiquitin ligase complex WD repeat subunit Raf1/Dos1	0.4	3.34	1.67E-03	3.96E-02
SPCC285.15c	rps2802	40S ribosomal protein S28, Rps2802	0.24	3.31	1.78E-03	4.14E-02
SPCC1739.14	npp106	nucleoporin Npp106	0.2	3.26	2.09E-03	4.68E-02
SPBC1711.15c	SPBC1711.15c	Schizosaccharomyces pombe specific protein	0.27	3.26	2.18E-03	4.77E-02
SPAC29A4.18	prw1	Clr6 histone deacetylase complex subunit Prw1	0.21	3.23	2.24E-03	4.81E-02
SPBC15D4.09c	met3	cystathionine gamma-synthase Met3	0.42	3.22	2.32E-03	4.95E-02
