# Supplementary material for: Global network analysis in Schizosaccharomyces pombe reveals three distinct consequences of the common 1-kb deletion causing juvenile CLN3 disease
Source: Sci Rep. 2021 Mar 18;11:6332. doi: 10.1038/s41598-021-85471-4 (PMC7973434; doi:10.1038/s41598-021-85471-4)
Supplement: Supplementary file 4 — S4: Supplementary Tables 4. [file 41598_2021_85471_MOESM4_ESM.pdf]

Global network analysis in *Schizosaccharomyces pombe* reveals three distinct consequences of the common 1-kb deletion causing juvenile CLN3 disease

Christopher J. Minnis[1,2], StJohn Townsend[3,4], Julia Petschnigg[1], Elisa Tinelli[1], Jürg Bähler[3], Claire Russell[2], Sara E. Mole[1]

[1]MRC Laboratory for Molecular Cell Biology and Great Ormond Street Institute of Child Health, University College London, London WC1E 6BT, UK
[2]Dept. Comparative Biomedical Sciences, Royal Veterinary College, Royal College Street, London NW1 0TU, UK
[3]Institute of Healthy Ageing, Department of Genetics, Evolution and Environment, University College London, London WC1E 6BT, UK
[4]The Molecular Biology of Metabolism Laboratory, The Francis Crick Institute, London, NW1 1AT, United Kingdom

*Corresponding author: christopher.minnis.15@ucl.ac.uk

Supplementary table 1: Negative genetic interactions *btn1(102-208del)* vs *ade6Δ* control

Systematic ID	Gene name	Product description	Colony Size Difference	t	P Value	Adjusted P Value
SPBC2F12.15c	pfa3	palmitoyltransferase Pfa3 (predicted)	-0.9	-13.37	1.43E-17	3.72E-14
SPBP8B7.05c	nce103	carbonic anhydrase (predicted)	-0.86	-8.39	7.17E-11	9.32E-08
SPBC25H2.16c	gga22	Golgi localized Arf binding gamma-adaptin ortholog Gga22	-0.55	-7.61	1.03E-09	8.69E-07
SPBC1D7.03	clg1	cyclin-like protein involved in autophagy Clg1 (predicted)	-0.47	-6.84	1.50E-08	7.81E-06
SPBC1271.12	kes1	sterol transfer protein Kes1 (predicted)	-0.58	-6.66	2.79E-08	1.21E-05
SPBC23E6.01c	cxr1	splicing factor Cxr1	-0.53	-6.04	2.36E-07	7.66E-05
SPBC543.07	pek1	MAP kinase kinase Pek1	-0.48	-5.88	4.16E-07	9.01E-05
SPCP1E11.05c	are2	acyl-coA-sterol acyltransferase Are2	-0.41	-5.95	3.32E-07	9.01E-05
SPAC4H3.13	pcc1	EKC/KEOPS complex subunit Pcc1 (predicted)	-0.88	-5.7	7.89E-07	1.37E-04
SPAC13G6.15c	SPAC13G6.15c	calcipressin (predicted)	-0.6	-5.66	8.95E-07	1.45E-04
SPAC4G8.10	gos1	SNARE Gos1 (predicted)	-0.47	-5.61	1.07E-06	1.64E-04
SPBC1685.13	fhn1	eisosome assembly protein Fhn1	-0.59	-5.45	1.82E-06	2.43E-04
SPBC887.05c	cwf29	RNA-binding protein Cwf29	-0.66	-5.45	1.87E-06	2.43E-04
SPBC530.08	SPBC530.08	membrane-tethered transcription factor (predicted)	-0.41	-5.41	2.09E-06	2.54E-04
SPBC887.15c	sur2	sphingosine hydroxylase/sphingolipid delta-4 desaturase activity Sur2	-0.44	-5.36	2.49E-06	2.77E-04
SPCC4F11.03c	SPCC4F11.03c	Schizosaccharomyces specific protein	-0.36	-5.25	3.61E-06	3.61E-04
SPAC25B8.06c	dia4	mitochondrial serine-tRNA ligase (predicted)	-0.78	-5.22	4.04E-06	3.75E-04
SPAC823.10c	hem25	mitochondrial carrier, glycine Hem25 (predicted)	-0.55	-5.23	3.95E-06	3.75E-04
SPBC26H8.08c	grn1	GTPase Grn1	-0.8	-5.19	4.47E-06	4.00E-04
SPAC458.05	pik3	phosphatidylinositol 3-kinase Pik3	-0.79	-5.18	4.64E-06	4.02E-04
SPBC1778.03c	SPBC1778.03c	NADH pyrophosphatase (predicted)	-0.43	-5.11	5.80E-06	4.68E-04
SPCC16C4.10	SPCC16C4.10	6-phosphogluconolactonase (predicted)	-0.32	-5.1	6.16E-06	4.71E-04
SPBC4B4.03	rsc1	RSC complex subunit Rsc1	-0.52	-5.05	7.09E-06	5.21E-04
SPAC1486.08	cox16	mitochondrial copper chaperone for cytochrome c oxidase Cox16 (predicted)	-0.47	-4.98	9.10E-06	6.39E-04
SPCC1494.08c	SPCC1494.08c	cortical variant C2 domain protein, human FAM102A and FAM102B ortholog, implicated in signalling or endocytosis	-0.33	-4.97	9.42E-06	6.45E-04
SPAC3A11.08	pcu4	cullin 4	-0.73	-4.96	9.78E-06	6.52E-04
SPAC30C2.06c	dml1	mitochondrial inheritance GTPase, tubulin-like (predicted)	-0.63	-4.92	1.13E-05	6.93E-04
SPAC23A1.19c	hrq1	RecQ type DNA helicase Hrq1 (predicted)	-0.58	-4.86	1.38E-05	7.82E-04
SPAC11G7.01	mtl2	plasma membrane-associated serine-rich cell wall sensor Mtl2	-0.4	-4.78	1.78E-05	9.06E-04
SPAC4C5.04	rad31	SUMO activating enzyme E1-type Rad31	-0.62	-4.78	1.77E-05	9.06E-04
SPBC646.13	sds23	PP2A-type phosphatase inhibitor Sds23/Moc1	-0.62	-4.8	1.66E-05	9.06E-04
SPBC2D10.13	est1	telomerase regulator Est1	-0.41	-4.74	2.02E-05	1.01E-03
SPAC4F10.15c	wsp1	WASp homolog	-0.64	-4.67	2.58E-05	1.20E-03
SPCC790.02	pep3	HOPS/CORVET complex subunit, ubiquitin-protein ligase E3 Pep3/Vps18 (predicted)	-0.64	-4.65	2.74E-05	1.25E-03
SPBC6B1.04	mde4	microtubule-site clamp monopolin complex subunit Mde4	-0.35	-4.58	3.51E-05	1.50E-03
SPBC17A3.09c	aim22	lipoate-protein ligase A (predicted)	-0.47	-4.51	4.41E-05	1.79E-03
SPAC19G12.13c	poz1	shelterin complex subunit Poz1	-0.54	-4.47	4.94E-05	1.92E-03
SPCC126.15c	sec65	signal recognition particle subunit Sec65 (predicted)	-0.6	-4.48	4.85E-05	1.92E-03
SPBC2A9.06c	nus1	di-trans,poly-cis-decaprenylcistransferase Nus1	-0.73	-4.47	5.02E-05	1.92E-03
SPBC1604.02c	ppr1	mitochondrial PPR repeat protein Ppr1	-0.38	-4.45	5.33E-05	2.01E-03
SPBC28F2.10c	ngg1	SAGA complex subunit Ngg1/Ada3	-0.38	-4.43	5.65E-05	2.10E-03
SPAC3H8.09c	nab3	poly(A) binding protein Nab3 (predicted)	-0.4	-4.4	6.27E-05	2.19E-03

SPBC409.07c	wis1	MAP kinase kinase Wis1	-0.84	-4.38	6.57E-05	2.25E-03
SPAC144.03	ade2	adenylosuccinate synthetase Ade2	-0.47	-4.38	6.68E-05	2.26E-03
SPAC22G7.05	kri1	ribosome biogenesis protein Kri1 (predicted)	-0.7	-4.36	7.16E-05	2.33E-03
SPAC4C5.02c	ryh1	GTPase Ryh1	-0.51	-4.35	7.27E-05	2.33E-03
SPBC4B4.07c	usp102	U1 snRNP-associated protein Usp102	-0.42	-4.36	7.19E-05	2.33E-03
SPCC594.05c	spf1	Set1C PHD finger subunit Spf1	-0.33	-4.35	7.27E-05	2.33E-03
SPAC26H5.10c	tif51	translation elongation and termination factor eIF5A (predicted)	-0.39	-4.33	7.85E-05	2.49E-03
SPBC1105.10	rav1	RAVE complex subunit Rav1	-0.3	-4.31	8.43E-05	2.61E-03
SPBC11G11.01	fis1	mitochondrial fission protein Fis1 (predicted)	-0.38	-4.31	8.35E-05	2.61E-03
SPBC31F10.12	tma20	RNA-binding protein Tma20 (predicted)	-0.37	-4.3	8.52E-05	2.61E-03
SPBC3H7.15	hhp1	serine/threonine protein kinase Hhp1	-0.28	-4.28	9.16E-05	2.77E-03
SPBC25H2.08c	mrs2	mitochondrial inner membrane magnesium ion transmembrane transporter Mrs2 (predicted)	-0.29	-4.26	9.70E-05	2.83E-03
SPBC428.08c	clr4	histone lysine H3 methyltransferase Clr4	-0.42	-4.25	1.02E-04	2.94E-03
SPBC11C11.02	imp2	F-BAR domain protein Imp2	-0.47	-4.21	1.16E-04	3.25E-03
SPCC1739.10	mug33	Tea1-interacting protein involved in exocytosis	-0.47	-4.21	1.15E-04	3.25E-03
SPAC2F3.15	lsk1	P-TEFb-associated cyclin-dependent protein kinase Lsk1	-0.75	-4.23	1.19E-04	3.26E-03
SPAC2F7.17	mrf1	mitochondrial translation release factor (predicted)	-0.27	-4.18	1.27E-04	3.37E-03
SPBC543.09	yta12	mitochondrial m-AAA protease Yta12 (predicted)	-0.41	-4.18	1.27E-04	3.37E-03
SPBC557.02c	SPBC557.02c	DUF2458 conserved fungal protein	-0.32	-4.18	1.27E-04	3.37E-03
SPAC19B12.10	sst2	human AMSH/STAMBP protein homolog, ubiquitin specific-protease	-0.57	-4.2	1.35E-04	3.54E-03
SPBC30D10.10c	tor1	serine/threonine protein kinase Tor1	-0.65	-4.15	1.39E-04	3.63E-03
SPBC354.03	swd3	Set1C complex WD repeat protein Swd3	-0.42	-4.14	1.44E-04	3.71E-03
SPBC12C2.04	SPBC12C2.04	NAD binding dehydrogenase family protein	-0.32	-4.13	1.48E-04	3.72E-03
SPBC3F6.01c	SPBC3F6.01c	TPR repeat serine/threonine protein phosphatase (predicted)	-0.35	-4.13	1.51E-04	3.74E-03
SPBC337.09	erg28	Erg28 protein (predicted)	-0.37	-4.15	1.54E-04	3.76E-03
SPBC26H8.14c	cox17	mitochondrial copper chaperone for cytochrome c oxidase Cox17 (predicted)	-0.46	-4.1	1.63E-04	3.92E-03
SPAC222.05c	mss1	mitochondrial tRNA wobble uridine modification GTPase Mss1 (predicted)	-0.27	-4.09	1.71E-04	3.98E-03
SPAC5D6.05	med18	mediator complex subunit Med18	-0.76	-4.09	1.69E-04	3.98E-03
SPBC2D10.06	rep1	MBF transcription factor activator Rep1	-0.35	-4.06	1.83E-04	4.22E-03
SPBC3H7.03c	kgd1	2-oxoglutarate dehydrogenase (lipoamide) (e1 component of oxoglutarate dehydrogenase complex) (predicted)	-0.41	-4.04	2.00E-04	4.57E-03
SPBC1734.05c	spf31	DNAJ protein, splicing factor Spf31 (predicted)	-0.39	-3.96	2.52E-04	5.60E-03
SPCC1494.10	adn3	transcription factor Adn3	-0.33	-3.96	2.52E-04	5.60E-03
SPAC16C9.05	cph1	Clr6 histone deacetylase associated PHD finger protein Cph1	-0.56	-3.98	2.62E-04	5.76E-03
SPBC646.09c	int6	translation initiation factor eIF3e	-0.82	-3.93	2.79E-04	6.00E-03
SPBC11B10.07c	ivn1	plasma membrane phospholipid-translocating ATPase complex Lem3 family subunit Ivn1 (predicted)	-0.3	-3.9	3.09E-04	6.59E-03
SPBC23E6.08	sat1	Golgi membrane exchange factor subunit Sat1 (predicted)	-0.56	-3.89	3.20E-04	6.77E-03
SPBC29A10.08	gas2	cell wall 1,3-beta-glucanosyltransferase Gas2	-0.31	-3.87	3.33E-04	6.93E-03
SPAC6G9.15c	ebp1	Ell binding protein Ebp1	-0.38	-3.85	3.58E-04	7.38E-03
SPBC27B12.03c	erg32	C-5 sterol desaturase Erg32	-0.42	-3.84	3.65E-04	7.48E-03
SPBPB2B2.13	gal1	galactokinase Gal1	-0.34	-3.83	3.81E-04	7.73E-03
SPBC887.10	mcs4	response regulator Mcs4	-0.6	-3.83	3.85E-04	7.76E-03
SPCC576.13	swc5	Swr1 complex subunit Swc5	-0.43	-3.8	4.24E-04	8.48E-03
SPAC13G6.14	aps1	diadenosine 5',5'''-p1,p6-hexaphosphate hydrolase Aps1	-0.71	-3.76	4.66E-04	8.97E-03
SPBC4F6.06	kin1	microtubule affinity-regulating kinase Kin1	-0.36	-3.76	4.73E-04	9.05E-03

SPBC776.02c	dis2	serine/threonine protein phosphatase PP1, Dis2	-0.24	-3.73	5.20E-04	9.87E-03
SPBC25D12.06	SPBC25D12.06	mitochondrial ATP-dependent RNA helicase (predicted)	-0.45	-3.7	5.62E-04	1.04E-02
SPBC32F12.01c	css1	inositol phosphosphingolipid phospholipase C, Css1	-0.4	-3.69	5.80E-04	1.06E-02
SPBC947.08c	hip4	histone promoter control protein Hip4	-0.43	-3.67	6.13E-04	1.11E-02
SPBC1A4.04	SPBC1A4.04	Schizosaccharomyces specific protein	-0.33	-3.67	6.27E-04	1.13E-02
SPBC12D12.06	srb11	mediator complex cyclin subunit Srb11	-0.46	-3.66	6.35E-04	1.14E-02
SPCC794.11c	ent3	ENTH/VHS domain protein Ent3 (predicted)	-0.32	-3.66	6.49E-04	1.16E-02
SPBC1289.08	uap1	UDP-N-acetylglucosamine diphosphorylase Uap1/Qri1(predicted)	-0.75	-3.62	7.13E-04	1.23E-02
SPBC21B10.03c	ath1	ataxin-2 homolog	-0.38	-3.62	7.17E-04	1.23E-02
SPAC31G5.04	lys12	homoisocitrate dehydrogenase Lys12	-0.25	-3.61	7.47E-04	1.27E-02
SPCC18.17c	SPCC18.17c	proteasome assembly chaperone (predicted)	-0.28	-3.61	7.55E-04	1.27E-02
SPBC1703.12	ubp9	ubiquitin C-terminal hydrolase Ubp9	-0.55	-3.59	7.80E-04	1.30E-02
SPAC26F1.10c	pyp1	tyrosine phosphatase Pyp1	-0.65	-3.61	7.98E-04	1.31E-02
SPBC3E7.01	fab1	1-phosphatidylinositol-3-phosphate 5-kinase Fab1	-0.42	-3.59	8.00E-04	1.31E-02
SPCC285.13c	nup60	nucleoporin Nup60	-0.4	-3.59	7.98E-04	1.31E-02
SPAC1556.08c	cbs2	AMP-activated protein kinase gamma subunit Cbs2	-0.29	-3.58	8.24E-04	1.33E-02
SPBC839.15c	tef103	translation elongation factor EF-1 alpha Ef1a-c	-0.48	-3.56	8.62E-04	1.37E-02
SPAC1A6.04c	plb1	phospholipase B homolog Plb1	-0.46	-3.55	8.89E-04	1.38E-02
SPAC8F11.02c	dph3	diphthamide biosynthesis protein Dph3 (predicted)	-0.49	-3.55	8.85E-04	1.38E-02
SPBC21C3.07c	trm140	tRNA (cytosine-3) methyltransferase Trm140 (predicted)	-0.63	-3.54	9.18E-04	1.40E-02
SPBC336.03	efc25	Ras1 GEF Efc25	-0.29	-3.52	9.86E-04	1.48E-02
SPCC1919.03c	amk2	AMP-activated protein kinase beta subunit Amk2	-0.3	-3.51	9.95E-04	1.48E-02
SPBC530.06c	clu1	clustered mitochndria ortholog Clu1	-0.43	-3.51	1.00E-03	1.48E-02
SPBC2D10.16	mhf1	CENP-S ortholog, FANCM-MHF complex subunit Mhf1	-0.47	-3.52	1.05E-03	1.53E-02
SPAC15A10.06	SPAC15A10.06	CPA1 sodium ion/proton antiporter (predicted)	-0.54	-3.5	1.10E-03	1.57E-02
SPAC1D4.01	tls1	splicing factor Tls1	-0.26	-3.48	1.09E-03	1.57E-02
SPAC1F7.09c	dal2	allantoicase Dal2	-0.41	-3.41	1.34E-03	1.84E-02
SPAC1687.23c	SPAC1687.23c	Schizosaccharomyces pombe specific protein	-0.33	-3.4	1.48E-03	2.02E-02
SPAC22E12.19	snt1	Set3 complex subunit Snt1	-0.41	-3.36	1.56E-03	2.07E-02
SPBC21C3.20c	git1	C2 domain protein Git1	-0.21	-3.36	1.58E-03	2.09E-02
SPAC23G3.03	sib2	ornithine N5 monooxygenase (predicted)	-0.26	-3.34	1.65E-03	2.14E-02
SPBC16E9.09c	erp5	COPII vesicle coat component Erp5/Erp6 (predicted)	-0.3	-3.33	1.71E-03	2.20E-02
SPBC106.16	pre6	20S proteasome complex subunit alpha 4 Pre6	-0.54	-3.31	1.78E-03	2.27E-02
SPAC5H10.04	SPAC5H10.04	NADPH dehydrogenase, (Old yellow enzyme) involved in small alpha,beta-unsaturated carbonyl compounds (predicted)	-0.33	-3.31	1.81E-03	2.29E-02
SPBC577.11	SPBC577.11	DUF3074 family protein, implicated in vesicle trafficking or lipid metabolism	-0.37	-3.3	1.88E-03	2.37E-02
SPAC630.07c	SPAC630.07c	Schizosaccharomyces specific protein	-0.3	-3.29	1.89E-03	2.37E-02
SPAC57A7.04c	pabp	major poly(A) binding protein Pabp/Pab1	-0.53	-3.29	1.91E-03	2.39E-02
SPBC119.06	sco1	mitochondrial copper chaperone for cytochrome c oxidase Sco1 (predicted)	-0.28	-3.29	1.93E-03	2.40E-02
SPAC694.04c	SPAC694.04c	nuclear/mitochondrial metal-dependent protein hydrolase, human MYG1 ortholog	-0.25	-3.27	2.04E-03	2.48E-02
SPBC13E7.08c	leo1	RNA polymerase II associated Paf1 complex subunit Leo1	-0.23	-3.27	2.05E-03	2.48E-02
SPBC17D1.05	SPBC17D1.05	Schizosaccharomyces specific protein	-0.33	-3.27	2.05E-03	2.48E-02
SPAC11E3.05	sea3	SEA complex ubiquitin-protein ligase E3 subunit Sea3 (predicted)	-0.28	-3.25	2.15E-03	2.57E-02
SPBC409.11	meu18	Schizosaccharomyces specific protein Meu18	-0.26	-3.25	2.14E-03	2.57E-02
SPCC1919.05	ski3	Ski complex TPR repeat subunit Ski3 (predicted)	-0.23	-3.24	2.20E-03	2.61E-02

SPAC212.03	SPAC212.03	hypothetical protein		-0.47	-3.26	2.21E-03	2.61E-02
SPBC947.15c	ndi1	internal mitochondrial NADH dehydrogenase (ubiquinone) Ndi1 (predicted)		-0.26	-3.22	2.32E-03	2.70E-02
SPAC6F12.02	rst2	transcription factor Rst2		-0.4	-3.21	2.38E-03	2.73E-02
SPBC1685.08	cti6	histone deacetylase complex PHD finger subunit Cti6		-0.28	-3.21	2.38E-03	2.73E-02
SPAC1296.06	tah18	CIA machinery NADPH-dependent diflavin oxidoreductase Tah18 (predicted)		-0.75	-3.21	2.41E-03	2.74E-02
SPBC17G9.10	rpl1102	60S ribosomal protein L11 (predicted)		-0.39	-3.2	2.45E-03	2.78E-02
SPAC1039.08	SPAC1039.08	serine acetyltransferase (predicted)		-0.42	-3.2	2.47E-03	2.79E-02
SPAC22H12.05c	fsc1	fasciclin domain protein Fsc1		-0.3	-3.2	2.50E-03	2.81E-02
SPCC548.06c	ght8	plasma membrane hexose:proton symporter, unknown specificity Ght8 (predicted)		-0.3	-3.19	2.58E-03	2.88E-02
SPAC1F7.07c	fip1	plasma membrane iron transmembrane transporter Fip1		-0.77	-3.18	2.61E-03	2.90E-02
SPAC26H5.07c	SPAC26H5.07c	seven transmembrane receptor protein (predicted)		-0.23	-3.15	2.87E-03	3.11E-02
SPAC823.05c	tlg2	SNARE Tlg2 (predicted)		-0.43	-3.12	3.06E-03	3.25E-02
SPAC227.06	yip5	Rab GTPase binding Yip5 (predicted)		-0.28	-3.1	3.28E-03	3.42E-02
SPBC354.12	gpd3	glyceraldehyde 3-phosphate dehydrogenase Gpd3		-0.4	-3.1	3.27E-03	3.42E-02
SPBC83.03c	tas3	RITS complex subunit 3		-0.32	-3.1	3.30E-03	3.42E-02
SPBC56F2.01	pof12	F-box protein Pof12		-0.61	-3.09	3.35E-03	3.44E-02
SPCC794.10	ugp1	UTP-glucose-1-phosphate uridylyltransferase-like Ugp1		-0.23	-3.09	3.36E-03	3.44E-02
SPCPJ732.01	vps5	retromer complex subunit Vps5		-0.57	-3.08	3.45E-03	3.51E-02
SPAC644.06c	cdr1	NIM1 family serine/threonine protein kinase Cdr1/Nim1		-0.63	-3.06	3.62E-03	3.64E-02
SPAC6F12.06	rdi1	Rho GDP dissociation inhibitor Rdi1 (predicted)		-0.2	-3.06	3.63E-03	3.64E-02
SPAP8A3.13c	SPAP8A3.13c	Vid24 family protein (predicted)		-0.24	-3.05	3.75E-03	3.70E-02
SPBC14C8.16c	bot1	mitochondrial ribosomal protein subunit S35		-0.5	-3.05	3.74E-03	3.70E-02
SPAC922.05c	SPAC922.05c	transmembrane transporter (predicted)		-0.28	-3.05	3.78E-03	3.73E-02
SPCC126.04c	sgf73	SAGA complex deubiquitinating submodule subunit Sgf73		-0.3	-3.04	3.84E-03	3.77E-02
SPAC688.13	scn3	TatD DNase family Scn1		-0.23	-3.01	4.15E-03	3.98E-02
SPAC1F3.10c	Oct-01	mitochondrial intermediate peptidase Oct1 (predicted)		-0.63	-3.01	4.17E-03	3.98E-02
SPAC31A2.11c	cuf1	nutritional copper sensing transcription factor Cuf1		-0.22	-3.01	4.23E-03	4.03E-02
SPAC16E8.05c	SPAC16E8.05c	Schizosaccharomyces specific protein Mde1		-0.32	-3	4.35E-03	4.13E-02
SPAC22F8.04	pet1	Golgi phosphoenolpyruvate transmembrane transporter Pet1		-0.33	-3.01	4.41E-03	4.17E-02
SPBC21C3.02c	dep1	Sds3-like family protein Dep1		-0.21	-2.99	4.42E-03	4.17E-02
SPAC2F7.02c	psr1	CTD small phosphatase Psr1 (predicted)		-0.36	-2.99	4.50E-03	4.22E-02
SPAC15E1.06	vps29	retromer complex subunit Vps29		-0.5	-2.99	4.57E-03	4.26E-02
SPAC8E11.05c	SPAC8E11.05c	DUF5102 family conserved fungal protein, associated with clathrin coated vesicles (predicted)		-0.27	-2.98	4.57E-03	4.26E-02
SPBC16E9.16c	lsd90	Lsd90 protein		-0.41	-2.97	4.71E-03	4.34E-02
SPAC513.03	mfm2	M-factor precursor Mfm2		-0.5	-2.97	4.75E-03	4.35E-02
SPAC959.04c	omh6	alpha-1,2-mannosyltransferase Omh6 (predicted)		-0.61	-2.96	4.76E-03	4.35E-02
SPAC688.06c	slx4	structure-specific endonuclease subunit Slx4		-0.28	-2.94	5.13E-03	4.61E-02
SPBCPT2R1.01c	SPBCPT2R1.01c	S. pombe specific DUF999 protein family 9		-0.44	-2.92	5.33E-03	4.75E-02
SPAC3A11.09	sod22	plasma membrane sodium ion/proton antiporter Sod22		-0.18	-2.92	5.43E-03	4.76E-02
SPBC16A3.19	eaf7	histone acetyltransferase complex subunit Eaf7		-0.29	-2.92	5.41E-03	4.76E-02
SPBC3E7.10	fma1	methionine aminopeptidase Fma1 (predicted)		-0.27	-2.92	5.44E-03	4.76E-02
SPBC530.10c	anc1	mitochondrial carrier, ATP:ADP antiporter Anc1		-0.55	-2.92	5.39E-03	4.76E-02
SPCC16C4.09	sts5	cytoplasmic P body 3'-5'-exoribonuclease, Dis3L2-related (predicted)		-0.19	-2.91	5.45E-03	4.76E-02
SPBC13G1.08c	ash2	Ash2-trithorax family protein		-0.2	-2.91	5.54E-03	4.80E-02

SPBC3B8.10c	nem1	Nem1-Spo7 phosphatase complex catalytic subunit Nem1 (predicted)	-0.25	-2.9	5.61E-03	4.85E-02
SPBC1604.08c	imp1	importin alpha family nuclear import signal receptor adaptor Imp1	-0.2	-2.9	5.73E-03	4.89E-02
SPBC1709.04c	cyp3	cyclophilin family peptidyl-prolyl cis-trans isomerase Cyp3	-0.18	-2.9	5.72E-03	4.89E-02
SPCC74.03c	ssp2	AMP-activated protein serine/threonine kinase alpha subunit Ssp2	-0.27	-2.9	5.70E-03	4.89E-02

Supplementary table 2: Positive genetic interactions *btn1(102-208del)* vs *ade6Δ* control

Systematic ID	Gene name	Product description	Colony Size Difference	t	P Value	Adjusted P Value
SPBC29A3.21	SPBC29A3.21	Schizosaccharomyces pombe specific protein	1.19	7.7	1.34E-09	8.69E-07
SPAC17G6.04c	cpp1	protein farnesyltransferase beta subunit Cpp1	0.45	6.35	8.17E-08	3.03E-05
SPBC211.06	gfh1	gamma tubulin complex subunit Gfh1	0.45	5.9	3.85E-07	9.01E-05
SPCC736.09c	tfx1	TRAX	0.42	5.89	3.99E-07	9.01E-05
SPBC16E9.11c	pub3	HECT-type ubiquitin-protein ligase E3 Pub3 (predicted)	0.42	5.73	7.13E-07	1.35E-04
SPBC1921.03c	mex67	mRNA export receptor, Tap, nucleoporin Mex67	0.33	5.72	7.27E-07	1.35E-04
SPBC418.01c	his4	imidazoleglycerol-phosphate synthase His4	0.46	5.49	1.62E-06	2.34E-04
SPAC16E8.14c	tae1	ribosomal protein AdoMet-dependent proline dimethyltransferase Tae1 (predicted)	0.43	5.41	2.15E-06	2.54E-04
SPAC13A11.05	ysp2	peptidase family M17 cytoplasmic leucyl aminopeptidase yspII (LAP yspII)	0.31	5.35	2.56E-06	2.77E-04
SPBC8D2.01	gsk31	serine/threonine protein kinase Gsk31 (predicted)	0.69	5.28	3.25E-06	3.38E-04
SPCC757.11c	SPCC757.11c	transmembrane transporter (predicted)	0.43	5.16	4.98E-06	4.18E-04
SPAC18B11.04	ncs1	neuronal calcium sensor related protein Ncs1	0.36	5.11	5.94E-06	4.68E-04
SPCC320.06	SPCC320.06	conserved fungal protein	0.37	5.05	7.22E-06	5.21E-04
SPCC1235.03	cue2	no go decay endonuclease Cue2	0.37	4.94	1.04E-05	6.77E-04
SPCC569.02c	SPCC569.02c	S. pombe specific UPF0321 family protein 2	0.35	4.93	1.07E-05	6.81E-04
SPCPB1C11.02	SPCPB1C11.02	amino acid transmembrane transporter (predicted)	0.34	4.91	1.15E-05	6.93E-04
SPBC839.11c	hut1	ER uridine diphosphate-glucose transmembrane transporter Hut1	0.37	4.9	1.21E-05	7.16E-04
SPBC9B6.03	SPBC9B6.03	zf-FYVE type zinc finger protein, involved in endosomal transport	0.5	4.86	1.36E-05	7.82E-04
SPAC1952.09c	SPAC1952.09c	acetyl-CoA hydrolase (predicted)	0.35	4.78	1.77E-05	9.06E-04
SPBC354.15	fap1	L-pipecolate oxidase	0.37	4.8	1.68E-05	9.06E-04
SPCC330.11	btb1	BTB/POZ domain protein Btb1	0.45	4.69	2.43E-05	1.19E-03
SPBC1271.09	tgp1	plasma membrane glycerophosphodiester transmembrane transporter (predicted)	0.32	4.67	2.54E-05	1.20E-03
SPBC28E12.06c	lvs1	beige protein homolog Lvs1	0.41	4.68	2.52E-05	1.20E-03
SPBC337.11	SPBC337.11	mitochondrial inner membrane CH-OH group oxidoreductase family, reticulon interacting protein, implicated in mitochondrial organization or tethering	0.52	4.65	2.78E-05	1.25E-03
SPBC725.15	ura5	orotate phosphoribosyltransferase Ura5	0.4	4.64	2.85E-05	1.25E-03
SPBC32F12.09	rum1	CDK inhibitor Rum1	0.34	4.58	3.50E-05	1.50E-03
SPAC13C5.05c	SPAC13C5.05c	N-acetylglucosamine-phosphate mutase (predicted)	0.32	4.54	3.91E-05	1.64E-03
SPCC548.05c	dbl5	ubiquitin-protein ligase E3 Dbl5	0.45	4.51	4.39E-05	1.79E-03
SPBC4.06	SPBC4.06	acid phosphatase Fmp10 (predicted)	0.31	4.47	4.91E-05	1.92E-03
SPAC222.07c	hri2	eIF2 alpha kinase Hri2	0.4	4.42	5.77E-05	2.11E-03
SPBC18H10.13	rps1402	40S ribosomal protein S14 (predicted)	0.3	4.41	5.99E-05	2.16E-03
SPBC3B9.06c	atg3	autophagy associated protein Atg3	0.45	4.41	6.13E-05	2.18E-03
SPBC11C11.06c	SPBC11C11.06c	Schizosaccharomyces specific protein	0.34	4.4	6.31E-05	2.19E-03
SPBC6B1.02	ppk30	Ark1/Prk1 family protein kinase Ppk30	1	4.27	9.42E-05	2.81E-03
SPBC30D10.18c	rpl102	60S ribosomal protein L10a	0.36	4.27	9.62E-05	2.83E-03
SPBP26C9.03c	fet4	plasma membrane iron/zinc ion transmembrane transporter (predicted)	0.31	4.23	1.09E-04	3.10E-03
SPBC839.06	cta3	P-type ATPase, potassium exporting Cta3	0.5	4.2	1.19E-04	3.26E-03
SPBC21D10.07	cmc1	copper-binding protein of the mitochondrial intermembrane space Cmc1 (predicted)	0.41	4.13	1.49E-04	3.72E-03
SPCC18.09c	hnt3	aprataxin Hnt3	0.52	4.13	1.48E-04	3.72E-03
SPBP8B7.24c	atg8	autophagy associated protein Atg8	0.31	4.12	1.55E-04	3.76E-03
SPCC794.15	SPCC794.15	Schizosaccharomyces specific protein	0.43	4.13	1.65E-04	3.93E-03
SPBC12C2.12c	glo1	glyoxalase I	0.29	4.09	1.71E-04	3.98E-03
SPBC19C2.02	pmt1	tRNA (cytosine-5-)-methyltransferase Pmt1	0.29	3.98	2.37E-04	5.37E-03
SPAC3G6.03c	SPAC3G6.03c	Maf-like protein, nucleoside-triphosphate diphosphatase, human ASMTL ortholog	0.35	3.95	2.63E-04	5.76E-03
SPBC4F6.05c	emp46	lectin family glycoprotein receptor Emp46 (predicted)	0.41	3.93	2.76E-04	5.97E-03
SPAPB24D3.01	toe3	transcription factor (predicted)	0.37	3.88	3.26E-04	6.83E-03
SPBC19C7.08c	ppm2	tRNA methyltransferase Ppm2 (predicted)	0.28	3.78	4.44E-04	8.81E-03
SPBC1734.12c	alg12	dolichyl pyrophosphate Man7GlcNAc2 alpha-1,6-mannosyltransferase Alg12 (predicted)	0.25	3.77	4.54E-04	8.95E-03
SPBC83.19c	SPBC83.19c	Schizosaccharomyces pombe specific protein	0.25	3.8	4.59E-04	8.97E-03
SPAC13G6.03	gpi7	GPI anchor biosynthesis protein Gpi7 (predicted)	0.45	3.76	4.66E-04	8.97E-03
SPAC1751.01c	gti1	gluconate transmembrane transporter inducer Gti1	0.53	3.71	5.58E-04	1.04E-02
SPBC83.05	SPBC83.05	mitochondrial RNA-binding protein (predicted)	0.74	3.7	5.66E-04	1.04E-02

SPBP4H10.09	rsv1	transcription factor Rsv1		0.5	3.7	5.63E-04	1.04E-02
SPAC1420.01c	SPAC1420.01c	GATA-like domain protein (predicted)		0.5	3.65	6.57E-04	1.16E-02
SPAC1834.05	alg9	mannosyltransferase complex subunit Alg9 (predicted)		0.3	3.67	6.69E-04	1.17E-02
SPBC2G5.01	SPBC2G5.01	ER protein involved in ER-nucleus signaling (predicted)		0.42	3.64	6.76E-04	1.18E-02
SPBC21C3.09c	oaa1	mitochondrial acylpyruvase Oaa1 (predicted)		0.38	3.64	6.85E-04	1.19E-02
SPAC17G6.15c	fsf1	mitochondrial carrier, serine Fsf1 (predicted)		0.23	3.6	7.75E-04	1.30E-02
SPCC70.02c	inh1	mitochondrial proton-transporting ATP synthase inhibitor Inh1 (predicted)		0.33	3.58	8.06E-04	1.31E-02
SPBC839.07	ibp1	Cdc25 family phosphatase Ibp1, unknown role, implicated in DNA replication		0.7	3.57	8.36E-04	1.33E-02
SPCC757.13	SPCC757.13	dipeptide transmembrane transporter (predicted)		0.39	3.57	8.33E-04	1.33E-02
SPBC29A3.18	cyt1	cytochrome c1 Cyt1 (predicted)		0.22	3.56	8.73E-04	1.38E-02
SPAC22E12.05c	rer1	Rer1 family protein (predicted)		0.23	3.54	9.05E-04	1.40E-02
SPBC1861.02	abp2	unknown protein, may bind replication origins Abp2		0.31	3.54	9.18E-04	1.40E-02
SPBC27B12.05	SPBC27B12.05	WD repeat protein		0.28	3.54	9.28E-04	1.41E-02
SPAC1F7.12	yak3	aldose reductase ARK13 family YakC, implicated in cellular detoxification from family members		0.31	3.53	9.58E-04	1.45E-02
SPAC8F11.08c	SPAC8F11.08c	ER membrane associated esterase/lipase (predicted)		0.27	3.51	9.95E-04	1.48E-02
SPBC18E5.07	aim21	barbed end F-actin assembly inhibitor		0.32	3.51	1.02E-03	1.50E-02
SPBC1683.12	SPBC1683.12	carboxylic acid transmembrane transporter (predicted)		0.39	3.5	1.05E-03	1.53E-02
SPAC6G9.12	cfr1	exomer complex BRCT domain subunit Cfr1		0.24	3.48	1.10E-03	1.57E-02
SPBC31E1.01c	atg2	autophagy associated protein Atg2		0.33	3.48	1.10E-03	1.57E-02
SPAC589.10c	ubi5	ribosomal-ubiquitin fusion protein Ubi5 (predicted)		0.4	3.44	1.25E-03	1.76E-02
SPAPB8E5.04c	npc2	Niemann-Pick disease type C2 protein hE1 homolog Npc2 (predicted)		0.24	3.43	1.28E-03	1.79E-02
SPBC691.03c	apl3	AP-2 adaptor complex alpha subunit Alp3		0.37	3.43	1.28E-03	1.79E-02
SPAC323.05c	mtq2	eRF1 methyltransferase Mtq2 (predicted)		0.22	3.42	1.32E-03	1.84E-02
SPBC1734.09	yea4	ER UDP-N-acetylglucosamine transmembrane transporter (predicted)		0.34	3.42	1.33E-03	1.84E-02
SPCC18B5.11c	cds1	replication checkpoint kinase Cds1		0.38	3.4	1.37E-03	1.88E-02
SPBC887.17	SPBC887.17	nucleobase transmembrane transporter (predicted)		0.4	3.38	1.49E-03	2.02E-02
SPAC186.08c	SPAC186.08c	L-lactate dehydrogenase (predicted)		0.31	3.37	1.51E-03	2.04E-02
SPBPB2B2.07c	SPBPB2B2.07c	S. pombe specific DUF999 protein family 7		0.29	3.36	1.54E-03	2.06E-02
SPBC8D2.18c	SPBC8D2.18c	adenosylhomocysteinase (predicted)		0.24	3.36	1.55E-03	2.07E-02
SPBC16E9.17c	rem1	meiosis-specific cyclin Rem1		0.26	3.35	1.61E-03	2.11E-02
SPBC1198.07c	SPBC1198.07c	mannan endo-1,6-alpha-mannosidase (predicted)		0.22	3.34	1.64E-03	2.14E-02
SPBC902.05c	idh2	isocitrate dehydrogenase (NAD+) subunit 2		0.31	3.34	1.66E-03	2.14E-02
SPAC3A11.05c	kms1	meiotic spindle pole body KASH domain protein Kms1		0.27	3.33	1.69E-03	2.18E-02
SPAC14C4.05c	man1	LEM domain nuclear inner membrane protein Man1, Sad1 interacting factor		0.28	3.28	2.05E-03	2.48E-02
SPBC16E9.03c	coa1	cytochrome c oxidase assembly protein Coa1 (predicted)		0.24	3.27	2.02E-03	2.48E-02
SPCC594.06c	vsl1	vacuolar SNARE Vsl1/Vam7		0.28	3.27	2.03E-03	2.48E-02
SPBC3E7.16c	leu3	2-isopropylmalate synthase Leu3		0.2	3.25	2.15E-03	2.57E-02
SPAC23D3.03c	SPAC23D3.03c	GTPase activating protein (predicted)		0.31	3.25	2.22E-03	2.62E-02
SPAC1805.15c	pub2	HECT-type ubiquitin-protein ligase E3 Pub2		0.24	3.23	2.30E-03	2.69E-02
SPAC1B3.15c	SPAC1B3.15c	transmembrane transporter (predicted)		0.22	3.22	2.35E-03	2.71E-02
SPCC330.07c	SPCC330.07c	transmembrane transporter (predicted)		0.25	3.22	2.34E-03	2.71E-02
SPBC16E9.02c	SPBC16E9.02c	CUE domain protein, human TOLLIP ortholog		0.27	3.19	2.54E-03	2.84E-02
SPBC2D10.04	aly2	arrestin Aly1 related Aly2		0.31	3.17	2.68E-03	2.97E-02
SPAC12B10.07	acp1	F-actin capping protein alpha subunit		0.21	3.18	2.76E-03	3.04E-02
SPAC18B11.08c	SPAC18B11.08c	UPF0139 family conserved fungal ER membrane protein		0.29	3.16	2.78E-03	3.05E-02
SPAC19B12.08	atg4	Atg8 deconjugator Atg4 (predicted)		0.26	3.16	2.87E-03	3.11E-02
SPBC800.11	SPBC800.11	inosine-uridine preferring nucleoside hydrolase (predicted)		0.27	3.15	2.87E-03	3.11E-02
SPBC365.11	grp2	Golgi GRIP domain protein Grp2 (predicted)		0.2	3.14	2.96E-03	3.18E-02
SPCC1183.06	ung1	uracil DNA N-glycosylase Ung1		0.26	3.14	2.96E-03	3.18E-02
SPCC16C4.01	sif2	mitochondrial protein, involved in mitochondrial gene expression (predicted)		0.3	3.13	3.01E-03	3.22E-02
SPCC1682.11c	ctl1	choline transporter-like, implicated in autophagy Ctl1		0.28	3.13	3.03E-03	3.23E-02
SPAC3H8.04	SPAC3H8.04	DUF4210 domain protein, human FAM214A ortholog, implicated in chromosome segregation (predicted)		0.23	3.12	3.12E-03	3.30E-02
SPBC1709.13c	set10	ribosomal lysine methyltransferase Set10		0.22	3.11	3.15E-03	3.32E-02
SPCC757.02c	SPCC757.02c	dehydrogenase (predicted)		0.19	3.1	3.30E-03	3.42E-02

SPCC330.14c	rpl2402	60S ribosomal protein L24 (predicted)	0.27	3.09	3.33E-03	3.43E-02
SPBC24C6.08c	bhd1	Lst4-Lst7 complex subunit, folliculin Bhd1/Lst7	0.45	3.08	3.46E-03	3.52E-02
SPBC25B2.11	pof2	F-box protein Pof2	0.25	3.06	3.62E-03	3.64E-02
SPAC23C4.12	hhp2	serine/threonine protein kinase Hhp2	0.27	3.06	3.68E-03	3.68E-02
SPAC16C9.02c	mta1	S-methyl-5-thioadenosine phosphorylase Mta1	0.21	3.05	3.75E-03	3.70E-02
SPAC20G4.01	caf16	CCR4-Not complex subunit Caf16 (predicted)	0.23	3.04	3.92E-03	3.81E-02
SPCC4G3.17	hdd1	GMP 5'-nucleotidase (predicted)	0.21	3.04	3.90E-03	3.81E-02
SPAC17A2.14	mnr2	vacuolar CorA family magnesium ion transmembrane transporter Mnr2	0.24	3.04	4.04E-03	3.92E-02
SPAC1F8.05	isp3	spore wall structural constituent Isp3	0.3	3.02	4.08E-03	3.94E-02
SPCC364.02c	bis1	splicing factor Bis1	0.27	3.02	4.13E-03	3.97E-02
SPBP4G3.03	fub2	PI31 proteasome regulator Fub2 (predicted)	0.23	2.97	4.70E-03	4.34E-02
SPCC320.07c	mde7	RNA-binding protein Mde7	0.23	2.97	4.69E-03	4.34E-02
SPAC22G7.02	kap111	karyopherin/importin beta family nuclear import signal receptor Kap111 (predicted)	0.27	2.98	4.74E-03	4.35E-02
SPAC23A1.03	apt1	adenine phosphoribosyltransferase (APRT) Apt1	0.28	2.98	4.79E-03	4.36E-02
SPAC144.05	SPAC144.05	DNA-dependent ATPase/ ubiquitin-protein ligase E3 (predicted)	0.21	2.97	4.82E-03	4.36E-02
SPAC19B12.11c	bud20	zinc finger ribosome biogenesis protein Bud20 (predicted)	0.26	2.97	4.90E-03	4.42E-02
SPBC17A3.02	aim19	mitochondrial conserved fungal membrane protein Aim19	0.23	2.93	5.23E-03	4.69E-02
SPBC359.04c	pfl7	cell surface glycoprotein, flocculin Pfl7, DIPSY family	0.25	2.93	5.28E-03	4.72E-02
SPAC56F8.14c	mug115	Schizosaccharomyces pombe specific protein Mug115	0.34	2.92	5.40E-03	4.76E-02
SPBC8E4.03	SPBC8E4.03	agmatinase 2 (predicted)	0.25	2.91	5.54E-03	4.80E-02
SPAC30.03c	tsn1	translin	0.23	2.91	5.72E-03	4.89E-02
SPAC14C4.04	SPAC14C4.04	ThiJ domain protein (predicted)	0.23	2.89	5.76E-03	4.90E-02
SPBC800.12c	SPBC800.12c	ubiquitin family protein (predicted)	0.24	2.89	5.87E-03	4.98E-02
