# Supplementary material for: Global network analysis in Schizosaccharomyces pombe reveals three distinct consequences of the common 1-kb deletion causing juvenile CLN3 disease
Source: Sci Rep. 2021 Mar 18;11:6332. doi: 10.1038/s41598-021-85471-4 (PMC7973434; doi:10.1038/s41598-021-85471-4)
Supplement: Supplementary file 5 — S5: Supplementary Tables 5. [file 41598_2021_85471_MOESM5_ESM.pdf]

Global network analysis in *Schizosaccharomyces pombe* reveals three distinct consequences of the common 1-kb deletion causing juvenile CLN3 disease

Christopher J. Minnis[1,2], StJohn Townsend[3,4], Julia Petschnigg[1], Elisa Tinelli[1], Jürg Bähler[3], Claire Russell[2], Sara E. Mole[1]

[1]MRC Laboratory for Molecular Cell Biology and Great Ormond Street Institute of Child Health, University College London, London WC1E 6BT, UK
[2]Dept. Comparative Biomedical Sciences, Royal Veterinary College, Royal College Street, London NW1 0TU, UK
[3]Institute of Healthy Ageing, Department of Genetics, Evolution and Environment, University College London, London WC1E 6BT, UK
[4]The Molecular Biology of Metabolism Laboratory, The Francis Crick Institute, London, NW1 1AT, United Kingdom

*Corresponding author: christopher.minnis.15@ucl.ac.uk

Supplementary table 1 : Positive colony size difference between *btn1Δ* vs *btn1(D363G)*

Systematic ID	Gene name	Product description	Colony Size Difference	t	P Value	Adjusted P Value
SPBC337.08c	ubi4	protein modifier, ubiquitin	1.1	5.44	1.93E-06	5.02E-03

Supplementary table 2 : Negative colony size difference between *btn1Δ* vs *btn1(102-208del)*

Systematic ID	Gene name	Product description	Colony Size Difference	t	P Value	Adjusted P Value
SPBC3H7.03c	kgd1	2-oxoglutarate dehydrogenase (lipoamide) (e1 component of oxoglutarate dehydrogenase complex) (predicted)	-0.64	-6.37	7.53E-08	9.79E-05
SPBC1D7.03	clg1	cyclin-like protein involved in autophagy Clg1 (predicted)	-0.4	-5.79	5.62E-07	3.65E-04
SPBC543.09	yta12	mitochondrial m-AAA protease Yta12 (predicted)	-0.54	-5.57	1.20E-06	5.22E-04
SPBC530.08	SPBC530.08	membrane-tethered transcription factor (predicted)	-0.42	-5.53	1.42E-06	5.26E-04
SPAC3G9.03	rpl2301	60S ribosomal protein L23	-0.42	-5.4	2.15E-06	5.60E-04
SPAC823.10c	hem25	mitochondrial carrier, glycine Hem25 (predicted)	-0.56	-5.4	2.15E-06	5.60E-04
SPCC1393.08	fil1	transcription factor, zf-GATA type	-0.46	-5.33	2.79E-06	6.59E-04
SPCC18B5.10c	tex1	TREX complex subunit Tex1 (predicted)	-0.64	-5.23	3.85E-06	7.71E-04
SPBC4B4.03	rsc1	RSC complex subunit Rsc1	-0.53	-5.18	4.61E-06	8.57E-04
SPBP8B7.05c	nce103	carbonic anhydrase (predicted)	-0.53	-5.1	6.16E-06	1.07E-03
SPBC947.08c	hip4	histone promoter control protein Hip4	-0.57	-4.92	1.12E-05	1.71E-03
SPCC126.04c	sgf73	SAGA complex deubiquitinating submodule subunit Sgf73	-0.48	-4.85	1.42E-05	1.85E-03
SPBC530.06c	clu1	clustered mitochndria ortholog Clu1	-0.58	-4.81	1.64E-05	2.03E-03
SPCC1739.10	mug33	Tea1-interacting protein involved in exocytosis	-0.52	-4.76	1.91E-05	2.16E-03
SPBC19F8.08	rps401	40S ribosomal protein S4 (predicted)	-0.39	-4.73	2.13E-05	2.21E-03
SPBC12C2.04	SPBC12C2.04	NAD binding dehydrogenase family protein	-0.37	-4.71	2.23E-05	2.23E-03
SPAC13G6.15c	SPAC13G6.15c	calcipressin (predicted)	-0.5	-4.68	2.49E-05	2.30E-03
SPAC19B12.10	sst2	human AMSH/STAMBP protein homolog, ubiquitin specific-protease	-0.64	-4.72	2.56E-05	2.30E-03
SPBC543.07	pek1	MAP kinase kinase Pek1	-0.38	-4.69	2.42E-05	2.30E-03
SPBC2D10.13	est1	telomerase regulator Est1	-0.4	-4.65	2.70E-05	2.34E-03
SPAC13G6.14	aps1	diadenosine 5',5'''-p1,p6-hexaphosphate hydrolase Aps1	-0.87	-4.6	3.22E-05	2.65E-03
SPCC16C4.10	SPCC16C4.10	6-phosphogluconolactonase (predicted)	-0.29	-4.56	3.67E-05	2.65E-03
SPCC594.05c	spf1	Set1C PHD finger subunit Spf1	-0.35	-4.57	3.52E-05	2.65E-03
SPAC26H5.10c	tif51	translation elongation and termination factor eIF5A (predicted)	-0.4	-4.43	5.58E-05	3.82E-03
SPCC18B5.03	wee1	M phase inhibitor protein kinase Wee1	-0.45	-4.39	6.55E-05	4.26E-03
SPAC6F12.02	rst2	transcription factor Rst2	-0.53	-4.34	7.59E-05	4.59E-03
SPBC1105.10	rav1	RAVE complex subunit Rav1	-0.3	-4.33	7.84E-05	4.63E-03
SPAC13G6.10c	asl1	cell wall protein Asl1, predicted O-glucosyl hydrolase	-0.65	-4.32	8.16E-05	4.71E-03
SPAPB2B4.02	grx5	mitochondrial [2Fe-2S] cluster assembly and transfer glutaredoxin Grx5	-0.29	-4.35	8.41E-05	4.75E-03
SPBC839.15c	tef103	translation elongation factor EF-1 alpha Ef1a-c	-0.57	-4.24	1.04E-04	5.76E-03
SPBC3H7.15	hhp1	serine/threonine protein kinase Hhp1	-0.28	-4.21	1.14E-04	5.88E-03
SPBP16F5.05c	yar1	ribosome biogenesis protein Yar1 (predicted)	-0.39	-4.21	1.15E-04	5.88E-03
SPBPB2B2.13	gal1	galactokinase Gal1	-0.38	-4.22	1.11E-04	5.88E-03
SPBC1A4.05	blt1	ubiquitin domain-like protein Blt1	-0.43	-4.19	1.22E-04	6.10E-03
SPBC3D6.04c	mad1	mitotic spindle checkpoint protein Mad1	-0.4	-4.14	1.45E-04	6.85E-03
SPBC887.10	mcs4	response regulator Mcs4	-0.65	-4.14	1.43E-04	6.85E-03
SPAC1071.04c	spc2	signal peptidase subunit Spc2 (predicted)	-0.35	-4.07	1.79E-04	8.16E-03
SPBC16G5.15c	fkh2	forkhead transcription factor Fkh2	-0.41	-4.06	1.86E-04	8.35E-03
SPAC644.06c	cdr1	NIM1 family serine/threonine protein kinase Cdr1/Nim1	-0.82	-4	2.23E-04	9.84E-03
SPBC21C3.08c	car2	ornithine transaminase Car2	-0.71	-4	2.27E-04	9.84E-03
SPBC887.15c	sur2	sphingosine hydroxylase/sphingolipid delta-4 desaturase activity Sur2	-0.33	-3.99	2.32E-04	9.85E-03
SPAC8E11.05c	SPAC8E11.05c	DUF5102 family conserved fungal protein, associated with clathrin coated vesicles (predicted)	-0.36	-3.94	2.69E-04	1.11E-02
SPAC19G12.13c	poz1	shelterin complex subunit Poz1	-0.47	-3.9	3.10E-04	1.22E-02
SPBC1A4.04	SPBC1A4.04	Schizosaccharomyces specific protein	-0.35	-3.89	3.15E-04	1.22E-02
SPBC1778.03c	SPBC1778.03c	NADH pyrophosphatase (predicted)	-0.33	-3.89	3.21E-04	1.23E-02
SPCC18.17c	SPCC18.17c	proteasome assembly chaperone (predicted)	-0.3	-3.86	3.44E-04	1.27E-02

SPAC2F7.04	med1	mediator complex subunit Med1	-0.37	-3.85	3.58E-04	1.29E-02
SPCC757.05c	SPCC757.05c	peptidase family M20 protein involved in glutathione catabolism (predicted)	-0.37	-3.84	3.65E-04	1.29E-02
SPCP1E11.10	dhm1	ankyrin repeat protein, unknown biological role	-0.29	-3.84	3.72E-04	1.29E-02
SPBC19C2.13c	ctu2	cytosolic thiouridylase subunit Ctu2	-0.28	-3.83	3.81E-04	1.30E-02
SPAC8F11.02c	dph3	diphthamide biosynthesis protein Dph3 (predicted)	-0.53	-3.82	3.90E-04	1.32E-02
SPAC23A1.19c	hrq1	RecQ type DNA helicase Hrq1 (predicted)	-0.46	-3.79	4.28E-04	1.37E-02
SPCC794.11c	ent3	ENTH/VHS domain protein Ent3 (predicted)	-0.34	-3.8	4.21E-04	1.37E-02
SPBC25H2.16c	gga22	Golgi localized Arf binding gamma-adaptin ortholog Gga22	-0.27	-3.75	4.84E-04	1.50E-02
SPBC16E9.16c	lsd90	Lsd90 protein	-0.51	-3.72	5.30E-04	1.60E-02
SPAC16E8.05c	SPAC16E8.05c	Schizosaccharomyces specific protein Mde1	-0.39	-3.7	5.62E-04	1.68E-02
SPCC1739.14	npp106	nucleoporin Npp106	-0.3	-3.69	5.92E-04	1.73E-02
SPBC2F12.11c	rep2	MBF transcription factor activator Rep2	-0.58	-3.66	6.39E-04	1.82E-02
SPBC32F12.01c	css1	inositol phosphosphingolipid phospholipase C, Css1	-0.39	-3.65	6.53E-04	1.85E-02
SPBC17A3.09c	aim22	lipoate-protein ligase A (predicted)	-0.38	-3.64	6.73E-04	1.86E-02
SPCC794.08	efr3	phosphatidylinositol-4 kinase plasma membrane scaffold Efr3	-0.33	-3.61	7.46E-04	2.02E-02
SPAC22F3.08c	rok1	ATP-dependent RNA helicase Rok1 (predicted)	-0.54	-3.6	8.23E-04	2.10E-02
SPAC22F8.02c	pvg5	Golgi 4,6-pyruvylated galactose (PvGal) residue biosynthesis protein Pvg5	-0.31	-3.58	8.10E-04	2.10E-02
SPBC1604.02c	ppr1	mitochondrial PPR repeat protein Ppr1	-0.31	-3.59	7.86E-04	2.10E-02
SPBC409.11	meu18	Schizosaccharomyces specific protein Meu18	-0.28	-3.59	7.95E-04	2.10E-02
SPBC887.05c	cwf29	RNA-binding protein Cwf29	-0.44	-3.58	8.04E-04	2.10E-02
SPBC27B12.03c	erg32	C-5 sterol desaturase Erg32	-0.38	-3.55	8.85E-04	2.21E-02
SPAPJ696.01c	vps17	retromer complex subunit Vps17	-0.52	-3.55	9.03E-04	2.24E-02
SPAC2F7.17	mrf1	mitochondrial translation release factor (predicted)	-0.23	-3.52	9.78E-04	2.29E-02
SPBC3E7.01	fab1	1-phosphatidylinositol-3-phosphate 5-kinase Fab1	-0.42	-3.53	9.51E-04	2.29E-02
SPBC365.10	arp5	Ino80 complex actin-like protein Arp5	-0.33	-3.49	1.06E-03	2.41E-02
SPBC685.06	rps001	40S ribosomal protein S0A (p40)	-0.29	-3.47	1.12E-03	2.46E-02
SPCC1682.12c	ubp16	ubiquitin C-terminal hydrolase Ubp16	-0.34	-3.48	1.11E-03	2.46E-02
SPAP8A3.14c	sls1	mitochondrial inner membrane protein Sls1 (predicted)	-0.33	-3.46	1.15E-03	2.51E-02
SPAC1071.02	mms19	CIA machinery protein Mms19	-0.86	-3.46	1.24E-03	2.57E-02
SPAC8E11.07c	alp31	tubulin specific chaperone cofactor A, Alp31	-0.31	-3.46	1.24E-03	2.57E-02
SPBC119.12	rud3	Golgi matrix protein Rud3 (predicted)	-0.35	-3.43	1.29E-03	2.57E-02
SPBC16H5.08c	SPBC16H5.08c	ribosome biogenesis ATPase, Arb family ABCF2-like (predicted)	-0.3	-3.43	1.27E-03	2.57E-02
SPBC21B10.06c	inp2	myosin binding vezatin family protein involved in peroxisome inheritance Inp2 (predicted)	-0.33	-3.42	1.30E-03	2.57E-02
SPCC4F11.03c	SPCC4F11.03c	Schizosaccharomyces specific protein	-0.23	-3.43	1.29E-03	2.57E-02
SPBC18H10.19	vps38	phophatidylinositol 3-kinase complex subunit Vps38	-0.26	-3.42	1.31E-03	2.59E-02
SPBC8D2.17	gmh4	alpha-1,2-galactosyltransferase (predicted)	-0.31	-3.42	1.33E-03	2.60E-02
SPBC3E7.10	fma1	methionine aminopeptidase Fma1 (predicted)	-0.32	-3.41	1.36E-03	2.60E-02
SPCC285.17	spp27	RNA polymerase I upstream activation factor complex subunit Spp27	-0.52	-3.4	1.38E-03	2.62E-02
SPAC16C9.05	cph1	Clr6 histone deacetylase associated PHD finger protein Cph1	-0.48	-3.41	1.42E-03	2.68E-02
SPCC794.03	SPCC794.03	amino acid transmembrane transporter (predicted)	-0.32	-3.38	1.48E-03	2.75E-02
SPAC1071.07c	rps1502	40S ribosomal protein S15 (predicted)	-0.31	-3.37	1.50E-03	2.76E-02
SPAC26A3.07c	rpl1101	60S ribosomal protein L11 (predicted)	-0.38	-3.37	1.53E-03	2.79E-02
SPAC144.03	ade2	adenylosuccinate synthetase Ade2	-0.36	-3.36	1.58E-03	2.88E-02
SPAC1F3.03	sro7	Lgl family protein Sro7 (predicted)	-0.38	-3.32	1.75E-03	3.10E-02
SPBC27B12.10c	tom7	mitochondrial TOM complex subunit Tom7 (predicted)	-0.23	-3.32	1.76E-03	3.10E-02
SPCC16C4.20c	hap2	Ino80 complex, HMG box protein Hap2	-0.39	-3.32	1.77E-03	3.10E-02
SPBC16E9.18	psd1	phosphatidylserine decarboxylase Psd1	-0.31	-3.31	1.81E-03	3.16E-02
SPBC23E6.09	ssn6	transcriptional corepressor Ssn6	-0.79	-3.29	1.89E-03	3.26E-02
SPAC22G7.05	kri1	ribosome biogenesis protein Kri1 (predicted)	-0.53	-3.27	2.00E-03	3.40E-02
SPBC21B10.03c	ath1	ataxin-2 homolog	-0.34	-3.26	2.07E-03	3.49E-02
SPAC222.05c	mss1	mitochondrial tRNA wobble uridine modification GTPase Mss1 (predicted)	-0.22	-3.26	2.10E-03	3.51E-02
SPAC227.17c	SPAC227.17c	DUF3128 family, human c22orf39 ortholog	-0.35	-3.25	2.14E-03	3.52E-02
SPBC28F2.10c	ngg1	SAGA complex subunit Ngg1/Ada3	-0.27	-3.24	2.22E-03	3.61E-02
SPAC1A6.04c	plb1	phospholipase B homolog Plb1	-0.42	-3.23	2.26E-03	3.65E-02

SPBC354.05c	sre2	membrane-tethered transcription factor Sre2	-0.48	-3.21	2.39E-03	3.81E-02
SPBC11G11.01	fis1	mitochondrial fission protein Fis1 (predicted)	-0.28	-3.2	2.49E-03	3.86E-02
SPBC1685.13	fhn1	eisosome assembly protein Fhn1	-0.34	-3.2	2.48E-03	3.86E-02
SPBC660.07	ntp1	alpha,alpha-trehalase Ntp1	-0.42	-3.2	2.47E-03	3.86E-02
SPAC1039.08	SPAC1039.08	serine acetyltransferase (predicted)	-0.41	-3.17	2.66E-03	4.10E-02
SPBC106.16	pre6	20S proteasome complex subunit alpha 4 Pre6	-0.52	-3.16	2.79E-03	4.24E-02
SPBC4F6.06	kin1	microtubule affinity-regulating kinase Kin1	-0.3	-3.14	2.93E-03	4.36E-02
SPAC1B3.07c	vps28	ESCRT I complex subunit Vps28	-0.23	-3.13	3.05E-03	4.45E-02
SPAC144.06	apl5	AP-3 adaptor complex subunit Apl5 (predicted)	-0.26	-3.12	3.13E-03	4.51E-02
SPAC31G5.04	lys12	homoisocitrate dehydrogenase Lys12	-0.21	-3.12	3.14E-03	4.51E-02
SPCC1919.05	ski3	Ski complex TPR repeat subunit Ski3 (predicted)	-0.22	-3.1	3.32E-03	4.71E-02
SPCC364.03	rpl1702	60S ribosomal protein L17 (predicted)	-0.37	-3.09	3.35E-03	4.74E-02
SPAC212.03	SPAC212.03	hypothetical protein	-0.45	-3.1	3.38E-03	4.75E-02
SPAP8A3.13c	SPAP8A3.13c	Vid24 family protein (predicted)	-0.25	-3.08	3.51E-03	4.86E-02
SPBCPT2R1.08c	tlh2	RecQ type DNA helicase Tlh1	-0.28	-3.09	3.50E-03	4.86E-02
SPAPB17E12.05	rpl3703	60S ribosomal protein L37 (predicted)	-0.43	-3.08	3.61E-03	4.94E-02
SPAC29A4.20	elp3	elongator complex subunit Elp3	-0.24	-3.06	3.70E-03	4.97E-02
SPAC8E11.02c	rad24	14-3-3 protein Rad24	-0.24	-3.06	3.66E-03	4.97E-02

Supplementary table 3 : Positive colony size difference between *btn1Δ* vs *btn1(102-208del)*

Systematic ID	Gene name	Product description	Colony Size Difference	t	P Value	Adjusted P Value
SPBC29A3.21	SPBC29A3.21	Schizosaccharomyces pombe specific protein	1.39	9.03	1.84E-11	4.78E-08
SPBC211.06	gfh1	gamma tubulin complex subunit Gfh1	0.48	6.24	1.18E-07	1.02E-04
SPBC32F12.09	rum1	CDK inhibitor Rum1	0.42	5.61	1.07E-06	5.22E-04
SPBC418.01c	his4	imidazoleglycerol-phosphate synthase His4	0.46	5.42	2.04E-06	5.60E-04
SPBC1271.09	tgp1	plasma membrane glycerophosphodiester transmembrane transporter (predicted)	0.36	5.26	3.50E-06	7.58E-04
SPCPB1C11.02	SPCPB1C11.02	amino acid transmembrane transporter (predicted)	0.35	4.97	9.43E-06	1.53E-03
SPAC18B11.04	ncs1	neuronal calcium sensor related protein Ncs1	0.34	4.86	1.37E-05	1.85E-03
SPBC4.06	SPBC4.06	acid phosphatase Fmp10 (predicted)	0.34	4.86	1.36E-05	1.85E-03
SPBC839.11c	hut1	ER uridine diphosphate-glucose transmembrane transporter Hut1	0.36	4.79	1.73E-05	2.04E-03
SPAC222.07c	hri2	eIF2 alpha kinase Hri2	0.42	4.75	2.00E-05	2.17E-03
SPBC3B9.06c	atg3	autophagy associated protein Atg3	0.47	4.57	3.63E-05	2.65E-03
SPBC405.05	atg16	autophagy associated protein Atg16	0.3	4.59	3.34E-05	2.65E-03
SPBC8D2.01	gsk31	serine/threonine protein kinase Gsk31 (predicted)	0.59	4.58	3.46E-05	2.65E-03
SPAC11D3.04c	SPAC11D3.04c	polyketide cyclase SnoaL-like domain protein	0.61	4.47	4.92E-05	3.46E-03
SPBP8B7.24c	atg8	autophagy associated protein Atg8	0.34	4.42	5.84E-05	3.89E-03
SPBP26C9.03c	fet4	plasma membrane iron/zinc ion transmembrane transporter (predicted)	0.32	4.37	6.82E-05	4.33E-03
SPBC16E9.17c	rem1	meiosis-specific cyclin Rem1	0.33	4.35	7.26E-05	4.50E-03
SPBC12C2.12c	glo1	glyoxalase I	0.29	4.22	1.12E-04	5.88E-03
SPAC3A11.05c	kms1	meiotic spindle pole body KASH domain protein Kms1	0.33	4.15	1.41E-04	6.85E-03
SPAC23C4.12	hhp2	serine/threonine protein kinase Hhp2	0.36	4.11	1.58E-04	7.32E-03
SPBC16E9.11c	pub3	HECT-type ubiquitin-protein ligase E3 Pub3 (predicted)	0.29	3.99	2.35E-04	9.85E-03
SPBC29A3.18	cyt1	cytochrome c1 Cyt1 (predicted)	0.25	3.94	2.75E-04	1.12E-02
SPAC57A7.09	SPAC57A7.09	ubiquitin-protein ligase E3, human RNF13 family homolog, unknown biological role	0.26	3.93	2.84E-04	1.13E-02
SPBC16E9.02c	SPBC16E9.02c	CUE domain protein, human TOLLIP ortholog	0.33	3.88	3.28E-04	1.24E-02
SPBC18E5.07	aim21	barbed end F-actin assembly inhibitor	0.35	3.86	3.47E-04	1.27E-02
SPBC6B1.02	ppk30	Ark1/Prk1 family protein kinase Ppk30	0.9	3.84	3.68E-04	1.29E-02
SPAC1039.03	SPAC1039.03	esterase/lipase, implicated in cellular detoxification (predicted)	0.24	3.8	4.15E-04	1.37E-02
SPAC1952.09c	SPAC1952.09c	acetyl-CoA hydrolase (predicted)	0.27	3.79	4.31E-04	1.37E-02
SPBC354.15	fap1	L-pipecolate oxidase	0.3	3.79	4.32E-04	1.37E-02
SPCC70.08c	SPCC70.08c	methyltransferase (predicted)	0.55	3.78	4.39E-04	1.38E-02
SPBC19C2.02	pmt1	tRNA (cytosine-5-)-methyltransferase Pmt1	0.27	3.74	5.01E-04	1.53E-02
SPAC1F7.12	yak3	aldose reductase ARK13 family YakC, implicated in cellular detoxification from family members	0.33	3.69	5.85E-04	1.73E-02

SPCC757.11c	SPCC757.11c	transmembrane transporter (predicted)	0.31	3.67	6.19E-04	1.79E-02
SPBC609.02	ptn1	phosphatidylinositol-3,4,5-trisphosphate3-phosphatase Ptn1	0.71	3.64	6.73E-04	1.86E-02
SPAC22G7.02	kap111	karyopherin/importin beta family nuclear import signal receptor Kap111 (predicted)	0.33	3.66	7.00E-04	1.91E-02
SPBC1198.07c	SPBC1198.07c	mannan endo-1,6-alpha-mannosidase (predicted)	0.24	3.58	8.22E-04	2.10E-02
SPAC1420.01c	SPAC1420.01c	GATA-like domain protein (predicted)	0.48	3.56	8.60E-04	2.17E-02
SPBC23G7.06c	nvj2	nucleus-vacuole junction protein Nvj2	0.27	3.54	9.32E-04	2.29E-02
SPAC14C4.11	vtc2	vacuolar transporter chaperone (VTC) complex polyphosphate synthetase subunit Vtc2/3 (predicted)	0.51	3.53	9.55E-04	2.29E-02
SPBC1709.12	rid1	GTPase binding protein Rid1 (predicted)	0.26	3.52	9.83E-04	2.29E-02
SPBC21D10.07	cmc1	copper-binding protein of the mitochondrial intermembrane space Cmc1 (predicted)	0.35	3.52	9.87E-04	2.29E-02
SPBC839.07	ibp1	Cdc25 family phosphatase Ibp1, unknown role, implicated in DNA replication	0.69	3.52	9.75E-04	2.29E-02
SPCC18.09c	hnt3	aprataxin Hnt3	0.44	3.51	1.01E-03	2.32E-02
SPCC1827.07c	SPCC1827.07c	SPX/EXS domain protein (predicted)	0.2	3.49	1.05E-03	2.40E-02
SPBC337.11	SPBC337.11	mitochondrial inner membrane CH-OH group oxidoreductase family, reticulon interacting protein, implicated in mitochondrial organization or tethering	0.39	3.48	1.11E-03	2.46E-02
SPAC16E8.14c	tae1	ribosomal protein AdoMet-dependent proline dimethyltransferase Tae1 (predicted)	0.27	3.44	1.22E-03	2.57E-02
SPBC1683.12	SPBC1683.12	carboxylic acid transmembrane transporter (predicted)	0.38	3.43	1.29E-03	2.57E-02
SPBC1921.03c	mex67	mRNA export receptor, Tap, nucleoporin Mex67	0.2	3.45	1.21E-03	2.57E-02
SPBC25B2.04c	mtg1	mitochondrial translation factor (GTPase) Mtg1 (predicted)	0.19	3.43	1.28E-03	2.57E-02
SPBC713.07c	SPBC713.07c	vacuolar polyphosphatase (predicted)	0.28	3.43	1.28E-03	2.57E-02
SPBC83.19c	SPBC83.19c	Schizosaccharomyces pombe specific protein	0.23	3.47	1.22E-03	2.57E-02
SPAPB8E5.04c	npc2	Niemann-Pick disease type C2 protein hE1 homolog Npc2 (predicted)	0.24	3.41	1.36E-03	2.60E-02
SPBC800.12c	SPBC800.12c	ubiquitin family protein (predicted)	0.28	3.41	1.36E-03	2.60E-02
SPBPB2B2.09c	pan5	2-dehydropantoate 2-reductase Pan5 (predicted)	0.49	3.39	1.44E-03	2.70E-02
SPAC890.07c	rmt1	type I protein arginine N-methyltransferase Rmt1	0.31	3.35	1.72E-03	3.08E-02
SPCC548.05c	dbl5	ubiquitin-protein ligase E3 Dbl5	0.34	3.33	1.71E-03	3.08E-02
SPCC18B5.11c	cds1	replication checkpoint kinase Cds1	0.37	3.31	1.82E-03	3.16E-02
SPAC18B11.08c	SPAC18B11.08c	UPF0139 family conserved fungal ER membrane protein	0.3	3.28	1.95E-03	3.34E-02
SPAC1751.01c	gti1	gluconate transmembrane transporter inducer Gti1	0.46	3.26	2.11E-03	3.51E-02
SPBC342.03	gas4	spore wall 1,3-beta-glucanosyltransferase Gas4	0.21	3.25	2.12E-03	3.51E-02
SPCC736.09c	tfx1	TRAX	0.23	3.24	2.19E-03	3.58E-02
SPBC83.05	SPBC83.05	mitochondrial RNA-binding protein (predicted)	0.64	3.22	2.31E-03	3.70E-02
SPAC17G6.15c	fsf1	mitochondrial carrier, serine Fsf1 (predicted)	0.2	3.2	2.45E-03	3.86E-02
SPBC11C11.06c	SPBC11C11.06c	Schizosaccharomyces specific protein	0.25	3.2	2.49E-03	3.86E-02
SPAC13A11.05	ysp2	peptidase family M17 cytoplasmic leucyl aminopeptidase yspII (LAP yspII)	0.18	3.17	2.69E-03	4.11E-02
SPBC16A3.14	SPBC16A3.14	superoxide dismutase, mitochondrial ribosomal protein subunit (predicted)	0.2	3.15	2.84E-03	4.28E-02
SPBC691.03c	apl3	AP-2 adaptor complex alpha subunit Alp3	0.34	3.15	2.85E-03	4.28E-02
SPBC651.03c	gyp10	GTPase activating protein Gyp10	0.23	3.14	2.92E-03	4.36E-02
SPBC1773.16c	SPBC1773.16c	transcription factor, zf-fungal binuclear cluster type(predicted)	0.27	3.13	3.00E-03	4.44E-02
SPAC17A2.14	mnr2	vacuolar CorA family magnesium ion transmembrane transporter Mnr2	0.25	3.14	3.06E-03	4.45E-02
SPBC8D2.19	mde3	serine/threonine protein kinase, meiotic Mde3	0.69	3.12	3.06E-03	4.45E-02
SPBC365.11	grp2	Golgi GRIP domain protein Grp2 (predicted)	0.2	3.11	3.17E-03	4.53E-02
SPAC56F8.14c	mug115	Schizosaccharomyces pombe specific protein Mug115	0.36	3.08	3.51E-03	4.86E-02
SPBC8D2.18c	SPBC8D2.18c	adenosylhomocysteinase (predicted)	0.22	3.07	3.59E-03	4.94E-02
SPBC21C3.09c	oaa1	mitochondrial acylpyruvase Oaa1 (predicted)	0.32	3.06	3.71E-03	4.97E-02
SPBC28E12.06c	lvs1	beige protein homolog Lvs1	0.27	3.06	3.71E-03	4.97E-02
SPBC29A3.21	SPBC29A3.21	Schizosaccharomyces pombe specific protein	1.39	9.03	1.84E-11	4.78E-08
SPBC211.06	gfh1	gamma tubulin complex subunit Gfh1	0.48	6.24	1.18E-07	1.02E-04
SPBC32F12.09	rum1	CDK inhibitor Rum1	0.42	5.61	1.07E-06	5.22E-04
SPBC418.01c	his4	imidazoleglycerol-phosphate synthase His4	0.46	5.42	2.04E-06	5.60E-04
SPBC1271.09	tgp1	plasma membrane glycerophosphodiester transmembrane transporter (predicted)	0.36	5.26	3.50E-06	7.58E-04
SPCPB1C11.02	SPCPB1C11.02	amino acid transmembrane transporter (predicted)	0.35	4.97	9.43E-06	1.53E-03
SPAC18B11.04	ncs1	neuronal calcium sensor related protein Ncs1	0.34	4.86	1.37E-05	1.85E-03
SPBC4.06	SPBC4.06	acid phosphatase Fmp10 (predicted)	0.34	4.86	1.36E-05	1.85E-03
SPBC839.11c	hut1	ER uridine diphosphate-glucose transmembrane transporter Hut1	0.36	4.79	1.73E-05	2.04E-03
SPAC222.07c	hri2	eIF2 alpha kinase Hri2	0.42	4.75	2.00E-05	2.17E-03

SPBC3B9.06c	atg3	autophagy associated protein Atg3	0.47	4.57	3.63E-05	2.65E-03
SPBC405.05	atg16	autophagy associated protein Atg16	0.3	4.59	3.34E-05	2.65E-03
SPBC8D2.01	gsk31	serine/threonine protein kinase Gsk31 (predicted)	0.59	4.58	3.46E-05	2.65E-03
SPAC11D3.04c	SPAC11D3.04c	polyketide cyclase SnoaL-like domain protein	0.61	4.47	4.92E-05	3.46E-03
SPBP8B7.24c	atg8	autophagy associated protein Atg8	0.34	4.42	5.84E-05	3.89E-03
SPBP26C9.03c	fet4	plasma membrane iron/zinc ion transmembrane transporter (predicted)	0.32	4.37	6.82E-05	4.33E-03
SPBC16E9.17c	rem1	meiosis-specific cyclin Rem1	0.33	4.35	7.26E-05	4.50E-03
SPBC12C2.12c	glo1	glyoxalase I	0.29	4.22	1.12E-04	5.88E-03
SPAC3A11.05c	kms1	meiotic spindle pole body KASH domain protein Kms1	0.33	4.15	1.41E-04	6.85E-03
SPAC23C4.12	hhp2	serine/threonine protein kinase Hhp2	0.36	4.11	1.58E-04	7.32E-03
SPBC16E9.11c	pub3	HECT-type ubiquitin-protein ligase E3 Pub3 (predicted)	0.29	3.99	2.35E-04	9.85E-03
SPBC29A3.18	cyt1	cytochrome c1 Cyt1 (predicted)	0.25	3.94	2.75E-04	1.12E-02
SPAC57A7.09	SPAC57A7.09	ubiquitin-protein ligase E3, human RNF13 family homolog, unknown biological role	0.26	3.93	2.84E-04	1.13E-02
SPBC16E9.02c	SPBC16E9.02c	CUE domain protein, human TOLLIP ortholog	0.33	3.88	3.28E-04	1.24E-02
SPBC18E5.07	aim21	barbed end F-actin assembly inhibitor	0.35	3.86	3.47E-04	1.27E-02
SPBC6B1.02	ppk30	Ark1/Prk1 family protein kinase Ppk30	0.9	3.84	3.68E-04	1.29E-02
SPAC1039.03	SPAC1039.03	esterase/lipase, implicated in cellular detoxification (predicted)	0.24	3.8	4.15E-04	1.37E-02
SPAC1952.09c	SPAC1952.09c	acetyl-CoA hydrolase (predicted)	0.27	3.79	4.31E-04	1.37E-02
SPBC354.15	fap1	L-pipecolate oxidase	0.3	3.79	4.32E-04	1.37E-02
SPCC70.08c	SPCC70.08c	methyltransferase (predicted)	0.55	3.78	4.39E-04	1.38E-02
SPBC19C2.02	pmt1	tRNA (cytosine-5-)-methyltransferase Pmt1	0.27	3.74	5.01E-04	1.53E-02
SPAC1F7.12	yak3	aldose reductase ARK13 family YakC, implicated in cellular detoxification from family members	0.33	3.69	5.85E-04	1.73E-02
SPCC757.11c	SPCC757.11c	transmembrane transporter (predicted)	0.31	3.67	6.19E-04	1.79E-02
SPBC609.02	ptn1	phosphatidylinositol-3,4,5-trisphosphate3-phosphatase Ptn1	0.71	3.64	6.73E-04	1.86E-02
SPAC22G7.02	kap111	karyopherin/importin beta family nuclear import signal receptor Kap111 (predicted)	0.33	3.66	7.00E-04	1.91E-02
SPBC1198.07c	SPBC1198.07c	mannan endo-1,6-alpha-mannosidase (predicted)	0.24	3.58	8.22E-04	2.10E-02
SPAC1420.01c	SPAC1420.01c	GATA-like domain protein (predicted)	0.48	3.56	8.60E-04	2.17E-02
SPBC23G7.06c	nvj2	nucleus-vacuole junction protein Nvj2	0.27	3.54	9.32E-04	2.29E-02
SPAC14C4.11	vtc2	vacuolar transporter chaperone (VTC) complex polyphosphate synthetase subunit Vtc2/3 (predicted)	0.51	3.53	9.55E-04	2.29E-02
SPBC1709.12	rid1	GTPase binding protein Rid1 (predicted)	0.26	3.52	9.83E-04	2.29E-02
SPBC21D10.07	cmc1	copper-binding protein of the mitochondrial intermembrane space Cmc1 (predicted)	0.35	3.52	9.87E-04	2.29E-02
SPBC839.07	ibp1	Cdc25 family phosphatase Ibp1, unknown role, implicated in DNA replication	0.69	3.52	9.75E-04	2.29E-02
SPCC18.09c	hnt3	aprataxin Hnt3	0.44	3.51	1.01E-03	2.32E-02
SPCC1827.07c	SPCC1827.07c	SPX/EXS domain protein (predicted)	0.2	3.49	1.05E-03	2.40E-02
SPBC337.11	SPBC337.11	mitochondrial inner membrane CH-OH group oxidoreductase family, reticulon interacting protein, implicated in mitochondrial organization or tethering	0.39	3.48	1.11E-03	2.46E-02
SPAC16E8.14c	tae1	ribosomal protein AdoMet-dependent proline dimethyltransferase Tae1 (predicted)	0.27	3.44	1.22E-03	2.57E-02
SPBC1683.12	SPBC1683.12	carboxylic acid transmembrane transporter (predicted)	0.38	3.43	1.29E-03	2.57E-02
SPBC1921.03c	mex67	mRNA export receptor, Tap, nucleoporin Mex67	0.2	3.45	1.21E-03	2.57E-02
SPBC25B2.04c	mtg1	mitochondrial translation factor (GTPase) Mtg1 (predicted)	0.19	3.43	1.28E-03	2.57E-02
SPBC713.07c	SPBC713.07c	vacuolar polyphosphatase (predicted)	0.28	3.43	1.28E-03	2.57E-02
SPBC83.19c	SPBC83.19c	Schizosaccharomyces pombe specific protein	0.23	3.47	1.22E-03	2.57E-02
SPAPB8E5.04c	npc2	Niemann-Pick disease type C2 protein hE1 homolog Npc2 (predicted)	0.24	3.41	1.36E-03	2.60E-02
SPBC800.12c	SPBC800.12c	ubiquitin family protein (predicted)	0.28	3.41	1.36E-03	2.60E-02
SPBPB2B2.09c	pan5	2-dehydropantoate 2-reductase Pan5 (predicted)	0.49	3.39	1.44E-03	2.70E-02
SPAC890.07c	rmt1	type I protein arginine N-methyltransferase Rmt1	0.31	3.35	1.72E-03	3.08E-02
SPCC548.05c	dbl5	ubiquitin-protein ligase E3 Dbl5	0.34	3.33	1.71E-03	3.08E-02
SPCC18B5.11c	cds1	replication checkpoint kinase Cds1	0.37	3.31	1.82E-03	3.16E-02
SPAC18B11.08c	SPAC18B11.08c	UPF0139 family conserved fungal ER membrane protein	0.3	3.28	1.95E-03	3.34E-02
SPAC1751.01c	gti1	gluconate transmembrane transporter inducer Gti1	0.46	3.26	2.11E-03	3.51E-02
SPBC342.03	gas4	spore wall 1,3-beta-glucanosyltransferase Gas4	0.21	3.25	2.12E-03	3.51E-02
SPCC736.09c	tfx1	TRAX	0.23	3.24	2.19E-03	3.58E-02
SPBC83.05	SPBC83.05	mitochondrial RNA-binding protein (predicted)	0.64	3.22	2.31E-03	3.70E-02
SPAC17G6.15c	fsf1	mitochondrial carrier, serine Fsf1 (predicted)	0.2	3.2	2.45E-03	3.86E-02
SPBC11C11.06c	SPBC11C11.06c	Schizosaccharomyces specific protein	0.25	3.2	2.49E-03	3.86E-02

SPAC13A11.05	ysp2	peptidase family M17 cytoplasmic leucyl aminopeptidase yspII (LAP yspII)	0.18	3.17	2.69E-03	4.11E-02
SPBC16A3.14	SPBC16A3.14	superoxide dismutase, mitochondrial ribosomal protein subunit (predicted)	0.2	3.15	2.84E-03	4.28E-02
SPBC691.03c	apl3	AP-2 adaptor complex alpha subunit Alp3	0.34	3.15	2.85E-03	4.28E-02
SPBC651.03c	gyp10	GTPase activating protein Gyp10	0.23	3.14	2.92E-03	4.36E-02
SPBC1773.16c	SPBC1773.16c	transcription factor, zf-fungal binuclear cluster type(predicted)	0.27	3.13	3.00E-03	4.44E-02
SPAC17A2.14	mnr2	vacuolar CorA family magnesium ion transmembrane transporter Mnr2	0.25	3.14	3.06E-03	4.45E-02
SPBC8D2.19	mde3	serine/threonine protein kinase, meiotic Mde3	0.69	3.12	3.06E-03	4.45E-02
SPBC365.11	grp2	Golgi GRIP domain protein Grp2 (predicted)	0.2	3.11	3.17E-03	4.53E-02
SPAC56F8.14c	mug115	Schizosaccharomyces pombe specific protein Mug115	0.36	3.08	3.51E-03	4.86E-02
SPBC8D2.18c	SPBC8D2.18c	adenosylhomocysteinase (predicted)	0.22	3.07	3.59E-03	4.94E-02
SPBC21C3.09c	oaa1	mitochondrial acylpyruvase Oaa1 (predicted)	0.32	3.06	3.71E-03	4.97E-02
SPBC28E12.06c	lvs1	beige protein homolog Lvs1	0.27	3.06	3.71E-03	4.97E-02

Supplementary table4: Negative colony size difference between *btn1(D363G)* vs *btn1(102-208del)*

Systematic ID	Gene name	Product description	Colony Size Difference	t	P Value	Adjusted P Value
SPAPB2B4.02	grx5	mitochondrial [2Fe-2S] cluster assembly and transfer glutaredoxin Grx5	-0.42	-6.23	1.73E-07	1.52E-04
SPBP8B7.05c	nce103	carbonic anhydrase (predicted)	-0.62	-6.13	1.75E-07	1.52E-04
SPCC16C4.10	SPCC16C4.10	6-phosphogluconolactonase (predicted)	-0.35	-5.67	8.73E-07	4.54E-04
SPBC887.10	mcs4	response regulator Mcs4	-0.86	-5.56	1.28E-06	5.56E-04
SPCC1393.08	fil1	transcription factor, zf-GATA type	-0.46	-5.38	2.36E-06	7.65E-04
SPBC12C2.04	SPBC12C2.04	NAD binding dehydrogenase family protein	-0.41	-5.3	3.09E-06	8.03E-04
SPBC1604.03c	SPBC1604.03c	conserved fungal protein, implicated in vesicle trafficking or lipid metabolism	-0.34	-5.29	3.20E-06	8.03E-04
SPAC31A2.11c	cuf1	nutritional copper sensing transcription factor Cuf1	-0.38	-5.2	4.32E-06	9.30E-04
SPAC823.10c	hem25	mitochondrial carrier, glycine Hem25 (predicted)	-0.53	-5.18	4.65E-06	9.30E-04
SPBC947.08c	hip4	histone promoter control protein Hip4	-0.58	-5.07	6.68E-06	1.24E-03
SPBC1105.10	rav1	RAVE complex subunit Rav1	-0.34	-5.02	8.05E-06	1.40E-03
SPBC19F8.08	rps401	40S ribosomal protein S4 (predicted)	-0.41	-4.98	9.25E-06	1.41E-03
SPBC1D7.03	clg1	cyclin-like protein involved in autophagy Clg1 (predicted)	-0.34	-4.96	9.77E-06	1.41E-03
SPAC13G6.15c	SPAC13G6.15c	calcipressin (predicted)	-0.5	-4.78	1.80E-05	1.87E-03
SPBC4B4.03	rsc1	RSC complex subunit Rsc1	-0.48	-4.82	1.58E-05	1.87E-03
SPBC543.07	pek1	MAP kinase kinase Pek1	-0.38	-4.79	1.71E-05	1.87E-03
SPCC18B5.10c	tex1	TREX complex subunit Tex1 (predicted)	-0.58	-4.78	1.77E-05	1.87E-03
SPBC2D10.13	est1	telomerase regulator Est1	-0.4	-4.75	1.97E-05	1.97E-03
SPBC19C2.13c	ctu2	cytosolic thiouridylase subunit Ctu2	-0.34	-4.69	2.37E-05	2.20E-03
SPBC530.08	SPBC530.08	membrane-tethered transcription factor (predicted)	-0.35	-4.7	2.35E-05	2.20E-03
SPAC13G6.14	aps1	diadenosine 5',5'''-p1,p6-hexaphosphate hydrolase Aps1	-0.85	-4.57	3.62E-05	3.03E-03
SPCC1739.10	mug33	Tea1-interacting protein involved in exocytosis	-0.49	-4.54	3.98E-05	3.24E-03
SPAC26H5.10c	tif51	translation elongation and termination factor eIF5A (predicted)	-0.4	-4.5	4.53E-05	3.57E-03
SPAC19B12.10	sst2	human AMSH/STAMBP protein homolog, ubiquitin specific-protease	-0.61	-4.49	5.30E-05	3.94E-03
SPBC3H7.03c	kgd1	2-oxoglutarate dehydrogenase (lipoamide) (e1 component of oxoglutarate dehydrogenase complex) (predicted)	-0.44	-4.45	5.32E-05	3.94E-03
SPBP16F5.05c	yar1	ribosome biogenesis protein Yar1 (predicted)	-0.41	-4.44	5.45E-05	3.94E-03
SPAC144.03	ade2	adenylosuccinate synthetase Ade2	-0.47	-4.41	6.08E-05	4.05E-03
SPBC887.15c	sur2	sphingosine hydroxylase/sphingolipid delta-4 desaturase activity Sur2	-0.36	-4.42	5.81E-05	4.05E-03
SPAC3G9.03	rpl2301	60S ribosomal protein L23	-0.34	-4.4	6.29E-05	4.09E-03
SPBC3E7.01	fab1	1-phosphatidylinositol-3-phosphate 5-kinase Fab1	-0.51	-4.36	7.09E-05	4.49E-03
SPBC17A3.09c	aim22	lipoate-protein ligase A (predicted)	-0.44	-4.27	9.41E-05	5.46E-03
SPBC29A10.08	gas2	cell wall 1,3-beta-glucanosyltransferase Gas2	-0.34	-4.27	9.45E-05	5.46E-03
SPCC126.04c	sgf73	SAGA complex deubiquitinating submodule subunit Sgf73	-0.42	-4.28	9.20E-05	5.46E-03
SPBC530.06c	clu1	clustered mitochndria ortholog Clu1	-0.5	-4.24	1.04E-04	5.87E-03
SPBC23E6.01c	cxr1	splicing factor Cxr1	-0.37	-4.23	1.08E-04	5.92E-03
SPAC1B3.07c	vps28	ESCRT I complex subunit Vps28	-0.3	-4.18	1.26E-04	6.07E-03
SPBC119.06	sco1	mitochondrial copper chaperone for cytochrome c oxidase Sco1 (predicted)	-0.35	-4.19	1.24E-04	6.07E-03
SPBC1A4.04	SPBC1A4.04	Schizosaccharomyces specific protein	-0.37	-4.19	1.22E-04	6.07E-03
SPBC3B9.09	vps36	ESCRT II complex subunit Vps36	-0.24	-4.19	1.23E-04	6.07E-03

SPAC31G5.04	lys12	homoisocitrate dehydrogenase Lys12	-0.28	-4.15	1.39E-04	6.30E-03
SPBC25H2.16c	gga22	Golgi localized Arf binding gamma-adaptin ortholog Gga22	-0.29	-4.14	1.45E-04	6.30E-03
SPBC409.07c	wis1	MAP kinase kinase Wis1	-0.78	-4.14	1.45E-04	6.30E-03
SPBC543.09	yta12	mitochondrial m-AAA protease Yta12 (predicted)	-0.4	-4.15	1.38E-04	6.30E-03
SPBC8D2.03c	hhf2	histone H4 h4.2	-0.31	-4.14	1.42E-04	6.30E-03
SPBC685.06	rps001	40S ribosomal protein S0A (p40)	-0.33	-4.07	1.81E-04	7.57E-03
SPAC19G12.13c	poz1	shelterin complex subunit Poz1	-0.48	-4	2.22E-04	8.97E-03
SPBC21C3.02c	dep1	Sds3-like family protein Dep1	-0.28	-3.99	2.29E-04	9.00E-03
SPBC21C3.08c	car2	ornithine transaminase Car2	-0.7	-3.98	2.37E-04	9.21E-03
SPAC8F11.02c	dph3	diphthamide biosynthesis protein Dph3 (predicted)	-0.54	-3.96	2.57E-04	9.55E-03
SPBC21B10.03c	ath1	ataxin-2 homolog	-0.41	-3.96	2.54E-04	9.55E-03
SPBC3H7.15	hhp1	serine/threonine protein kinase Hhp1	-0.26	-3.96	2.55E-04	9.55E-03
SPBC16G5.15c	fkh2	forkhead transcription factor Fkh2	-0.39	-3.95	2.66E-04	9.57E-03
SPBC365.10	arp5	Ino80 complex actin-like protein Arp5	-0.37	-3.94	2.69E-04	9.57E-03
SPCC285.17	spp27	RNA polymerase I upstream activation factor complex subunit Spp27	-0.59	-3.9	3.10E-04	1.06E-02
SPBC106.10	pka1	cAMP-dependent protein kinase catalytic subunit Pka1	-0.28	-3.87	3.41E-04	1.15E-02
SPBC3D6.04c	mad1	mitotic spindle checkpoint protein Mad1	-0.36	-3.83	3.86E-04	1.27E-02
SPBC1778.03c	SPBC1778.03c	NADH pyrophosphatase (predicted)	-0.32	-3.82	3.92E-04	1.27E-02
SPBC1604.02c	ppr1	mitochondrial PPR repeat protein Ppr1	-0.32	-3.79	4.27E-04	1.36E-02
SPBC2F12.11c	rep2	MBF transcription factor activator Rep2	-0.59	-3.79	4.28E-04	1.36E-02
SPAC8E11.07c	alp31	tubulin specific chaperone cofactor A, Alp31	-0.34	-3.79	4.62E-04	1.38E-02
SPBC16E9.16c	lsd90	Lsd90 protein	-0.51	-3.77	4.52E-04	1.38E-02
SPCC594.05c	spf1	Set1C PHD finger subunit Spf1	-0.28	-3.76	4.78E-04	1.41E-02
SPAC1A6.04c	plb1	phospholipase B homolog Plb1	-0.47	-3.72	5.31E-04	1.55E-02
SPBC409.19c	mtx2	metaxin 2 Mtx2 (predicted)	-0.45	-3.7	5.74E-04	1.64E-02
SPBC1A4.05	blt1	ubiquitin domain-like protein Blt1	-0.38	-3.68	6.01E-04	1.68E-02
SPAC1071.04c	spc2	signal peptidase subunit Spc2 (predicted)	-0.31	-3.67	6.18E-04	1.71E-02
SPAC22F3.08c	rok1	ATP-dependent RNA helicase Rok1 (predicted)	-0.55	-3.69	6.39E-04	1.71E-02
SPAC6F12.02	rst2	transcription factor Rst2	-0.44	-3.66	6.37E-04	1.71E-02
SPBC14F5.09c	ade8	adenylosuccinate lyase Ade8	-0.4	-3.67	6.27E-04	1.71E-02
SPAC22F8.02c	pvg5	Golgi 4,6-pyruvylated galactose (PvGal) residue biosynthesis protein Pvg5	-0.31	-3.61	7.49E-04	1.86E-02
SPCC757.05c	SPCC757.05c	peptidase family M20 protein involved in glutathione catabolism (predicted)	-0.34	-3.57	8.28E-04	1.97E-02
SPAC4H3.13	pcc1	EKC/KEOPS complex subunit Pcc1 (predicted)	-0.55	-3.57	8.43E-04	1.99E-02
SPAC14C4.14	atp1	F1-FO ATP synthase alpha subunit	-0.48	-3.53	9.55E-04	2.11E-02
SPAC890.05	pxr1	ribosome biogenesis protein, G-patch domain, PINX1 family Pxr1 (predicted)	-0.42	-3.53	9.59E-04	2.11E-02
SPBC11G11.01	fis1	mitochondrial fission protein Fis1 (predicted)	-0.31	-3.52	9.82E-04	2.11E-02
SPBC27B12.10c	tom7	mitochondrial TOM complex subunit Tom7 (predicted)	-0.24	-3.53	9.54E-04	2.11E-02
SPBC557.02c	SPBC557.02c	DUF2458 conserved fungal protein	-0.27	-3.54	9.19E-04	2.11E-02
SPBC577.02	rpl3801	60S ribosomal protein L38 (predicted)	-0.31	-3.49	1.13E-03	2.33E-02
SPAC8E11.05c	SPAC8E11.05c	DUF5102 family conserved fungal protein, associated with clathrin coated vesicles (predicted)	-0.31	-3.44	1.24E-03	2.50E-02
SPBC119.12	rud3	Golgi matrix protein Rud3 (predicted)	-0.35	-3.43	1.28E-03	2.50E-02
SPBC409.11	meu18	Schizosaccharomyces specific protein Meu18	-0.27	-3.43	1.28E-03	2.50E-02
SPCC794.11c	ent3	ENTH/VHS domain protein Ent3 (predicted)	-0.3	-3.44	1.25E-03	2.50E-02
SPCC1739.14	npp106	nucleoporin Npp106	-0.27	-3.39	1.42E-03	2.75E-02
SPCP1E11.05c	are2	acyl-coA-sterol acyltransferase Are2	-0.23	-3.39	1.43E-03	2.75E-02
SPBC27B12.03c	erg32	C-5 sterol desaturase Erg32	-0.36	-3.36	1.56E-03	2.93E-02
SPAC30.02c	kti12	elongator complex associated protein Kti12 (predicted)	-0.36	-3.37	1.60E-03	2.96E-02
SPBCPT2R1.08c	tlh2	RecQ type DNA helicase Tlh1	-0.3	-3.37	1.60E-03	2.96E-02
SPBC428.08c	clr4	histone lysine H3 methyltransferase Clr4	-0.32	-3.35	1.62E-03	2.97E-02
SPCC16C4.20c	hap2	Ino80 complex, HMG box protein Hap2	-0.39	-3.35	1.62E-03	2.97E-02
SPCC548.06c	ght8	plasma membrane hexose:proton symporter, unknown specificity Ght8 (predicted)	-0.31	-3.34	1.65E-03	2.98E-02
SPAC4G8.10	gos1	SNARE Gos1 (predicted)	-0.28	-3.33	1.69E-03	3.01E-02
SPBC887.05c	cwf29	RNA-binding protein Cwf29	-0.4	-3.33	1.69E-03	3.01E-02
SPAC1071.07c	rps1502	40S ribosomal protein S15 (predicted)	-0.3	-3.32	1.76E-03	3.10E-02

SPBC3E7.10	fma1	methionine aminopeptidase Fma1 (predicted)	-0.31	-3.32	1.76E-03	3.10E-02
SPAC1071.02	mms19	CIA machinery protein Mms19	-0.82	-3.31	1.89E-03	3.30E-02
SPAC16C9.05	cph1	Clr6 histone deacetylase associated PHD finger protein Cph1	-0.46	-3.3	1.93E-03	3.35E-02
SPBC839.15c	tef103	translation elongation factor EF-1 alpha Ef1a-c	-0.44	-3.25	2.11E-03	3.56E-02
SPCC1919.03c	amk2	AMP-activated protein kinase beta subunit Amk2	-0.27	-3.26	2.08E-03	3.56E-02
SPBC1718.03	ker1	DNA-directed RNA polymerase I complex subunit Ker1	-0.25	-3.24	2.20E-03	3.64E-02
SPAC8E11.02c	rad24	14-3-3 protein Rad24	-0.25	-3.22	2.33E-03	3.84E-02
SPBC31F10.14c	hip3	HIRA interacting protein Hip3	-0.58	-3.21	2.42E-03	3.89E-02
SPBP16F5.03c	tra1	SAGA complex phosphatidylinositol pseudokinase Tra1	-0.39	-3.21	2.41E-03	3.89E-02
SPAC2F7.07c	cph2	Clr6 histone deacetylase associated PHD-finger protein Cph2	-0.33	-3.2	2.46E-03	3.93E-02
SPAC30C2.06c	dml1	mitochondrial inheritance GTPase, tubulin-like (predicted)	-0.41	-3.19	2.57E-03	4.03E-02
SPBC21C3.20c	git1	C2 domain protein Git1	-0.2	-3.18	2.63E-03	4.05E-02
SPCC794.08	efr3	phosphatidylinositol-4 kinase plasma membrane scaffold Efr3	-0.29	-3.18	2.64E-03	4.05E-02
SPCC790.02	pep3	HOPS/CORVET complex subunit, ubiquitin-protein ligase E3 Pep3/Vps18 (predicted)	-0.43	-3.17	2.69E-03	4.11E-02
SPAC16E8.05c	SPAC16E8.05c	Schizosaccharomyces specific protein Mde1	-0.33	-3.16	2.77E-03	4.18E-02
SPBC776.06c	SPBC776.06c	Arf3/6 docking factor (predicted)	-0.42	-3.16	2.76E-03	4.18E-02
SPBC1703.13c	SPBC1703.13c	mitochondrial carrier, inorganic phosphate (predicted)	-0.16	-3.16	2.79E-03	4.20E-02
SPAC23D3.09	arp42	SWI/SNF and RSC complex subunit Arp42	-0.39	-3.17	2.83E-03	4.23E-02
SPAC644.06c	cdr1	NIM1 family serine/threonine protein kinase Cdr1/Nim1	-0.64	-3.15	2.86E-03	4.23E-02
SPAC6F6.12	atg24	autophagy associated PX/BAR domain sorting nexin Atg24	-0.37	-3.15	2.88E-03	4.23E-02
SPBC21B10.10	rps402	40S ribosomal protein S4 (predicted)	-0.27	-3.13	2.97E-03	4.30E-02
SPBC1198.11c	reb1	RNA polymerase I transcription termination factor/ RNA polymerase II transcription factor Reb1	-0.35	-3.12	3.12E-03	4.46E-02
SPBC30D10.10c	tor1	serine/threonine protein kinase Tor1	-0.48	-3.1	3.23E-03	4.55E-02
SPBC32F12.01c	css1	inositol phosphosphingolipid phospholipase C, Css1	-0.33	-3.1	3.23E-03	4.55E-02
SPBC21C3.07c	trm140	tRNA (cytosine-3) methyltransferase Trm140 (predicted)	-0.54	-3.09	3.37E-03	4.66E-02
SPCC1223.05c	rpl3702	60S ribosomal protein L37 (predicted)	-0.3	-3.09	3.37E-03	4.66E-02
SPAC212.03	SPAC212.03	hypothetical protein	-0.44	-3.1	3.46E-03	4.71E-02
SPBC1709.04c	cyp3	cyclophilin family peptidyl-prolyl cis-trans isomerase Cyp3	-0.19	-3.08	3.43E-03	4.71E-02
SPAC29A4.20	elp3	elongator complex subunit Elp3	-0.24	-3.07	3.52E-03	4.77E-02
SPAC13C5.06c	mug121	Schizosaccharomyces pombe specific protein Mug121	-0.24	-3.07	3.56E-03	4.77E-02
SPBC106.16	pre6	20S proteasome complex subunit alpha 4 Pre6	-0.5	-3.07	3.56E-03	4.77E-02
SPAC1687.23c	SPAC1687.23c	Schizosaccharomyces pombe specific protein	-0.3	-3.07	3.72E-03	4.94E-02

Supplementary table5: Positive colony size difference between *btn1(D363G)* vs *btn1(102-208del)*

Systematic ID	Gene name	Product description	Colony Size Difference	t	P Value	Adjusted P Value
SPBC29A3.21	SPBC29A3.21	Schizosaccharomyces pombe specific protein	1.49	9.84	1.52E-12	3.96E-09
SPBC211.06	gfh1	gamma tubulin complex subunit Gfh1	0.44	5.88	4.17E-07	2.71E-04
SPBC32F12.09	rum1	CDK inhibitor Rum1	0.4	5.41	2.14E-06	7.65E-04
SPAC18B11.04	ncs1	neuronal calcium sensor related protein Ncs1	0.37	5.27	3.40E-06	8.03E-04
SPBC418.01c	his4	imidazoleglycerol-phosphate synthase His4	0.41	4.98	9.00E-06	1.41E-03
SPBC839.11c	hut1	ER uridine diphosphate-glucose transmembrane transporter Hut1	0.36	4.87	1.30E-05	1.78E-03
SPBC12C2.12c	glo1	glyoxalase I	0.33	4.84	1.46E-05	1.87E-03
SPBC8D2.01	gsk31	serine/threonine protein kinase Gsk31 (predicted)	0.61	4.79	1.74E-05	1.87E-03
SPBC1271.09	tgp1	plasma membrane glycerophosphodiester transmembrane transporter (predicted)	0.32	4.67	2.59E-05	2.32E-03
SPCC757.11c	SPCC757.11c	transmembrane transporter (predicted)	0.38	4.62	3.02E-05	2.62E-03
SPBC651.03c	gyp10	GTPase activating protein Gyp10	0.32	4.41	6.03E-05	4.05E-03
SPBP26C9.03c	fet4	plasma membrane iron/zinc ion transmembrane transporter (predicted)	0.31	4.31	8.39E-05	5.19E-03
SPBC16E9.17c	rem1	meiosis-specific cyclin Rem1	0.32	4.23	1.09E-04	5.92E-03
SPAC222.07c	hri2	eIF2 alpha kinase Hri2	0.37	4.22	1.12E-04	5.93E-03
SPBC354.15	fap1	L-pipecolate oxidase	0.32	4.2	1.20E-04	6.07E-03
SPBP8B7.24c	atg8	autophagy associated protein Atg8	0.31	4.18	1.28E-04	6.07E-03
SPAC3A11.05c	kms1	meiotic spindle pole body KASH domain protein Kms1	0.32	4.12	1.52E-04	6.47E-03
SPAC13A11.05	ysp2	peptidase family M17 cytoplasmic leucyl aminopeptidase yspII (LAP yspII)	0.23	4	2.24E-04	8.97E-03
SPBC6B1.02	ppk30	Ark1/Prk1 family protein kinase Ppk30	0.92	4.01	2.19E-04	8.97E-03

SPBC1683.12	SPBC1683.12	carboxylic acid transmembrane transporter (predicted)	0.43	3.95	2.62E-04	9.57E-03
SPBC4.06	SPBC4.06	acid phosphatase Fmp10 (predicted)	0.27	3.93	2.77E-04	9.59E-03
SPCC18.09c	hnt3	aprataxin Hnt3	0.48	3.93	2.76E-04	9.59E-03
SPBC16E9.11c	pub3	HECT-type ubiquitin-protein ligase E3 Pub3 (predicted)	0.28	3.86	3.45E-04	1.15E-02
SPAC23C4.12	hhp2	serine/threonine protein kinase Hhp2	0.33	3.78	4.39E-04	1.38E-02
SPAC1952.09c	SPAC1952.09c	acetyl-CoA hydrolase (predicted)	0.27	3.77	4.59E-04	1.38E-02
SPCPB1C11.02	SPCPB1C11.02	amino acid transmembrane transporter (predicted)	0.26	3.77	4.60E-04	1.38E-02
SPBC405.05	atg16	autophagy associated protein Atg16	0.24	3.7	5.64E-04	1.63E-02
SPAC1F7.12	yak3	aldose reductase ARK13 family YakC, implicated in cellular detoxification from family members	0.32	3.69	5.81E-04	1.64E-02
SPBC1289.14	SPBC1289.14	adducin (predicted)	0.34	3.65	6.58E-04	1.73E-02
SPBC839.07	ibp1	Cdc25 family phosphatase Ibp1, unknown role, implicated in DNA replication	0.7	3.65	6.56E-04	1.73E-02
SPBC3B9.06c	atg3	autophagy associated protein Atg3	0.37	3.64	6.71E-04	1.74E-02
SPBC713.07c	SPBC713.07c	vacuolar polyphosphatase (predicted)	0.29	3.64	6.74E-04	1.74E-02
SPBC29A3.18	cyt1	cytochrome c1 Cyt1 (predicted)	0.22	3.63	7.01E-04	1.79E-02
SPBC902.05c	idh2	isocitrate dehydrogenase (NAD+) subunit 2	0.33	3.62	7.22E-04	1.82E-02
SPBC609.02	ptn1	phosphatidylinositol-3,4,5-trisphosphate3-phosphatase Ptn1	0.69	3.61	7.49E-04	1.86E-02
SPBC19C2.02	pmt1	tRNA (cytosine-5-)-methyltransferase Pmt1	0.25	3.6	7.62E-04	1.87E-02
SPBC12D12.05c	SPBC12D12.05c	mitochondrial carrier, ATP:ADP antiporter (predicted)	0.27	3.6	7.70E-04	1.87E-02
SPBC337.11	SPBC337.11	mitochondrial inner membrane CH-OH group oxidoreductase family, reticulon interacting protein, implicated in mitochondrial organization or tethering	0.39	3.59	7.80E-04	1.88E-02
SPAC1420.01c	SPAC1420.01c	GATA-like domain protein (predicted)	0.47	3.52	9.72E-04	2.11E-02
SPBC11C11.06c	SPBC11C11.06c	Schizosaccharomyces specific protein	0.27	3.53	9.47E-04	2.11E-02
SPBC18E5.07	aim21	barbed end F-actin assembly inhibitor	0.31	3.52	9.80E-04	2.11E-02
SPBC839.06	cta3	P-type ATPase, potassium exporting Cta3	0.41	3.53	9.38E-04	2.11E-02
SPCC548.05c	dbl5	ubiquitin-protein ligase E3 Dbl5	0.35	3.53	9.58E-04	2.11E-02
SPCC736.09c	tfx1	TRAX	0.25	3.53	9.56E-04	2.11E-02
SPAC1751.01c	gti1	gluconate transmembrane transporter inducer Gti1	0.49	3.51	1.01E-03	2.13E-02
SPBC1709.12	rid1	GTPase binding protein Rid1 (predicted)	0.25	3.51	1.01E-03	2.13E-02
SPBC776.04	sec2302	COPII cargo receptor subunit Sec23b (predicted)	0.27	3.51	1.00E-03	2.13E-02
SPAC17G6.15c	fsf1	mitochondrial carrier, serine Fsf1 (predicted)	0.22	3.49	1.06E-03	2.21E-02
SPAC57A7.09	SPAC57A7.09	ubiquitin-protein ligase E3, human RNF13 family homolog, unknown biological role	0.22	3.46	1.15E-03	2.36E-02
SPBC83.19c	SPBC83.19c	Schizosaccharomyces pombe specific protein	0.22	3.45	1.27E-03	2.50E-02
SPCC1827.07c	SPCC1827.07c	SPX/EXS domain protein (predicted)	0.19	3.44	1.24E-03	2.50E-02
SPBC2D10.04	aly2	arrestin Aly1 related Aly2	0.32	3.38	1.48E-03	2.82E-02
SPCC18B5.11c	cds1	replication checkpoint kinase Cds1	0.37	3.38	1.48E-03	2.82E-02
SPBC28E12.06c	lvs1	beige protein homolog Lvs1	0.29	3.34	1.63E-03	2.97E-02
SPBC16E9.03c	coa1	cytochrome c oxidase assembly protein Coa1 (predicted)	0.24	3.28	1.97E-03	3.39E-02
SPAC1F8.05	isp3	spore wall structural constituent Isp3	0.31	3.25	2.12E-03	3.56E-02
SPAC56F8.14c	mug115	Schizosaccharomyces pombe specific protein Mug115	0.37	3.25	2.12E-03	3.56E-02
SPAC6G9.13c	bqt1	bouquet formation protein Bqt1	0.23	3.24	2.18E-03	3.63E-02
SPAC16E8.14c	tae1	ribosomal protein AdoMet-dependent proline dimethyltransferase Tae1 (predicted)	0.25	3.21	2.42E-03	3.89E-02
SPBC83.05	SPBC83.05	mitochondrial RNA-binding protein (predicted)	0.62	3.21	2.42E-03	3.89E-02
SPAC18B11.08c	SPAC18B11.08c	UPF0139 family conserved fungal ER membrane protein	0.28	3.19	2.57E-03	4.03E-02
SPAC977.11	fex1	plasma membrane fluoride efflux channel Fex1	0.35	3.18	2.59E-03	4.03E-02
SPBC2G5.01	SPBC2G5.01	ER protein involved in ER-nucleus signaling (predicted)	0.36	3.18	2.59E-03	4.03E-02
SPBC31E1.01c	atg2	autophagy associated protein Atg2	0.3	3.15	2.88E-03	4.23E-02
SPAC890.07c	rmt1	type I protein arginine N-methyltransferase Rmt1	0.29	3.15	2.95E-03	4.30E-02
SPBC16A3.14	SPBC16A3.14	superoxide dismutase, mitochondrial ribosomal protein subunit (predicted)	0.2	3.13	2.97E-03	4.30E-02
SPAC22G7.02	kap111	karyopherin/importin beta family nuclear import signal receptor Kap111 (predicted)	0.28	3.14	3.04E-03	4.37E-02
SPBC16E9.02c	SPBC16E9.02c	CUE domain protein, human TOLLIP ortholog	0.26	3.11	3.18E-03	4.52E-02
SPAC8F11.08c	SPAC8F11.08c	ER membrane associated esterase/lipase (predicted)	0.24	3.1	3.26E-03	4.56E-02
SPBC887.17	SPBC887.17	nucleobase transmembrane transporter (predicted)	0.36	3.08	3.45E-03	4.71E-02
SPBC21D10.07	cmc1	copper-binding protein of the mitochondrial intermembrane space Cmc1 (predicted)	0.3	3.07	3.61E-03	4.81E-02
