# Supplementary material for: Global network analysis in Schizosaccharomyces pombe reveals three distinct consequences of the common 1-kb deletion causing juvenile CLN3 disease
Source: Sci Rep. 2021 Mar 18;11:6332. doi: 10.1038/s41598-021-85471-4 (PMC7973434; doi:10.1038/s41598-021-85471-4)
Supplement: Supplementary file 6 — S6: Supplementary Tables 6. [file 41598_2021_85471_MOESM6_ESM.pdf]

Global network analysis in *Schizosaccharomyces pombe* reveals three distinct consequences of the common 1-kb deletion causing juvenile CLN3 disease

Christopher J. Minnis[1,2], StJohn Townsend[3,4], Julia Petschnigg[1], Elisa Tinelli[1], Jürg Bähler[3], Claire Russell[2], Sara E. Mole[1]

[1]*MRC Laboratory for Molecular Cell Biology and Great Ormond Street Institute of Child Health, University College London, London WC1E 6BT, UK*
[2]*Dept. Comparative Biomedical Sciences, Royal Veterinary College, Royal College Street, London NW1 0TU, UK*
[3]*Institute of Healthy Ageing, Department of Genetics, Evolution and Environment, University College London, London WC1E 6BT, UK*
[4]*The Molecular Biology of Metabolism Laboratory, The Francis Crick Institute, London, NW1 1AT, United Kingdom*

*Corresponding author: christopher.minnis.15@ucl.ac.uk

Supplymentary table 1: Negative genetic interactions for *btn1*Δ vs *ade6*Δ control
None enriched

Supplymentary table 2: Positive genetic interactions for *btn1*Δ vs *ade6*Δ control

source	term_name	term_id	adjusted_p_value	negative_log10_of_adjusted_p_value	term_size	query_size	intersection_size	effective_domain_size	intersections
GO:MF	structural constituent of ribosome	GO:0003735	6.45163E-05	4.190330774	218	76	15	5032	SPAC3G9.03,SPAC144.11,SPBC19F8.08,SPBC839.13C,SPCC330.14C,SPBC577.02,SPCC1223.05C,SPCC285.15C,SPAC31G5.03,SPBC776.11,SPBC1921.01C,SPAC328.10C,SPAC644.15,SPCC1682.14,SPAC13G6.02C
GO:MF	structural molecule activity	GO:0005198	6.45163E-05	4.190330774	274	76	17	5032	SPAC3G9.03,SPAC1486.04C,SPAC144.11,SPBC19F8.08,SPBC839.13C,SPCC330.14C,SPBC577.02,SPCC1223.05C,SPCC285.15C,SPAC31G5.03,SPCC1739.14,SPBC776.11,SPBC1921.01C,SPAC328.10C,SPAC644.15,SPCC1682.14,SPAC13G6.02C
GO:MF	rRNA binding	GO:0019843	0.001783318	2.748771135	59	76	7	5032	SPAC3G9.03,SPAC144.11,SPBC19F8.08,SPCC1223.05C,SPAC31G5.03,SPBC1921.01C,SPAC328.10C
GO:BP	cytoplasmic translation	GO:0002181	4.61846E-05	4.335502832	269	76	18	5044	SPAC3G9.03,SPAC144.11,SPBC19F8.08,SPBC839.13C,SPAC323.05C,SPCC330.14C,SPBC577.02,SPCC1223.05C,SPCC285.15C,SPAC31G5.03,SPAC926.05C,SPBC776.11,SPBC1921.01C,SPAC328.10C,SPAC644.15,SPCC1682.14,SPCP31B10.07,SPAC13G6.02C
GO:BP	amide biosynthetic process	GO:0043604	0.015235401	1.8171461	485	76	19	5044	SPAC3G9.03,SPAC144.11,SPBC19F8.08,SPBC839.13C,SPAC323.05C,SPCC330.14C,SPAC144.04C,SPBC577.02,SPCC1223.05C,SPCC285.15C,SPAC31G5.03,SPAC926.05C,SPBC776.11,SPBC1921.01C,SPAC328.10C,SPAC644.15,SPCC1682.14,SPCP31B10.07,SPAC13G6.02C
GO:BP	peptide biosynthetic process	GO:0043043	0.015235401	1.8171461	438	76	18	5044	SPAC3G9.03,SPAC144.11,SPBC19F8.08,SPBC839.13C,SPAC323.05C,SPCC330.14C,SPBC577.02,SPCC1223.05C,SPCC285.15C,SPAC31G5.03,SPAC926.05C,SPBC776.11,SPBC1921.01C,SPAC328.10C,SPAC644.15,SPCC1682.14,SPCP31B10.07,SPAC13G6.02C
GO:BP	translation	GO:0006412	0.015235401	1.8171461	428	76	18	5044	SPAC3G9.03,SPAC144.11,SPBC19F8.08,SPBC839.13C,SPAC323.05C,SPCC330.14C,SPBC577.02,SPCC1223.05C,SPCC285.15C,SPAC31G5.03,SPAC926.05C,SPBC776.11,SPBC1921.01C,SPAC328.10C,SPAC644.15,SPCC1682.14,SPCP31B10.07,SPAC13G6.02C
GO:BP	peptide metabolic process	GO:0006518	0.019045007	1.720218874	458	76	18	5044	SPAC3G9.03,SPAC144.11,SPBC19F8.08,SPBC839.13C,SPAC323.05C,SPCC330.14C,SPBC577.02,SPCC1223.05C,SPCC285.15C,SPAC31G5.03,SPAC926.05C,SPBC776.11,SPBC1921.01C,SPAC328.10C,SPAC644.15,SPCC1682.14,SPCP31B10.07,SPAC13G6.02C
GO:BP	cellular amide metabolic process	GO:0043603	0.042621747	1.370368756	540	76	19	5044	SPAC3G9.03,SPAC144.11,SPBC19F8.08,SPBC839.13C,SPAC323.05C,SPCC330.14C,SPAC144.04C,SPBC577.02,SPCC1223.05C,SPCC285.15C,SPAC31G5.03,SPAC926.05C,SPBC776.11,SPBC1921.01C,SPAC328.10C,SPAC644.15,SPCC1682.14,SPCP31B10.07,SPAC13G6.02C

GO:BP	organonitrogen compound biosynthetic process	GO:1901566	0.08234746	1.084349794	873	76	25	5044	SPAC23A1.03,SPAC3G9.03,SPBC1734.12C,SPAC144.11,SPAC16.03C,SPAC1834.05,SPBC19F8.08,SPBC839.13C,SPAC323.05C,SPCC330.14C,SPAC144.04C,SPBC577.02,SPCC1223.05C,SPCC285.15C,SPAC31G5.03,SPAPB17E12.08,SPBC1734.13,SPAC926.05C,SPBC776.11,SPBC1921.01C,SPAC328.10C,SPAC644.15,SPCC1682.14,SPCP31B10.07,SPAC13G6.02C
GO:CC	cytosolic ribosome	GO:0022626	1.26911E-06	5.896500095	156	76	15	5075	SPAC3G9.03,SPAC144.11,SPBC19F8.08,SPBC839.13C,SPCC330.14C,SPBC577.02,SPCC1223.05C,SPCC285.15C,SPAC31G5.03,SPBC776.11,SPBC1921.01C,SPAC328.10C,SPAC644.15,SPCC1682.14,SPAC13G6.02C
GO:CC	ribosomal subunit	GO:0044391	9.62763E-05	4.016480663	227	76	15	5075	SPAC3G9.03,SPAC144.11,SPBC19F8.08,SPBC839.13C,SPCC330.14C,SPBC577.02,SPCC1223.05C,SPCC285.15C,SPAC31G5.03,SPBC776.11,SPBC1921.01C,SPAC328.10C,SPAC644.15,SPCC1682.14,SPAC13G6.02C
GO:CC	ribosome	GO:0005840	0.0001375	3.861698881	241	76	15	5075	SPAC3G9.03,SPAC144.11,SPBC19F8.08,SPBC839.13C,SPCC330.14C,SPBC577.02,SPCC1223.05C,SPCC285.15C,SPAC31G5.03,SPBC776.11,SPBC1921.01C,SPAC328.10C,SPAC644.15,SPCC1682.14,SPAC13G6.02C
GO:CC	cytosolic large ribosomal subunit	GO:0022625	0.000303623	3.51766498	89	76	9	5075	SPAC3G9.03,SPBC839.13C,SPCC330.14C,SPBC577.02,SPCC1223.05C,SPBC776.11,SPBC1921.01C,SPAC644.15,SPCC1682.14
GO:CC	large ribosomal subunit	GO:0015934	0.006869052	2.163103225	135	76	9	5075	SPAC3G9.03,SPBC839.13C,SPCC330.14C,SPBC577.02,SPCC1223.05C,SPBC776.11,SPBC1921.01C,SPAC644.15,SPCC1682.14
GO:CC	cytosolic small ribosomal subunit	GO:0022627	0.012302613	1.910002648	64	76	6	5075	SPAC144.11,SPBC19F8.08,SPCC285.15C,SPAC31G5.03,SPAC328.10C,SPAC13G6.02C
GO:CC	endosome	GO:0005768	0.012302613	1.910002648	121	76	8	5075	SPAC6G9.12,SPAPJ696.01C,SPAC16E8.01,SPCC594.06C,SPBC1734.08,SPBC9B6.03,SPBC18H10.19,SPBC215.14C
GO:CC	ribonucleoprotein complex	GO:1990904	0.021913264	1.65929293	542	76	18	5075	SPAC3G9.03,SPAC144.11,SPBC19F8.08,SPBC839.13C,SPAC19B12.11C,SPBP16F5.05C,SPCC330.14C,SPBC577.02,SPCC1223.05C,SPCC285.15C,SPAC31G5.03,SPBC776.11,SPBC1921.01C,SPAC328.10C,SPAC644.15,SPCC1682.14,SPCP31B10.07,SPAC13G6.02C
GO:CC	ESCRT complex	GO:0036452	0.022329154	1.65112774	14	76	3	5075	SPBC1734.08,SPBC9B6.03,SPBC215.14C
GO:CC	vesicle	GO:0031982	0.022329154	1.65112774	209	76	10	5075	SPAC6G9.12,SPAPJ696.01C,SPAC16E8.01,SPCC1919.10C,SPCC594.06C,SPBC1734.08,SPAC30D11.05,SPBC9B6.03,SPBC18H10.19,SPBC215.14C
GO:CC	protein-containing complex	GO:0032991	0.027265304	1.564389658	1984	76	43	5075	SPAC17G6.04C,SPAC3G9.03,SPAC6G9.12,SPCC18B5.10C,SPAC1486.04C,SPAC144.11,SPBC19F8.08,SPAPJ696.01C,SPBC839.13C,SPAC323.05C,SPAC19B12.11C,SPBP16F5.05C,SPAC16E8.01,SPCC330.14C,SPBC1711.03,SPAC1F3.03,SPCC1919.10C,SPBC577.02,SPBP35G2.08C,SPCC594.06C,SPCC330.11,SPCC1223.05C,SPCC285.15C,SPAC31G5.03,SPBC1734.13,SPBC1734.08,SPCC1739.14,SPBC776.11,SPAC30D11.05,SPBC9B6.03,SPBC8D2.04,SPAC19D5.11C,SPBC1921.01C,SPAC328.10C,SPAC644.15,SPCC1682.14,SPCP31B10.07,SPAC13G6.02C,SPAC29A4.18,SPBC18H10.19,SPBC215.14C,SPCC1235.06,SPAC26H5.05
GO:CC	endosome	GO:0010008	0.031352855	1.503722903	59	76	5	5075	SPAC16E8.01,SPBC1734.08,SPBC9B6.03,SPBC18H10.19,SPBC215.14C
GO:CC	endomembrane system	GO:0012505	0.034056623	1.467798414	1003	76	26	5075	SPBC1734.12C,SPAC6G9.12,SPAC1486.04C,SPBC887.17,SPAC1834.05,SPCC613.03,SPAPJ696.01C,SPCPB1C11.01,SPAC16E8.01,SPBC1711.03,SPBC1604.03C,SPAC13G6.10C,SPCC594.06C,SPBC3D6.04C,SPAPB17E12.08,SPBC2G2.01C,SPBC1734.08,SPCC1739.14,SPAC30D11.05,SPCC594.04C,SPBC9B6.03,SPCC320.05,SPBC18H10.19,SPBC215.14C,SPCC1235.06,SPAC26H5.05

GO:CC	small ribosomal subunit	GO:0015935	0.040515632	1.392377386	94	76	6	5075	SPAC144.11,SPBC19F8.08,SPCC285.15C,SPAC31G5.03,SPAC328.10C,SPAC13G6.02C
GO:CC	cytoplasmic vesicle	GO:0031410	0.04361852	1.360329072	205	76	9	5075	SPAC6G9.12,SPAPJ696.01C,SPAC16E8.01,SPCC594.06C,SPBC1734.08,SPAC30D11.05,SPBC9B6.03,SPBC18H10.19,SPBC215.14C
GO:CC	intracellular vesicle	GO:0097708	0.04361852	1.360329072	205	76	9	5075	SPAC6G9.12,SPAPJ696.01C,SPAC16E8.01,SPCC594.06C,SPBC1734.08,SPAC30D11.05,SPBC9B6.03,SPBC18H10.19,SPBC215.14C
KEGG	Ribosome	KEGG:03010	2.52231E-06	5.598201085	220	30	15	1959	SPAC3G9.03,SPAC144.11,SPBC19F8.08,SPBC839.13C,SPCC330.14C,SPBC577.02,SPCC1223.05C,SPCC285.15C,SPAC31G5.03,SPBC776.11,SPBC1921.01C,SPAC328.10C,SPAC644.15,SPCC1682.14,SPAC13G6.02C

Supplymentary table 3: Negativer genetic interactions for *btn1(D363G)* vs *ade6*Δ control
None enriched

Supplymentary table 4: Positive genetic interactions for *btn1(D363G)* vs *ade6*Δ control

source	term_name	term_id	adjusted_p_value	negative_log10_of_adjusted_p_value	term_size	query_size	intersection_size	effective_domain_size	intersections
GO:MF	structural constituent of ribosome	GO:0003735	8.53275E-06	5.068910914	218	75	16	5032	SPBC577.02,SPBC19F8.08,SPAC3G9.03,SPBC21B10.10,SPAC644.15,SPCC330.14C,SPCC1223.05C,SPAC144.11,SPBC839.13C,SPBC685.06,SPBC18H10.13,SPBC839.05C,SPBC776.11,SPBC1921.01C,SPCC970.05,SPCC285.15C
GO:MF	structural molecule activity	GO:0005198	8.53275E-06	5.068910914	274	75	18	5032	SPAC1486.04C,SPBC577.02,SPBC19F8.08,SPAC3G9.03,SPBC21B10.10,SPAC644.15,SPCC330.14C,SPCC1223.05C,SPAC144.11,SPBC839.13C,SPBC685.06,SPBC18H10.13,SPBC839.05C,SPBC776.11,SPBC1921.01C,SPCC970.05,SPCC285.15C,SPCC1739.14
GO:MF	rRNA binding	GO:0019843	0.00164141	2.784782923	59	75	7	5032	SPBC19F8.08,SPAC3G9.03,SPBC21B10.10,SPCC1223.05C,SPAC144.11,SPBC18H10.13,SPBC1921.01C
GO:MF	transferase activity, transferring sulfur-containing groups	GO:0016782	0.056069283	1.251274994	14	75	3	5032	SPAPB2B4.02,SPCC1235.02,SPBC19C2.13C
GO:BP	cytoplasmic translation	GO:0002181	3.82666E-05	4.417180476	269	75	18	5044	SPBC577.02,SPBC19F8.08,SPAC3G9.03,SPBC21B10.10,SPAC644.15,SPCC330.14C,SPCC1223.05C,SPAC323.05C,SPAC144.11,SPBC839.13C,SPBC685.06,SPBC18H10.13,SPBC839.05C,SPBC776.11,SPBC1921.01C,SPCC1223.01,SPCC970.05,SPCC285.15C
GO:BP	organonitrogen compound biosynthetic process	GO:1901566	0.001119529	2.950964556	873	75	30	5044	SPAC23A1.03,SPBC1734.12C,SPBC577.02,SPBC19F8.08,SPAC3G9.03,SPAC1834.05,SPBC1734.13,SPBC21B10.10,SPAC644.15,SPAC16.03C,SPCC330.14C,SPCC1223.05C,SPAC323.05C,SPCC1235.02,SPAC144.11,SPAC13C5.05C,SPAC637.06,SPBC839.13C,SPBC685.06,SPBC18H10.13,SPBC839.05C,SPAC144.04C,SPBC776.11,SPBC725.15,SPBC1921.01C,SPCC1223.01,SPCC970.05,SPAC14C4.14,SPCC285.15C,SPBC15D4.09C
GO:BP	amide biosynthetic process	GO:0043604	0.004878676	2.311698014	485	75	20	5044	SPBC577.02,SPBC19F8.08,SPAC3G9.03,SPBC21B10.10,SPAC644.15,SPCC330.14C,SPCC1223.05C,SPAC323.05C,SPCC1235.02,SPAC144.11,SPBC839.13C,SPBC685.06,SPBC18H10.13,SPBC839.05C,SPAC144.04C,SPBC776.11,SPBC1921.01C,SPCC1223.01,SPCC970.05,SPCC285.15C
GO:BP	translation	GO:0006412	0.008343154	2.078669745	428	75	18	5044	SPBC577.02,SPBC19F8.08,SPAC3G9.03,SPBC21B10.10,SPAC644.15,SPCC330.14C,SPCC1223.05C,SPAC323.05C,SPAC144.11,SPBC839.13C,SPBC685.06,SPBC18H10.13,SPBC839.05C,SPBC776.11,SPBC1921.01C,SPCC1223.01,SPCC970.05,SPCC285.15C
GO:BP	peptide biosynthetic process	GO:0043043	0.009091344	2.041371932	438	75	18	5044	SPBC577.02,SPBC19F8.08,SPAC3G9.03,SPBC21B10.10,SPAC644.15,SPCC330.14C,SPCC1223.05C,SPAC323.05C,SPAC144.11,SPBC839.13C,SPBC685.06,SPBC18H10.13,SPBC839.05C,SPBC776.11,SPBC1921.01C,SPCC1223.01,SPCC970.05,SPCC285.15C
GO:BP	cellular amide metabolic process	GO:0043603	0.011664407	1.933137326	540	75	20	5044	SPBC577.02,SPBC19F8.08,SPAC3G9.03,SPBC21B10.10,SPAC644.15,SPCC330.14C,SPCC1223.05C,SPAC323.05C,SPCC1235.02,SPAC144.11,SPBC839.13C,SPBC685.06,SPBC18H10.13,SPBC839.05C,SPAC144.04C,SPBC776.11,SPBC1921.01C,SPCC1223.01,SPCC970.05,SPCC285.15C

GO:BP	peptide metabolic process	GO:0006518	0.011699667	1.931826512	458	75	18	5044	SPBC577.02,SPBC19F8.08,SPAC3G9.03,SPBC21B10.10,SPAC644.15,SPCC330.14C,SPCC1223.05C,SPAC323.05C,SPAC144.11,SPBC839.13C,SPBC685.06,SPBC18H10.13,SPBC839.05C,SPBC776.11,SPBC1921.01C,SPCC1223.01,SPCC970.05,SPCC285.15C
GO:BP	ribonucleoprotein complex localization	GO:0071166	0.071495729	1.145719899	75	75	6	5044	SPAC1486.04C,SPCC18B5.10C,SPBC1921.03C,SPAC19B12.11C,SPCC285.15C,SPCC1739.14
GO:BP	ribonucleoprotein complex export from nucleus	GO:0071426	0.071495729	1.145719899	75	75	6	5044	SPAC1486.04C,SPCC18B5.10C,SPBC1921.03C,SPAC19B12.11C,SPCC285.15C,SPCC1739.14
GO:BP	formin-nucleated actin cable organization	GO:0110009	0.071495729	1.145719899	3	75	2	5044	SPBC1778.06C,SPCC1919.10C
GO:BP	RNA export from nucleus	GO:0006405	0.085611915	1.067465787	79	75	6	5044	SPAC1486.04C,SPCC18B5.10C,SPBC1921.03C,SPAC19B12.11C,SPCC285.15C,SPCC1739.14
GO:CC	cytosolic ribosome	GO:0022626	1.15E-07	6.937980811	156	75	16	5075	SPBC577.02,SPBC19F8.08,SPAC3G9.03,SPBC21B10.10,SPAC644.15,SPCC330.14C,SPCC1223.05C,SPAC144.11,SPBC839.13C,SPBC685.06,SPBC18H10.13,SPBC839.05C,SPBC776.11,SPBC1921.01C,SPCC970.05,SPCC285.15C
GO:CC	ribosomal subunit	GO:0044391	1.34607E-05	4.870932041	227	75	16	5075	SPBC577.02,SPBC19F8.08,SPAC3G9.03,SPBC21B10.10,SPAC644.15,SPCC330.14C,SPCC1223.05C,SPAC144.11,SPBC839.13C,SPBC685.06,SPBC18H10.13,SPBC839.05C,SPBC776.11,SPBC1921.01C,SPCC970.05,SPCC285.15C
GO:CC	ribosome	GO:0005840	2.05852E-05	4.686445088	241	75	16	5075	SPBC577.02,SPBC19F8.08,SPAC3G9.03,SPBC21B10.10,SPAC644.15,SPCC330.14C,SPCC1223.05C,SPAC144.11,SPBC839.13C,SPBC685.06,SPBC18H10.13,SPBC839.05C,SPBC776.11,SPBC1921.01C,SPCC970.05,SPCC285.15C
GO:CC	cytosolic large ribosomal subunit	GO:0022625	0.000277876	3.556148595	89	75	9	5075	SPBC577.02,SPAC3G9.03,SPAC644.15,SPCC330.14C,SPCC1223.05C,SPBC839.13C,SPBC776.11,SPBC1921.01C,SPCC970.05
GO:CC	cytosolic small ribosomal subunit	GO:0022627	0.001608633	2.793542986	64	75	7	5075	SPBC19F8.08,SPBC21B10.10,SPAC144.11,SPBC685.06,SPBC18H10.13,SPBC839.05C,SPCC285.15C
GO:CC	large ribosomal subunit	GO:0015934	0.005281803	2.277217823	135	75	9	5075	SPBC577.02,SPAC3G9.03,SPAC644.15,SPCC330.14C,SPCC1223.05C,SPBC839.13C,SPBC776.11,SPBC1921.01C,SPCC970.05
GO:CC	small ribosomal subunit	GO:0015935	0.013295545	1.876293847	94	75	7	5075	SPBC19F8.08,SPBC21B10.10,SPAC144.11,SPBC685.06,SPBC18H10.13,SPBC839.05C,SPCC285.15C
GO:CC	ribonucleoprotein complex	GO:1990904	0.018949314	1.722406506	542	75	18	5075	SPBC577.02,SPBC19F8.08,SPAC3G9.03,SPBP16F5.05C,SPBC21B10.10,SPAC644.15,SPCC330.14C,SPCC1223.05C,SPAC144.11,SPBC839.13C,SPBC685.06,SPBC18H10.13,SPBC839.05C,SPAC19B12.11C,SPBC776.11,SPBC1921.01C,SPCC970.05,SPCC285.15C
GO:CC	proton-transporting ATP synthase complex, catalytic core F(1)	GO:0045261	0.045856908	1.338595229	5	75	2	5075	SPBC1734.13,SPAC14C4.14
GO:CC	mitochondrial proton-transporting ATP synthase complex, catalytic sector F(1)	GO:0000275	0.045856908	1.338595229	5	75	2	5075	SPBC1734.13,SPAC14C4.14

KEGG	Ribosome	KEGG:03010	4.55085E-05	4.34190731	220	39	16	1959	SPBC577.02,SPBC19F8.08,SPAC3G9.03,SPBC21B10.10,SPAC644.15,SPCC330.14C,SPCC1223.05C,SPAC144.11,SPBC839.13C,SPBC685.06,SPBC18H10.13,SPBC839.05C,SPBC776.11,SPBC1921.01C,SPCC970.05,SPCC285.15C
KEGG	Various types of N-glycan biosynthesis	KEGG:00513	0.096426761	1.015802422	19	39	3	1959	SPBC1734.12C,SPAC1834.05,SPAC637.06

Supplymentary table 5: Negative genetic interactions for *btn1(102-208del)* vs *ade6*Δ control

source	term_name	term_id	adjusted_p_value	negative_log10_of_adjusted_p_value	term_size	query_size	intersection_size	effective_domain_size	intersections
GO:BP	positive regulation of cellular process	GO:0048522	0.007940175	2.100169949	575	178	41	5044	SPBC1D7.03,SPBC543.07,SPAC4H3.13,SPAC458.05,SPAC3A11.08,SPBC646.13,SPAC4F10.15C,SPBC28F2.10C,SPBC409.07C,SPAC4C5.02C,SPCC594.05C,SPAC26H5.10C,SPBC3H7.15,SPBC428.08C,SPAC2F3.15,SPBC30D10.10C,SPAC5D6.05,SPBC2D10.06,SPCC1494.10,SPAC16C9.05,SPBC646.09C,SPBC23E6.08,SPAC6G9.15C,SPBC887.10,SPBC776.02C,SPBC12D12.06,SPBC3E7.01,SPAC1556.08C,SPBC21C3.20C,SPBC106.16,SPAC57A7.04C,SPBC13E7.08C,SPAC11E3.05,SPAC6F12.02,SPBC1685.08,SPBC83.03C,SPAC644.06C,SPCC126.04C,SPAC31A2.11C,SPAC513.03,SPCC74.03C
GO:BP	positive regulation of biological process	GO:0048518	0.007940175	2.100169949	587	178	41	5044	SPBC1D7.03,SPBC543.07,SPAC4H3.13,SPAC458.05,SPAC3A11.08,SPBC646.13,SPAC4F10.15C,SPBC28F2.10C,SPBC409.07C,SPAC4C5.02C,SPCC594.05C,SPAC26H5.10C,SPBC3H7.15,SPBC428.08C,SPAC2F3.15,SPBC30D10.10C,SPAC5D6.05,SPBC2D10.06,SPCC1494.10,SPAC16C9.05,SPBC646.09C,SPBC23E6.08,SPAC6G9.15C,SPBC887.10,SPBC776.02C,SPBC12D12.06,SPBC3E7.01,SPAC1556.08C,SPBC21C3.20C,SPBC106.16,SPAC57A7.04C,SPBC13E7.08C,SPAC11E3.05,SPAC6F12.02,SPBC1685.08,SPBC83.03C,SPAC644.06C,SPCC126.04C,SPAC31A2.11C,SPAC513.03,SPCC74.03C
GO:BP	cellular response to osmotic stress	GO:0071470	0.035589605	1.448676828	24	178	6	5044	SPBC409.07C,SPBC30D10.10C,SPBC887.10,SPAC1A6.04C,SPAC644.06C,SPCC74.03C
GO:BP	biological regulation	GO:0065007	0.035589605	1.448676828	1563	178	78	5044	SPBC1D7.03,SPBC543.07,SPAC4H3.13,SPAC13G6.15C,SPAC4G8.10,SPBC530.08,SPAC458.05,SPAC3A11.08,SPAC4C5.04,SPBC646.13,SPBC2D10.13,SPAC4F10.15C,SPCC790.02,SPAC19G12.13C,SPBC28F2.10C,SPBC409.07C,SPAC4C5.02C,SPCC594.05C,SPAC26H5.10C,SPBC1105.10,SPBC3H7.15,SPBC428.08C,SPAC2F3.15,SPBC30D10.10C,SPBC3F6.01C,SPAC5D6.05,SPBC2D10.06,SPCC1494.10,SPAC16C9.05,SPBC646.09C,SPBC11B10.07C,SPBC23E6.08,SPAC6G9.15C,SPBC887.10,SPBC4F6.06,SPBC776.02C,SPBC32F12.01C,SPBC947.08C,SPBC12D12.06,SPBC21B10.03C,SPAC26F1.10C,SPBC3E7.01,SPAC1556.08C,SPAC8F11.02C,SPBC336.03,SPCC1919.03C,SPAC15A10.06,SPAC1D4.01,SPAC22E12.19,SPBC21C3.20C,SPAC23G3.03,SPBC106.16,SPAC57A7.04C,SPBC119.06,SPBC13E7.08C,SPAC11E3.05,SPCC1919.05,SPAC6F12.02,SPBC1685.08,SPAC1F7.07C,SPAC26H5.07C,SPBC83.03C,SPCPJ732.01,SPAC644.06C,SPAC6F12.06,SPAP8A3.13C,SPCC126.04C,SPAC1F3.10C,SPAC31A2.11C,SPBC21C3.02C,SPAC2F7.02C,SPAC513.03,SPAC3A11.09,SPBC16A3.19,SPCC16C4.09,SPBC13G1.08C,SPBC3B8.10C,SPCC74.03C

GO:BP	regulation of cellular process	GO:0050794	0.035589605	1.448676828	1257	178	67	5044	SPBC1D7.03,SPBC543.07,SPAC4H3.13,SPAC13G6.15C,SPAC4G8.10,SPBC530.08,SPAC458.05,SPAC3A11.08,SPAC4C5.04,SPBC646.13,SPBC2D10.13,SPAC4F10.15C,SPCC790.02,SPAC19G12.13C,SPBC28F2.10C,SPBC409.07C,SPAC4C5.02C,SPCC594.05C,SPAC26H5.10C,SPBC3H7.15,SPBC428.08C,SPAC2F3.15,SPBC30D10.10C,SPBC3F6.01C,SPAC5D6.05,SPBC2D10.06,SPCC1494.10,SPAC16C9.05,SPBC646.09C,SPBC23E6.08,SPAC6G9.15C,SPBC887.10,SPBC4F6.06,SPBC776.02C,SPBC32F12.01C,SPBC947.08C,SPBC12D12.06,SPBC21B10.03C,SPAC26F1.10C,SPBC3E7.01,SPAC1556.08C,SPAC8F11.02C,SPBC336.03,SPCC1919.03C,SPAC1D4.01,SPAC22E12.19,SPBC21C3.20C,SPBC106.16,SPAC57A7.04C,SPBC13E7.08C,SPAC11E3.05,SPAC6F12.02,SPBC1685.08,SPAC26H5.07C,SPBC83.03C,SPAC644.06C,SPAC6F12.06,SPAP8A3.13C,SPCC126.04C,SPAC31A2.11C,SPBC21C3.02C,SPAC2F7.02C,SPAC513.03,SPBC16A3.19,SPBC13G1.08C,SPBC3B8.10C,SPCC74.03C
GO:BP	regulation of biological process	GO:0050789	0.035589605	1.448676828	1358	178	70	5044	SPBC1D7.03,SPBC543.07,SPAC4H3.13,SPAC13G6.15C,SPAC4G8.10,SPBC530.08,SPAC458.05,SPAC3A11.08,SPAC4C5.04,SPBC646.13,SPBC2D10.13,SPAC4F10.15C,SPCC790.02,SPAC19G12.13C,SPBC28F2.10C,SPBC409.07C,SPAC4C5.02C,SPCC594.05C,SPAC26H5.10C,SPBC3H7.15,SPBC428.08C,SPAC2F3.15,SPBC30D10.10C,SPBC3F6.01C,SPAC5D6.05,SPBC2D10.06,SPCC1494.10,SPAC16C9.05,SPBC646.09C,SPBC11B10.07C,SPBC23E6.08,SPAC6G9.15C,SPBC887.10,SPBC4F6.06,SPBC776.02C,SPBC32F12.01C,SPBC947.08C,SPBC12D12.06,SPBC21B10.03C,SPAC26F1.10C,SPBC3E7.01,SPAC1556.08C,SPAC8F11.02C,SPBC336.03,SPCC1919.03C,SPAC1D4.01,SPAC22E12.19,SPBC21C3.20C,SPBC106.16,SPAC57A7.04C,SPBC13E7.08C,SPAC11E3.05,SPCC1919.05,SPAC6F12.02,SPBC1685.08,SPAC26H5.07C,SPBC83.03C,SPAC644.06C,SPAC6F12.06,SPAP8A3.13C,SPCC126.04C,SPAC31A2.11C,SPBC21C3.02C,SPAC2F7.02C,SPAC513.03,SPBC16A3.19,SPCC16C4.09,SPBC13G1.08C,SPBC3B8.10C,SPCC74.03C
GO:BP	response to osmotic stress	GO:0006970	0.035589605	1.448676828	24	178	6	5044	SPBC409.07C,SPBC30D10.10C,SPBC887.10,SPAC1A6.04C,SPAC644.06C,SPCC74.03C
GO:BP	cellular response to glucose starvation	GO:0042149	0.035589605	1.448676828	15	178	5	5044	SPBC646.13,SPBC30D10.10C,SPCC1919.03C,SPAC6F12.02,SPCC74.03C
GO:BP	signaling	GO:0023052	0.036780608	1.434381092	410	178	29	5044	SPBC1D7.03,SPBC543.07,SPAC13G6.15C,SPAC458.05,SPBC646.13,SPBC409.07C,SPAC4C5.02C,SPBC3H7.15,SPAC2F3.15,SPBC30D10.10C,SPBC3F6.01C,SPBC23E6.08,SPBC887.10,SPBC4F6.06,SPBC776.02C,SPAC26F1.10C,SPBC3E7.01,SPAC1556.08C,SPBC336.03,SPCC1919.03C,SPBC21C3.20C,SPAC11E3.05,SPAC26H5.07C,SPAC644.06C,SPAC6F12.06,SPAC2F7.02C,SPAC513.03,SPBC3B8.10C,SPCC74.03C
GO:BP	protein modification process	GO:0036211	0.036780608	1.434381092	765	178	45	5044	SPBC2F12.15C,SPBC1D7.03,SPBC543.07,SPAC13G6.15C,SPAC3A11.08,SPAC4C5.04,SPBC646.13,SPCC790.02,SPBC17A3.09C,SPBC2A9.06C,SPBC28F2.10C,SPBC409.07C,SPCC594.05C,SPBC3H7.15,SPBC428.08C,SPAC2F3.15,SPAC19B12.10,SPBC30D10.10C,SPBC354.03,SPBC3F6.01C,SPAC16C9.05,SPBC887.10,SPBC4F6.06,SPBC776.02C,SPBC12D12.06,SPBC1703.12,SPAC26F1.10C,SPBC3E7.01,SPAC1556.08C,SPAC8F11.02C,SPCC1919.03C,SPAC22E12.19,SPBC13E7.08C,SPAC11E3.05,SPBC1685.08,SPAC644.06C,SPBC21C3.02C,SPAC2F7.02C,SPAC959.04C,SPBC16A3.19,SPBC3E7.10,SPBC13G1.08C,SPBC3B8.10C,SPBC1709.04C,SPCC74.03C

GO:BP	cellular protein modification process	GO:0006464	0.036780608	1.434381092	765	178	45	5044	SPBC2F12.15C,SPBC1D7.03,SPBC543.07,SPAC13G6.15C,SPAC3A11.08,SPAC4C5.04,SPBC646.13,SPCC790.02,SPBC17A3.09C,SPBC2A9.06C,SPBC28F2.10C,SPBC409.07C,SPCC594.05C,SPBC3H7.15,SPBC428.08C,SPAC2F3.15,SPAC19B12.10,SPBC30D10.10C,SPBC354.03,SPBC3F6.01C,SPAC16C9.05,SPBC887.10,SPBC4F6.06,SPBC776.02C,SPBC12D12.06,SPBC1703.12,SPAC26F1.10C,SPBC3E7.01,SPAC1556.08C,SPAC8F11.02C,SPCC1919.03C,SPAC22E12.19,SPBC13E7.08C,SPAC11E3.05,SPBC1685.08,SPAC644.06C,SPBC21C3.02C,SPAC2F7.02C,SPAC959.04C,SPBC16A3.19,SPBC3E7.10,SPBC13G1.08C,SPBC3B8.10C,SPBC1709.04C,SPCC74.03C
GO:BP	regulation of cellular metabolic process	GO:0031323	0.039941282	1.398577995	817	178	47	5044	SPBC1D7.03,SPBC543.07,SPAC4H3.13,SPAC13G6.15C,SPBC530.08,SPAC3A11.08,SPBC646.13,SPBC2D10.13,SPAC19G12.13C,SPBC28F2.10C,SPBC409.07C,SPCC594.05C,SPAC26H5.10C,SPBC3H7.15,SPBC428.08C,SPAC2F3.15,SPBC30D10.10C,SPAC5D6.05,SPBC2D10.06,SPCC1494.10,SPAC16C9.05,SPBC646.09C,SPAC6G9.15C,SPBC887.10,SPBC776.02C,SPBC947.08C,SPBC12D12.06,SPAC26F1.10C,SPAC1556.08C,SPAC8F11.02C,SPCC1919.03C,SPAC1D4.01,SPAC22E12.19,SPAC57A7.04C,SPBC13E7.08C,SPAC6F12.02,SPBC1685.08,SPBC83.03C,SPAP8A3.13C,SPCC126.04C,SPAC31A2.11C,SPBC21C3.02C,SPAC2F7.02C,SPBC16A3.19,SPBC13G1.08C,SPBC3B8.10C,SPCC74.03C
GO:BP	regulation of primary metabolic process	GO:0080090	0.041789915	1.378928514	798	178	46	5044	SPBC1D7.03,SPBC543.07,SPAC4H3.13,SPAC13G6.15C,SPBC530.08,SPAC3A11.08,SPBC646.13,SPBC2D10.13,SPAC19G12.13C,SPBC28F2.10C,SPBC409.07C,SPCC594.05C,SPAC26H5.10C,SPBC3H7.15,SPBC428.08C,SPAC2F3.15,SPAC5D6.05,SPBC2D10.06,SPCC1494.10,SPAC16C9.05,SPBC646.09C,SPAC6G9.15C,SPBC887.10,SPBC776.02C,SPBC947.08C,SPBC12D12.06,SPAC26F1.10C,SPAC1556.08C,SPAC8F11.02C,SPCC1919.03C,SPAC1D4.01,SPAC22E12.19,SPAC57A7.04C,SPBC13E7.08C,SPAC6F12.02,SPBC1685.08,SPBC83.03C,SPAP8A3.13C,SPCC126.04C,SPAC31A2.11C,SPBC21C3.02C,SPAC2F7.02C,SPBC16A3.19,SPBC13G1.08C,SPBC3B8.10C,SPCC74.03C
GO:BP	cytosolic transport	GO:0016482	0.060142974	1.220815103	41	178	7	5044	SPBC25H2.16C,SPCC790.02,SPAC4C5.02C,SPBC23E6.08,SPCC794.11C,SPCPJ732.01,SPAC15E1.06
GO:BP	regulation of nitrogen compound metabolic process	GO:0051171	0.061421305	1.211680957	772	178	44	5044	SPBC1D7.03,SPBC543.07,SPAC4H3.13,SPAC13G6.15C,SPBC530.08,SPAC3A11.08,SPBC646.13,SPBC2D10.13,SPAC19G12.13C,SPBC28F2.10C,SPBC409.07C,SPCC594.05C,SPAC26H5.10C,SPBC3H7.15,SPBC428.08C,SPAC2F3.15,SPAC5D6.05,SPBC2D10.06,SPCC1494.10,SPAC16C9.05,SPBC646.09C,SPAC6G9.15C,SPBC887.10,SPBC776.02C,SPBC947.08C,SPBC12D12.06,SPAC26F1.10C,SPAC1556.08C,SPAC8F11.02C,SPCC1919.03C,SPAC1D4.01,SPAC22E12.19,SPAC57A7.04C,SPBC13E7.08C,SPAC6F12.02,SPBC1685.08,SPBC83.03C,SPCC126.04C,SPAC31A2.11C,SPBC21C3.02C,SPAC2F7.02C,SPBC16A3.19,SPBC13G1.08C,SPCC74.03C
GO:BP	macromolecule modification	GO:0043412	0.061421305	1.211680957	866	178	48	5044	SPBC2F12.15C,SPBC1D7.03,SPBC543.07,SPAC4H3.13,SPAC13G6.15C,SPAC3A11.08,SPAC4C5.04,SPBC646.13,SPCC790.02,SPBC17A3.09C,SPBC2A9.06C,SPBC28F2.10C,SPBC409.07C,SPCC594.05C,SPBC3H7.15,SPBC428.08C,SPAC2F3.15,SPAC19B12.10,SPBC30D10.10C,SPBC354.03,SPBC3F6.01C,SPAC222.05C,SPAC16C9.05,SPBC887.10,SPBC4F6.06,SPBC776.02C,SPBC12D12.06,SPBC1703.12,SPAC26F1.10C,SPBC3E7.01,SPAC1556.08C,SPAC8F11.02C,SPBC21C3.07C,SPCC1919.03C,SPAC22E12.19,SPBC13E7.08C,SPAC11E3.05,SPBC1685.08,SPAC644.06C,SPBC21C3.02C,SPAC2F7.02C,SPAC959.04C,SPBC16A3.19,SPBC3E7.10,SPBC13G1.08C,SPBC3B8.10C,SPBC1709.04C,SPCC74.03C

GO:BP	positive regulation of cellular metabolic process	GO:0031325	0.063118118	1.19984596	356	178	25	5044	SPBC1D7.03,SPBC543.07,SPAC4H3.13,SPBC28F2.10C,SPBC409.07C,SPCC594.05C,SPAC26H5.10C,SPBC3H7.15,SPAC2F3.15,SPAC5D6.05,SPBC2D10.06,SPCC1494.10,SPAC16C9.05,SPBC646.09C,SPAC6G9.15C,SPBC887.10,SPBC776.02C,SPBC12D12.06,SPAC1556.08C,SPAC57A7.04C,SPBC13E7.08C,SPAC6F12.02,SPBC1685.08,SPCC126.04C,SPAC31A2.11C
GO:BP	regulation of transcription by RNA polymerase II	GO:0006357	0.07170088	1.144475512	340	178	24	5044	SPAC4H3.13,SPBC530.08,SPAC3A11.08,SPBC28F2.10C,SPBC428.08C,SPAC2F3.15,SPAC5D6.05,SPBC2D10.06,SPCC1494.10,SPAC16C9.05,SPAC6G9.15C,SPBC776.02C,SPBC12D12.06,SPAC1556.08C,SPAC22E12.19,SPBC13E7.08C,SPAC6F12.02,SPBC1685.08,SPCC126.04C,SPAC31A2.11C,SPBC21C3.02C,SPAC2F7.02C,SPBC16A3.19,SPBC13G1.08C
GO:BP	positive regulation of reproductive process	GO:2000243	0.07231457	1.140774193	86	178	10	5044	SPAC458.05,SPAC3A11.08,SPBC646.13,SPBC3H7.15,SPBC30D10.10C,SPBC3E7.01,SPBC21C3.20C,SPAC6F12.02,SPAC513.03,SPCC74.03C
GO:BP	positive regulation of nitrogen compound metabolic process	GO:0051173	0.075219887	1.123667322	346	178	24	5044	SPBC543.07,SPAC4H3.13,SPBC28F2.10C,SPBC409.07C,SPCC594.05C,SPAC26H5.10C,SPBC3H7.15,SPAC2F3.15,SPAC5D6.05,SPBC2D10.06,SPCC1494.10,SPAC16C9.05,SPBC646.09C,SPAC6G9.15C,SPBC887.10,SPBC776.02C,SPBC12D12.06,SPAC1556.08C,SPAC57A7.04C,SPBC13E7.08C,SPAC6F12.02,SPBC1685.08,SPCC126.04C,SPAC31A2.11C
GO:BP	regulation of metabolic process	GO:0019222	0.075219887	1.123667322	910	178	49	5044	SPBC1D7.03,SPBC543.07,SPAC4H3.13,SPAC13G6.15C,SPBC530.08,SPAC3A11.08,SPBC646.13,SPBC2D10.13,SPAC19G12.13C,SPBC28F2.10C,SPBC409.07C,SPCC594.05C,SPAC26H5.10C,SPBC3H7.15,SPBC428.08C,SPAC2F3.15,SPBC30D10.10C,SPAC5D6.05,SPBC2D10.06,SPCC1494.10,SPAC16C9.05,SPBC646.09C,SPAC6G9.15C,SPBC887.10,SPBC776.02C,SPBC947.08C,SPBC12D12.06,SPAC26F1.10C,SPAC1556.08C,SPAC8F11.02C,SPCC1919.03C,SPAC1D4.01,SPAC22E12.19,SPAC57A7.04C,SPBC13E7.08C,SPCC1919.05,SPAC6F12.02,SPBC1685.08,SPBC83.03C,SPAP8A3.13C,SPCC126.04C,SPAC31A2.11C,SPBC21C3.02C,SPAC2F7.02C,SPBC16A3.19,SPCC16C4.09,SPBC13G1.08C,SPBC3B8.10C,SPCC74.03C
GO:BP	positive regulation of metabolic process	GO:0009893	0.075219887	1.123667322	366	178	25	5044	SPBC1D7.03,SPBC543.07,SPAC4H3.13,SPBC28F2.10C,SPBC409.07C,SPCC594.05C,SPAC26H5.10C,SPBC3H7.15,SPAC2F3.15,SPAC5D6.05,SPBC2D10.06,SPCC1494.10,SPAC16C9.05,SPBC646.09C,SPAC6G9.15C,SPBC887.10,SPBC776.02C,SPBC12D12.06,SPAC1556.08C,SPAC57A7.04C,SPBC13E7.08C,SPAC6F12.02,SPBC1685.08,SPCC126.04C,SPAC31A2.11C
GO:BP	multicellular organism development	GO:0007275	0.075350113	1.12291609	2	178	2	5044	SPBC2A9.06C,SPCC74.03C
GO:BP	regulation of nucleobase-containing compound metabolic process	GO:0019219	0.075350113	1.12291609	572	178	34	5044	SPAC4H3.13,SPBC530.08,SPAC3A11.08,SPBC2D10.13,SPAC19G12.13C,SPBC28F2.10C,SPCC594.05C,SPBC3H7.15,SPBC428.08C,SPAC2F3.15,SPAC5D6.05,SPBC2D10.06,SPCC1494.10,SPAC16C9.05,SPAC6G9.15C,SPBC776.02C,SPBC947.08C,SPBC12D12.06,SPAC26F1.10C,SPAC1556.08C,SPAC1D4.01,SPAC22E12.19,SPAC57A7.04C,SPBC13E7.08C,SPAC6F12.02,SPBC1685.08,SPBC83.03C,SPCC126.04C,SPAC31A2.11C,SPBC21C3.02C,SPAC2F7.02C,SPBC16A3.19,SPBC13G1.08C,SPCC74.03C

GO:BP	regulation of nucleic acid-templated transcription	GO:1903506	0.075350113	1.12291609	463	178	29	5044	SPAC4H3.13,SPBC530.08,SPAC3A11.08,SPBC28F2.10C,SPCC594.05C,SPBC428.08C,SPAC2F3.15,SPAC5D6.05,SPBC2D10.06,SPCC1494.10,SPAC16C9.05,SPAC6G9.15C,SPBC776.02C,SPBC947.08C,SPBC12D12.06,SPAC26F1.10C,SPAC1556.08C,SPAC22E12.19,SPBC13E7.08C,SPAC6F12.02,SPBC1685.08,SPBC83.03C,SPCC126.04C,SPAC31A2.11C,SPBC21C3.02C,SPAC2F7.02C,SPBC16A3.19,SPBC13G1.08C,SPCC74.03C
GO:BP	regulation of transcription, DNA-templated	GO:0006355	0.075350113	1.12291609	457	178	29	5044	SPAC4H3.13,SPBC530.08,SPAC3A11.08,SPBC28F2.10C,SPCC594.05C,SPBC428.08C,SPAC2F3.15,SPAC5D6.05,SPBC2D10.06,SPCC1494.10,SPAC16C9.05,SPAC6G9.15C,SPBC776.02C,SPBC947.08C,SPBC12D12.06,SPAC26F1.10C,SPAC1556.08C,SPAC22E12.19,SPBC13E7.08C,SPAC6F12.02,SPBC1685.08,SPBC83.03C,SPCC126.04C,SPAC31A2.11C,SPBC21C3.02C,SPAC2F7.02C,SPBC16A3.19,SPBC13G1.08C,SPCC74.03C
GO:BP	regulation of cellular biosynthetic process	GO:0031326	0.075350113	1.12291609	620	178	36	5044	SPAC4H3.13,SPBC530.08,SPAC3A11.08,SPBC2D10.13,SPAC19G12.13C,SPBC28F2.10C,SPCC594.05C,SPAC26H5.10C,SPBC428.08C,SPAC2F3.15,SPAC5D6.05,SPBC2D10.06,SPCC1494.10,SPAC16C9.05,SPAC6G9.15C,SPBC776.02C,SPBC947.08C,SPBC12D12.06,SPAC26F1.10C,SPAC1556.08C,SPAC8F11.02C,SPAC22E12.19,SPAC57A7.04C,SPBC13E7.08C,SPAC6F12.02,SPBC1685.08,SPBC83.03C,SPAP8A3.13C,SPCC126.04C,SPAC31A2.11C,SPBC21C3.02C,SPAC2F7.02C,SPBC16A3.19,SPBC13G1.08C,SPBC3B8.10C,SPCC74.03C
GO:BP	regulation of biosynthetic process	GO:0009889	0.075350113	1.12291609	620	178	36	5044	SPAC4H3.13,SPBC530.08,SPAC3A11.08,SPBC2D10.13,SPAC19G12.13C,SPBC28F2.10C,SPCC594.05C,SPAC26H5.10C,SPBC428.08C,SPAC2F3.15,SPAC5D6.05,SPBC2D10.06,SPCC1494.10,SPAC16C9.05,SPAC6G9.15C,SPBC776.02C,SPBC947.08C,SPBC12D12.06,SPAC26F1.10C,SPAC1556.08C,SPAC8F11.02C,SPAC22E12.19,SPAC57A7.04C,SPBC13E7.08C,SPAC6F12.02,SPBC1685.08,SPBC83.03C,SPAP8A3.13C,SPCC126.04C,SPAC31A2.11C,SPBC21C3.02C,SPAC2F7.02C,SPBC16A3.19,SPBC13G1.08C,SPBC3B8.10C,SPCC74.03C
GO:BP	regulation of RNA metabolic process	GO:0051252	0.075350113	1.12291609	507	178	31	5044	SPAC4H3.13,SPBC530.08,SPAC3A11.08,SPBC28F2.10C,SPCC594.05C,SPBC428.08C,SPAC2F3.15,SPAC5D6.05,SPBC2D10.06,SPCC1494.10,SPAC16C9.05,SPAC6G9.15C,SPBC776.02C,SPBC947.08C,SPBC12D12.06,SPAC26F1.10C,SPAC1556.08C,SPAC1D4.01,SPAC22E12.19,SPAC57A7.04C,SPBC13E7.08C,SPAC6F12.02,SPBC1685.08,SPBC83.03C,SPCC126.04C,SPAC31A2.11C,SPBC21C3.02C,SPAC2F7.02C,SPBC16A3.19,SPBC13G1.08C,SPCC74.03C
GO:BP	system development	GO:0048731	0.075350113	1.12291609	2	178	2	5044	SPBC2A9.06C,SPCC74.03C
GO:BP	regulation of RNA biosynthetic process	GO:2001141	0.075350113	1.12291609	464	178	29	5044	SPAC4H3.13,SPBC530.08,SPAC3A11.08,SPBC28F2.10C,SPCC594.05C,SPBC428.08C,SPAC2F3.15,SPAC5D6.05,SPBC2D10.06,SPCC1494.10,SPAC16C9.05,SPAC6G9.15C,SPBC776.02C,SPBC947.08C,SPBC12D12.06,SPAC26F1.10C,SPAC1556.08C,SPAC22E12.19,SPBC13E7.08C,SPAC6F12.02,SPBC1685.08,SPBC83.03C,SPCC126.04C,SPAC31A2.11C,SPBC21C3.02C,SPAC2F7.02C,SPBC16A3.19,SPBC13G1.08C,SPCC74.03C
GO:BP	transcription by RNA polymerase II	GO:0006366	0.075350113	1.12291609	392	178	26	5044	SPAC4H3.13,SPBC530.08,SPBC4B4.03,SPAC3A11.08,SPBC28F2.10C,SPAC3H8.09C,SPBC428.08C,SPAC2F3.15,SPAC5D6.05,SPBC2D10.06,SPCC1494.10,SPAC16C9.05,SPAC6G9.15C,SPBC776.02C,SPBC12D12.06,SPAC1556.08C,SPAC22E12.19,SPBC13E7.08C,SPAC6F12.02,SPBC1685.08,SPCC126.04C,SPAC31A2.11C,SPBC21C3.02C,SPAC2F7.02C,SPBC16A3.19,SPBC13G1.08C

GO:BP	positive regulation of macromolecule metabolic process	GO:0010604	0.075350113	1.12291609	352	178	24	5044	SPBC543.07,SPAC4H3.13,SPBC28F2.10C,SPBC409.07C,SPCC594.05C,SPAC26H5.10C,SPBC3H7.15,SPAC2F3.15,SPAC5D6.05,SPBC2D10.06,SPCC1494.10,SPAC16C9.05,SPBC646.09C,SPAC6G9.15C,SPBC887.10,SPBC776.02C,SPBC12D12.06,SPAC1556.08C,SPAC57A7.04C,SPBC13E7.08C,SPAC6F12.02,SPBC1685.08,SPCC126.04C,SPAC31A2.11C
GO:BP	multicellular organismal process	GO:0032501	0.075350113	1.12291609	2	178	2	5044	SPBC2A9.06C,SPCC74.03C
GO:BP	histone modification	GO:0016570	0.085028337	1.070436315	129	178	12	5044	SPAC3A11.08,SPBC28F2.10C,SPCC594.05C,SPBC428.08C,SPBC354.03,SPAC16C9.05,SPAC22E12.19,SPBC13E7.08C,SPBC1685.08,SPBC21C3.02C,SPBC16A3.19,SPBC13G1.08C
GO:BP	covalent chromatin modification	GO:0016569	0.085028337	1.070436315	129	178	12	5044	SPAC3A11.08,SPBC28F2.10C,SPCC594.05C,SPBC428.08C,SPBC354.03,SPAC16C9.05,SPAC22E12.19,SPBC13E7.08C,SPBC1685.08,SPBC21C3.02C,SPBC16A3.19,SPBC13G1.08C
GO:BP	cell communication	GO:0007154	0.085028337	1.070436315	405	178	26	5044	SPBC543.07,SPAC13G6.15C,SPAC458.05,SPBC646.13,SPBC409.07C,SPAC4C5.02C,SPBC30D10.10C,SPBC3F6.01C,SPBC23E6.08,SPBC887.10,SPBC4F6.06,SPAC26F1.10C,SPBC3E7.01,SPAC1556.08C,SPBC336.03,SPCC1919.03C,SPBC21C3.20C,SPAC23G3.03,SPAC11E3.05,SPAC6F12.02,SPAC26H5.07C,SPAC644.06C,SPAC6F12.06,SPAC31A2.11C,SPAC513.03,SPCC74.03C
GO:BP	regulation of macromolecule metabolic process	GO:0060255	0.096194562	1.016849478	870	178	46	5044	SPBC1D7.03,SPBC543.07,SPAC4H3.13,SPAC13G6.15C,SPBC530.08,SPAC3A11.08,SPBC646.13,SPBC2D10.13,SPAC19G12.13C,SPBC28F2.10C,SPBC409.07C,SPCC594.05C,SPAC26H5.10C,SPBC3H7.15,SPBC428.08C,SPAC2F3.15,SPAC5D6.05,SPBC2D10.06,SPCC1494.10,SPAC16C9.05,SPBC646.09C,SPAC6G9.15C,SPBC887.10,SPBC776.02C,SPBC947.08C,SPBC12D12.06,SPAC26F1.10C,SPAC1556.08C,SPAC8F11.02C,SPCC1919.03C,SPAC1D4.01,SPAC22E12.19,SPAC57A7.04C,SPBC13E7.08C,SPCC1919.05,SPAC6F12.02,SPBC1685.08,SPBC83.03C,SPCC126.04C,SPAC31A2.11C,SPBC21C3.02C,SPAC2F7.02C,SPBC16A3.19,SPCC16C4.09,SPBC13G1.08C,SPCC74.03C
GO:BP	positive regulation of RNA metabolic process	GO:0051254	0.098591733	1.0061595	226	178	17	5044	SPAC4H3.13,SPBC28F2.10C,SPCC594.05C,SPAC2F3.15,SPAC5D6.05,SPBC2D10.06,SPCC1494.10,SPAC16C9.05,SPAC6G9.15C,SPBC776.02C,SPBC12D12.06,SPAC57A7.04C,SPBC13E7.08C,SPAC6F12.02,SPBC1685.08,SPCC126.04C,SPAC31A2.11C
GO:BP	cellular response to abiotic stimulus	GO:0071214	0.098591733	1.0061595	53	178	7	5044	SPAC23A1.19C,SPBC409.07C,SPBC30D10.10C,SPBC887.10,SPAC1A6.04C,SPAC644.06C,SPCC74.03C
GO:BP	cellular response to environmental stimulus	GO:0104004	0.098591733	1.0061595	53	178	7	5044	SPAC23A1.19C,SPBC409.07C,SPBC30D10.10C,SPBC887.10,SPAC1A6.04C,SPAC644.06C,SPCC74.03C
GO:CC	nucleotide-activated protein kinase complex	GO:0031588	0.016045245	1.794653635	3	178	3	5075	SPAC1556.08C,SPCC1919.03C,SPCC74.03C
KEGG	Mitophagy - yeast	KEGG:04139	0.048291622	1.316128207	29	54	5	1959	SPBC543.07,SPBC409.07C,SPBC11G11.01,SPBC30D10.10C,SPBC887.10

Supplymentary table 6: Positive genetic interactions for *btn1(102-208del)* vs *ade6*Δ control

source	term_name	term_id	adjusted_p_value	negative_log10_of_adjusted_p_value	term_size	query_size	intersection_size	effective_domain_size	intersections
KEGG	Autophagy - other	KEGG:04136	0.043925956	1.357278781	23	40	4	1959	SPBC3B9.06C,SPBP8B7.24C,SPBC31E1.01C,SPAC19B12.08
KEGG	Pyruvate metabolism	KEGG:00620	0.053914407	1.268295163	29	40	4	1959	SPAC1952.09C,SPBC12C2.12C,SPAC186.08C,SPBC3E7.16C

Supplymentary table 7: Negative genetic interactions unique for *btn1(102-208del)*

source	term_name	term_id	adjusted_p_value	negative_log10_of_adj	term_size	query_size	intersection_size	effective_domain_size	intersections
KEGG	Mitophagy - yeast	KEGG:04139	0.07303843	1.13644859	29	20	3	1959	SPBC543.07,SPBC887.10,SPBC11G11.01

Supplymentary table 8: Positive genetic interactions unique for *btn1(102-208del)*

source	term_name	term_id	adjusted_p_value	negative_log10_of_adj	term_size	query_size	intersection_size	effective_domain_size	intersections
GO:MF	anion transmembrane transporter activity	GO:0008509	0.043134765	1.365172562	79	55	6	5032	SPBC1271.09,SPCPB1C11.02,SPBC839.11C,SPCC1827.07C,SPBC1683.12,SPAC17G6.15C
GO:MF	protein kinase activity	GO:0004672	0.059276295	1.227118952	135	55	7	5032	SPAC222.07C,SPBC8D2.01,SPBC16E9.17C,SPAC23C4.12,SPBC6B1.02,SPCC18B5.11C,SPBC8D2.19
GO:MF	organic anion transmembrane transporter activity	GO:0008514	0.059276295	1.227118952	68	55	5	5032	SPBC1271.09,SPCPB1C11.02,SPBC839.11C,SPBC1683.12,SPAC17G6.15C
GO:MF	guanosine binding	GO:1905108	0.075827206	1.120174947	1	55	1	5032	SPCC18.09C
GO:MF	hydrolase activity, acting on acid carbon-carbon bonds	GO:0016822	0.075827206	1.120174947	1	55	1	5032	SPBC21C3.09C
GO:MF	hydrolase activity, acting on acid carbon-carbon bonds, in ketonic substances	GO:0016823	0.075827206	1.120174947	1	55	1	5032	SPBC21C3.09C
GO:MF	acetylpyruvate hydrolase activity	GO:0018773	0.075827206	1.120174947	1	55	1	5032	SPBC21C3.09C
GO:MF	GMP binding	GO:0019002	0.075827206	1.120174947	1	55	1	5032	SPCC18.09C
GO:MF	DNA 5'-adenosine monophosphate hydrolase activity	GO:0033699	0.075827206	1.120174947	1	55	1	5032	SPCC18.09C
GO:MF	propionyl-CoA:succinate CoA-transferase activity	GO:0043821	0.075827206	1.120174947	1	55	1	5032	SPAC1952.09C
GO:MF	acylpyruvate hydrolase activity	GO:0047621	0.075827206	1.120174947	1	55	1	5032	SPBC21C3.09C
GO:MF	L-pipecolate oxidase activity	GO:0050031	0.075827206	1.120174947	1	55	1	5032	SPBC354.15
GO:MF	proline oxidase activity	GO:0051699	0.075827206	1.120174947	1	55	1	5032	SPBC354.15
GO:MF	1-phosphatidylinositol 4-kinase activator activity	GO:0098744	0.075827206	1.120174947	1	55	1	5032	SPAC18B11.04
GO:MF	DNA-3'-diphospho-5'-guanosine diphosphatase	GO:0120108	0.075827206	1.120174947	1	55	1	5032	SPCC18.09C
GO:MF	trialkylsulfonium hydrolase activity	GO:0016802	0.075827206	1.120174947	1	55	1	5032	SPBC8D2.18C
GO:MF	electron transporter, transferring electrons within CoQH2-cytochrome c reductase complex activity	GO:0045153	0.075827206	1.120174947	1	55	1	5032	SPBC29A3.18

GO:MF	imidazoleglycerol-phosphate synthase activity	GO:0000107	0.075827206	1.120174947	1	55	1	5032	SPBC418.01C
GO:MF	kinase activity	GO:0016301	0.075827206	1.120174947	218	55	7	5032	SPAC222.07C,SPBC8D2.01,SPBC16E9.17C,SPAC23C4.12,SPBC6B1.02,SPCC18B5.11C,SPBC8D2.19
GO:MF	glycerophosphodiester transmembrane transporter activity	GO:0001406	0.075827206	1.120174947	1	55	1	5032	SPBC1271.09
GO:MF	adenosylhomocysteinase activity	GO:0004013	0.075827206	1.120174947	1	55	1	5032	SPBC8D2.18C
GO:MF	lactoylglutathione lyase activity	GO:0004462	0.075827206	1.120174947	1	55	1	5032	SPBC12C2.12C
GO:MF	protein serine/threonine kinase activity	GO:0004674	0.075827206	1.120174947	123	55	6	5032	SPAC222.07C,SPBC8D2.01,SPAC23C4.12,SPBC6B1.02,SPCC18B5.11C,SPBC8D2.19
GO:MF	cyclin-dependent protein serine/threonine kinase inhibitor activity	GO:0004861	0.075827206	1.120174947	1	55	1	5032	SPBC32F12.09
GO:MF	transporter activity	GO:0005215	0.075827206	1.120174947	334	55	9	5032	SPBC1271.09,SPCPB1C11.02,SPBC839.11C,SPBP26C9.03C,SPAC22G7.02,SPCC1827.07C,SPBC1683.12,SPAC17G6.15C,SPAC17A2.14
GO:MF	UDP-glucose transmembrane transporter activity	GO:0005460	0.075827206	1.120174947	1	55	1	5032	SPBC839.11C
GO:MF	CoA-transferase activity	GO:0008410	0.075827206	1.120174947	1	55	1	5032	SPAC1952.09C
GO:MF	phosphatidylethanolamine binding	GO:0008429	0.075827206	1.120174947	1	55	1	5032	SPBP8B7.24C
GO:MF	acetate CoA-transferase activity	GO:0008775	0.075827206	1.120174947	1	55	1	5032	SPAC1952.09C
GO:MF	ion transmembrane transporter activity	GO:0015075	0.075827206	1.120174947	204	55	8	5032	SPBC1271.09,SPCPB1C11.02,SPBC839.11C,SPBP26C9.03C,SPCC1827.07C,SPBC1683.12,SPAC17G6.15C,SPAC17A2.14
GO:MF	phosphate ion transmembrane transporter activity	GO:0015114	0.075827206	1.120174947	1	55	1	5032	SPCC1827.07C
GO:MF	phosphotransferase activity, alcohol group as acceptor	GO:0016773	0.075827206	1.120174947	189	55	7	5032	SPAC222.07C,SPBC8D2.01,SPBC16E9.17C,SPAC23C4.12,SPBC6B1.02,SPCC18B5.11C,SPBC8D2.19
GO:MF	transmembrane transporter activity	GO:0022857	0.087900733	1.056007502	292	55	8	5032	SPBC1271.09,SPCPB1C11.02,SPBC839.11C,SPBP26C9.03C,SPCC1827.07C,SPBC1683.12,SPAC17G6.15C,SPAC17A2.14
GO:MF	kinase regulator activity	GO:0019207	0.087900733	1.056007502	46	55	3	5032	SPBC32F12.09,SPAC18B11.04,SPBC16E9.17C
GO:MF	inorganic molecular entity transmembrane transporter activity	GO:0015318	0.098945067	1.004605854	188	55	6	5032	SPCPB1C11.02,SPBP26C9.03C,SPCC1827.07C,SPBC1683.12,SPAC17G6.15C,SPAC17A2.14
GO:MF	cyclin-dependent protein serine/threonine kinase regulator activity	GO:0016538	0.098945067	1.004605854	18	55	2	5032	SPBC32F12.09,SPBC16E9.17C

GO:BP	ion transmembrane transport	GO:0034220	0.036590294	1.436634099	239	55	11	5044	SPBC1271.09,SPCPB1C11.02,SPAC18B11.04,SPBC839.11C,SPBP26C9.03C,SPBC29A3.18,SPCC1827.07C,SPBC1683.12,SPAC1751.01C,SPAC17G6.15C,SPAC17A2.14
GO:BP	anion transmembrane transport	GO:0098656	0.041191379	1.385193674	100	55	7	5044	SPBC1271.09,SPCPB1C11.02,SPBC839.11C,SPCC1827.07C,SPBC1683.12,SPAC1751.01C,SPAC17G6.15C
GO:BP	ion transport	GO:0006811	0.049701107	1.30363394	279	55	11	5044	SPBC1271.09,SPCPB1C11.02,SPAC18B11.04,SPBC839.11C,SPBP26C9.03C,SPBC29A3.18,SPCC1827.07C,SPBC1683.12,SPAC1751.01C,SPAC17G6.15C,SPAC17A2.14
