# Supplementary material for: Global network analysis in Schizosaccharomyces pombe reveals three distinct consequences of the common 1-kb deletion causing juvenile CLN3 disease
Source: Sci Rep. 2021 Mar 18;11:6332. doi: 10.1038/s41598-021-85471-4 (PMC7973434; doi:10.1038/s41598-021-85471-4)
Supplement: Supplementary file 7 — S7: Supplementary Tables 7. [file 41598_2021_85471_MOESM7_ESM.pdf]

Global network analysis in *Schizosaccharomyces pombe* reveals three distinct consequences of the common 1-kb deletion causing juvenile CLN3 disease

Christopher J. Minnis[1,2], StJohn Townsend[3,4], Julia Petschnigg[1], Elisa Tinelli[1], Jürg Bähler[3], Claire Russell[2], Sara E. Mole[1]

[1]*MRC Laboratory for Molecular Cell Biology and Great Ormond Street Institute of Child Health, University College London, London WC1E 6BT, UK*
[2]*Dept. Comparative Biomedical Sciences, Royal Veterinary College, Royal College Street, London NW1 0TU, UK*
[3]*Institute of Healthy Ageing, Department of Genetics, Evolution and Environment, University College London, London WC1E 6BT, UK*
[4]*The Molecular Biology of Metabolism Laboratory, The Francis Crick Institute, London, NW1 1AT, United Kingdom*

*Corresponding author: christopher.minnis.15@ucl.ac.uk

Supplementary table 1 : Common loss of positive genetic interactions between *btn1*Δ and *btn1(102-208del)*

Systematic ID	Gene name	Product description	Positive Positive Max P-value
SPAC17G6.04c	cpp1	protein farnesyltransferase beta subunit Cpp1	4.53E-05
SPAC1834.05	alg9	mannosyltransferase complex subunit Alg9 (predicted)	1.17E-02
SPAC19B12.11c	bud20	zinc finger ribosome biogenesis protein Bud20 (predicted)	4.42E-02
SPAC23A1.03	apt1	adenine phosphoribosyltransferase (APRT) Apt1	4.36E-02
SPAC323.05c	mtq2	eRF1 methyltransferase Mtq2 (predicted)	1.84E-02
SPAC6G9.12	cfr1	exomer complex BRCT domain subunit Cfr1	1.57E-02
SPBC1734.12c	alg12	dolichyl pyrophosphate Man7GlcNAc2 alpha-1,6-mannosyltransferase Alg12 (predicted)	8.95E-03
SPBC887.17	SPBC887.17	nucleobase transmembrane transporter (predicted)	2.02E-02
SPBC9B6.03	SPBC9B6.03	zf-FYVE type zinc finger protein, involved in endosomal transport	2.68E-02
SPBP4H10.09	rsv1	transcription factor Rsv1	1.04E-02
SPCC1235.03	cue2	no go decay endonuclease Cue2	1.51E-02
SPCC320.06	SPCC320.06	conserved fungal protein	6.11E-04
SPCC330.11	btb1	BTB/POZ domain protein Btb1	1.57E-02
SPCC330.14c	rpl2402	60S ribosomal protein L24 (predicted)	3.43E-02
SPCC594.06c	vsl1	vacuolar SNARE Vsl1/Vam7	2.48E-02
SPCC736.09c	tfx1	TRAX	3.78E-02
SPCC757.02c	SPCC757.02c	dehydrogenase (predicted)	3.42E-02
SPCC794.15	SPCC794.15	Schizosaccharomyces specific protein	1.25E-02

Supplementary table 2 : Common loss of negative genetic interactions between *btn1*Δ and *btn1(102-208del)*

Systematic ID	Gene name	Product description	Negative Negative Max P-value
SPBC2F12.15c	pfa3	palmitoyltransferase Pfa3 (predicted)	3.72E-14
SPBC1271.12	kes1	sterol transfer protein Kes1 (predicted)	1.21E-05
SPAC11G7.01	mtl2	plasma membrane-associated serine-rich cell wall sensor Mtl2	9.06E-04
SPCP1E11.05c	are2	acyl-coA-sterol acyltransferase Are2	9.11E-04
SPBC25H2.16c	gga22	Golgi localized Arf binding gamma-adaptin ortholog Gga22	1.02E-03
SPAC4H3.13	pcc1	EKC/KEOPS complex subunit Pcc1 (predicted)	2.69E-03
SPAC4G8.10	gos1	SNARE Gos1 (predicted)	2.87E-03
SPCC126.15c	sec65	signal recognition particle subunit Sec65 (predicted)	3.02E-03
SPBC3F6.01c	SPBC3F6.01c	TPR repeat serine/threonine protein phosphatase (predicted)	3.74E-03
SPBP8B7.05c	nce103	carbonic anhydrase (predicted)	3.91E-03
SPBC26H8.14c	cox17	mitochondrial copper chaperone for cytochrome c oxidase Cox17 (predicted)	5.50E-03
SPBC646.13	sds23	PP2A-type phosphatase inhibitor Sds23/Moc1	5.85E-03
SPBC11B10.07c	ivn1	plasma membrane phospholipid-translocating ATPase complex Lem3 family subunit Ivn1 (predicted)	6.59E-03

SPAC458.05	pik3	phosphatidylinositol 3-kinase Pik3	7.11E-03
SPBC2D10.06	rep1	MBF transcription factor activator Rep1	7.11E-03
SPCC576.13	swc5	Swr1 complex subunit Swc5	8.48E-03
SPBC337.09	erg28	Erg28 protein (predicted)	9.82E-03
SPBC26H8.08c	grn1	GTPase Grn1	1.04E-02
SPBC23E6.01c	cxr1	splicing factor Cxr1	1.11E-02
SPBC1734.05c	spf31	DNAJ protein, splicing factor Spf31 (predicted)	1.25E-02
SPBC336.03	efc25	Ras1 GEF Efc25	1.48E-02
SPAC3H8.09c	nab3	poly(A) binding protein Nab3 (predicted)	1.68E-02
SPBC11C11.02	imp2	F-BAR domain protein Imp2	1.69E-02
SPAC1486.08	cox16	mitochondrial copper chaperone for cytochrome c oxidase Cox16 (predicted)	2.21E-02
SPBC25H2.08c	mrs2	mitochondrial inner membrane magnesium ion transmembrane transporter Mrs2 (predicted)	2.36E-02
SPAC4C5.04	rad31	SUMO activating enzyme E1-type Rad31	2.41E-02
SPBC13E7.08c	leo1	RNA polymerase II associated Paf1 complex subunit Leo1	2.48E-02
SPAC22H12.05c	fsc1	fasciclin domain protein Fsc1	2.81E-02
SPBC16E9.09c	erp5	COPII vesicle coat component Erp5/Erp6 (predicted)	3.25E-02
SPAC922.05c	SPAC922.05c	transmembrane transporter (predicted)	3.73E-02
SPAC4C5.02c	ryh1	GTPase Ryh1	3.93E-02
SPCC1494.08c	SPCC1494.08c	cortical variant C2 domain protein, human FAM102A and FAM102B ortholog, implicated in signalling or endocytosis	3.93E-02
SPAC25B8.06c	dia4	mitochondrial serine-tRNA ligase (predicted)	3.99E-02
SPAC3A11.08	pcu4	cullin 4	4.02E-02
SPAC513.03	mfm2	M-factor precursor Mfm2	4.35E-02
