# Supplementary material for: Global network analysis in Schizosaccharomyces pombe reveals three distinct consequences of the common 1-kb deletion causing juvenile CLN3 disease
Source: Sci Rep. 2021 Mar 18;11:6332. doi: 10.1038/s41598-021-85471-4 (PMC7973434; doi:10.1038/s41598-021-85471-4)
Supplement: Supplementary file 8 — S8: Supplementary Tables 8. [file 41598_2021_85471_MOESM8_ESM.pdf]

# Global network analysis in *Schizosaccharomyces pombe* reveals three distinct consequences of the common 1-kb deletion causing juvenile CLN3 disease

Christopher J. Minnis[1,2], StJohn Townsend[3,4], Julia Petschnigg[1], Elisa Tinelli[1], Jürg Bähler[3], Claire Russell[2], Sara E. Mole[1]

[1]*MRC Laboratory for Molecular Cell Biology and Great Ormond Street Institute of Child Health, University College London, London WC1E 6BT, UK*
[2]*Dept. Comparative Biomedical Sciences, Royal Veterinary College, Royal College Street, London NW1 0TU, UK*
[3]*Institute of Healthy Ageing, Department of Genetics, Evolution and Environment, University College London, London WC1E 6BT, UK*
[4]*The Molecular Biology of Metabolism Laboratory, The Francis Crick Institute, London, NW1 1AT, United Kingdom*

*Corresponding author: christopher.minnis.15@ucl.ac.uk

Supplementary table 1 : Residual function in *btn1(102-208del)* rescuing positive interactions of *btn1Δ*

| Systematic ID | Gene name | Product description | Positive Negative Max P-value |
|---|---|---|---|
| SPAC3G9.03 | rpl2301 | 60S ribosomal protein L23 | 5.60E-04 |
| SPCC1393.08 | fil1 | transcription factor, zf-GATA type | 7.10E-04 |
| SPCC18B5.10c | tex1 | TREX complex subunit Tex1 (predicted) | 7.71E-04 |
| SPBC19F8.08 | rps401 | 40S ribosomal protein S4 (predicted) | 3.79E-03 |
| SPAPB2B4.02 | grx5 | mitochondrial [2Fe-2S] cluster assembly and transfer glutaredoxin Grx5 | 4.75E-03 |
| SPBC1A4.05 | blt1 | ubiquitin domain-like protein Blt1 | 6.10E-03 |
| SPBP16F5.05c | yar1 | ribosome biogenesis protein Yar1 (predicted) | 7.11E-03 |
| SPAC13G6.10c | asl1 | cell wall protein Asl1, predicted O-glucosyl hydrolase | 1.55E-02 |
| SPBC3D6.04c | mad1 | mitotic spindle checkpoint protein Mad1 | 1.63E-02 |
| SPAPJ696.01c | vps17 | retromer complex subunit Vps17 | 2.24E-02 |
| SPCC1739.14 | npp106 | nucleoporin Npp106 | 2.30E-02 |
| SPAC1F3.03 | sro7 | Lgl family protein Sro7 (predicted) | 3.10E-02 |
| SPBC18H10.19 | vps38 | phophatidylinositol 3-kinase complex subunit Vps38 | 4.39E-02 |

Supplementary table 2 : Residual function in *btn1(102-208del)* rescuing positive interactions of *btn1Δ*

| Systematic ID | Gene name | Product description | Negative Positive Max P-value |
|---|---|---|---|
| SPAC11D3.04c | SPAC11D3.04c | polyketide cyclase SnoaL-like domain protein | 3.46E-03 |
| SPBC1709.12 | rid1 | GTPase binding protein Rid1 (predicted) | 2.29E-02 |
| SPCC70.08c | SPCC70.08c | methyltransferase (predicted) | 3.73E-02 |
