# Supplementary material for: Global network analysis in Schizosaccharomyces pombe reveals three distinct consequences of the common 1-kb deletion causing juvenile CLN3 disease
Source: Sci Rep. 2021 Mar 18;11:6332. doi: 10.1038/s41598-021-85471-4 (PMC7973434; doi:10.1038/s41598-021-85471-4)
Supplement: Supplementary file 9 — S9: Supplementary Tables 9. [file 41598_2021_85471_MOESM9_ESM.pdf]

Global network analysis in *Schizosaccharomyces pombe* reveals three distinct consequences of the common 1-kb deletion causing juvenile CLN3 disease

Christopher J. Minnis[1,2], StJohn Townsend[3,4], Julia Petschnigg[1], Elisa Tinelli[1], Jürg Bähler[3], Claire Russell[2], Sara E. Mole[1]

[1]*MRC Laboratory for Molecular Cell Biology and Great Ormond Street Institute of Child Health, University College London, London WC1E 6BT, UK*
[2]*Dept. Comparative Biomedical Sciences, Royal Veterinary College, Royal College Street, London NW1 0TU, UK*
[3]*Institute of Healthy Ageing, Department of Genetics, Evolution and Environment, University College London, London WC1E 6BT, UK*
[4]*The Molecular Biology of Metabolism Laboratory, The Francis Crick Institute, London, NW1 1AT, United Kingdom*

*Corresponding author: christopher.minnis.15@ucl.ac.uk

Supplementary table 1 : Increased senstivity in *btn1(102-208del)* mutant compared to *btn1Δ* positive interaction

Systematic ID	Gene name	Product description	Positive Positive Max P-value
SPCC736.09c	tfx1	TRAX	3.78E-02

Supplementary table 2 : Increased sensitivity in *btn1(102-208del)* mutant compared to *btn1Δ* negative interaction

Systematic ID	Gene name	Product description	Negative Negative Max P-value
SPBP8B7.05c	nce103	carbonic anhydrase (predicted)	3.91E-03
SPBC25H2.16c	gga22	Golgi localized Arf binding gamma-adaptin ortholog Gga22	1.50E-02
