# Supplementary material for: Global network analysis in Schizosaccharomyces pombe reveals three distinct consequences of the common 1-kb deletion causing juvenile CLN3 disease
Source: Sci Rep. 2021 Mar 18;11:6332. doi: 10.1038/s41598-021-85471-4 (PMC7973434; doi:10.1038/s41598-021-85471-4)
Supplement: Supplementary file 10 — S10: Supplementary Tables 10. [file 41598_2021_85471_MOESM10_ESM.pdf]

Global network analysis in *Schizosaccharomyces pombe* reveals three distinct consequences of the common 1-kb deletion causing juvenile CLN3 disease

Christopher J. Minnis[1,2], StJohn Townsend[3,4], Julia Petschnigg[1], Elisa Tinelli[1], Jürg Bähler[3], Claire Russell[2], Sara E. Mole[1]

[1]*MRC Laboratory for Molecular Cell Biology and Great Ormond Street Institute of Child Health, University College London, London WC1E 6BT, UK*

[2]*Dept. Comparative Biomedical Sciences, Royal Veterinary College, Royal College Street, London NW1 0TU, UK*

[3]*Institute of Healthy Ageing, Department of Genetics, Evolution and Environment, University College London, London WC1E 6BT, UK*

[4]*The Molecular Biology of Metabolism Laboratory, The Francis Crick Institute, London, NW1 1AT, United Kingdom*

*Corresponding author: christopher.minnis.15@ucl.ac.uk

Supplementary table 1 : Unique negative interactions for the *btn1(102-208del)* mutation

Systematic ID	Gene name	Product description	Colony Size Difference *btn1* $^{102-208del}$ vs *btn1Δ*	t	P Value	Adjusted P Value	Colony Size Difference *btn1Δ* vs *ade6Δ*
SPBC1D7.03	clg1	cyclin-like protein involved in autophagy Clg1 (predicted)	-0.4	-5.79	5.62E-07	3.65E-04	-0.07
SPBC530.08	SPBC530.08	membrane-tethered transcription factor (predicted)	-0.42	-5.53	1.42E-06	5.26E-04	0.01
SPAC823.10c	hem25	mitochondrial carrier, glycine Hem25 (predicted)	-0.56	-5.4	2.15E-06	5.60E-04	0.02
SPBC4B4.03	rsc1	RSC complex subunit Rsc1	-0.53	-5.18	4.61E-06	8.57E-04	0.01
SPCC1739.10	mug33	Tea1-interacting protein involved in exocytosis	-0.52	-4.76	1.91E-05	2.16E-03	0.06
SPBC12C2.04	SPBC12C2.04	NAD binding dehydrogenase family protein	-0.37	-4.71	2.23E-05	2.23E-03	0.05
SPAC19B12.10	sst2	human AMSH/STAMBP protein homolog, ubiquitin specific-protease	-0.64	-4.72	2.56E-05	2.30E-03	0.07
SPBC543.07	pek1	MAP kinase kinase Pek1	-0.38	-4.69	2.42E-05	2.30E-03	-0.1
SPBC2D10.13	est1	telomerase regulator Est1	-0.4	-4.65	2.70E-05	2.34E-03	-0.01
SPCC16C4.10	SPCC16C4.10	6-phosphogluconolactonase (predicted)	-0.29	-4.56	3.67E-05	2.65E-03	-0.03
SPCC594.05c	spf1	Set1C PHD finger subunit Spf1	-0.35	-4.57	3.52E-05	2.65E-03	0.02
SPAC26H5.10c	tif51	translation elongation and termination factor eIF5A (predicted)	-0.4	-4.43	5.58E-05	3.82E-03	0.01
SPBC1105.10	rav1	RAVE complex subunit Rav1	-0.3	-4.33	7.84E-05	4.63E-03	0
SPBC839.15c	tef103	translation elongation factor EF-1 alpha Ef1a-c	-0.57	-4.24	1.04E-04	5.76E-03	0.09
SPBC3H7.15	hhp1	serine/threonine protein kinase Hhp1	-0.28	-4.21	1.14E-04	5.88E-03	0
SPBPB2B2.13	gal1	galactokinase Gal1	-0.38	-4.22	1.11E-04	5.88E-03	0.03
SPBC887.10	mcs4	response regulator Mcs4	-0.65	-4.14	1.43E-04	6.85E-03	0.05
SPAC8E11.05c	SPAC8E11.05c	DUF5102 family conserved fungal protein, associated with clathrin coated vesicles (predicted)	-0.36	-3.94	2.69E-04	1.11E-02	0.09
SPAC19G12.13c	poz1	shelterin complex subunit Poz1	-0.47	-3.9	3.10E-04	1.22E-02	-0.07
SPBC1A4.04	SPBC1A4.04	Schizosaccharomyces specific protein	-0.35	-3.89	3.15E-04	1.22E-02	0.02
SPCC18.17c	SPCC18.17c	proteasome assembly chaperone (predicted)	-0.3	-3.86	3.44E-04	1.27E-02	0.02
SPAC2F7.04	med1	mediator complex subunit Med1	-0.37	-3.85	3.58E-04	1.29E-02	0.1
SPAC8F11.02c	dph3	diphthamide biosynthesis protein Dph3 (predicted)	-0.53	-3.82	3.90E-04	1.32E-02	0.04
SPCC794.11c	ent3	ENTH/VHS domain protein Ent3 (predicted)	-0.34	-3.8	4.21E-04	1.37E-02	0.01
SPAC16E8.05c	SPAC16E8.05c	Schizosaccharomyces specific protein Mde1	-0.39	-3.7	5.62E-04	1.68E-02	0.08
SPBC32F12.01c	css1	inositol phosphosphingolipid phospholipase C, Css1	-0.39	-3.65	6.53E-04	1.85E-02	0
SPBC17A3.09c	aim22	lipoate-protein ligase A (predicted)	-0.38	-3.64	6.73E-04	1.86E-02	-0.09
SPBC1604.02c	ppr1	mitochondrial PPR repeat protein Ppr1	-0.31	-3.59	7.86E-04	2.10E-02	-0.07
SPBC409.11	meu18	Schizosaccharomyces specific protein Meu18	-0.28	-3.59	7.95E-04	2.10E-02	0.03
SPBC27B12.03c	erg32	C-5 sterol desaturase Erg32	-0.38	-3.55	8.85E-04	2.21E-02	-0.03
SPAC2F7.17	mrf1	mitochondrial translation release factor (predicted)	-0.23	-3.52	9.78E-04	2.29E-02	-0.04
SPBC3E7.01	fab1	1-phosphatidylinositol-3-phosphate 5-kinase Fab1	-0.42	-3.53	9.51E-04	2.29E-02	0
SPBC365.10	arp5	Ino80 complex actin-like protein Arp5	-0.33	-3.49	1.06E-03	2.41E-02	0.08
SPBC21B10.06c	inp2	myosin binding vezatin family protein involved in peroxisome inheritance Inp2 (predicted)	-0.33	-3.42	1.30E-03	2.57E-02	0.06
SPBC3E7.10	fma1	methionine aminopeptidase Fma1 (predicted)	-0.32	-3.41	1.36E-03	2.60E-02	0.04
SPAC16C9.05	cph1	Clr6 histone deacetylase associated PHD finger protein Cph1	-0.48	-3.41	1.42E-03	2.68E-02	-0.08
SPCC794.03	SPCC794.03	amino acid transmembrane transporter (predicted)	-0.32	-3.38	1.48E-03	2.75E-02	0.08
SPCC16C4.20c	hap2	Ino80 complex, HMG box protein Hap2	-0.39	-3.32	1.77E-03	3.10E-02	0.08
SPBC21B10.03c	ath1	ataxin-2 homolog	-0.34	-3.26	2.07E-03	3.49E-02	-0.04

SPAC222.05c	mss1	mitochondrial tRNA wobble uridine modification GTPase Mss1 (predicted)	-0.22	-3.26	2.10E-03	3.51E-02	-0.05
SPAC1A6.04c	plb1	phospholipase B homolog Plb1	-0.42	-3.23	2.26E-03	3.65E-02	-0.04
SPBC11G11.01	fis1	mitochondrial fission protein Fis1 (predicted)	-0.28	-3.2	2.49E-03	3.86E-02	-0.1
SPAC1039.08	SPAC1039.08	serine acetyltransferase (predicted)	-0.41	-3.17	2.66E-03	4.10E-02	0
SPBC106.16	pre6	20S proteasome complex subunit alpha 4 Pre6	-0.52	-3.16	2.79E-03	4.24E-02	-0.02
SPBC4F6.06	kin1	microtubule affinity-regulating kinase Kin1	-0.3	-3.14	2.93E-03	4.36E-02	-0.06
SPAC1B3.07c	vps28	ESCRT I complex subunit Vps28	-0.23	-3.13	3.05E-03	4.45E-02	0.06
SPAC31G5.04	lys12	homoisocitrate dehydrogenase Lys12	-0.21	-3.12	3.14E-03	4.51E-02	-0.03
SPCC1919.05	ski3	Ski complex TPR repeat subunit Ski3 (predicted)	-0.22	-3.1	3.32E-03	4.71E-02	-0.01
SPAC212.03	SPAC212.03	hypothetical protein	-0.45	-3.1	3.38E-03	4.75E-02	-0.02
SPAP8A3.13c	SPAP8A3.13c	Vid24 family protein (predicted)	-0.25	-3.08	3.51E-03	4.86E-02	0
SPBCPT2R1.08c	tlh2	RecQ type DNA helicase Tlh1	-0.28	-3.09	3.50E-03	4.86E-02	0.04
SPAC8E11.02c	rad24	14-3-3 protein Rad24	-0.24	-3.06	3.66E-03	4.97E-02	0.05

Supplementary table 2 : Unique positive interactions for the *btn1(102-208del)* mutation

**Systematic ID**	**Gene name**	**Product description**	**Colony Size Difference *btn1*$^{102\text{-}208del}$ vs *btn1Δ***	**t**	**P Value**	**Adjusted P Value**	**Colony Size Difference *btn1Δ* vs *ade6Δ***
SPBC211.06	gfh1	gamma tubulin complex subunit Gfh1	0.48	6.24	1.18E-07	1.02E-04	-0.03
SPBC32F12.09	rum1	CDK inhibitor Rum1	0.42	5.61	1.07E-06	5.22E-04	-0.08
SPBC418.01c	his4	imidazoleglycerol-phosphate synthase His4	0.46	5.42	2.04E-06	5.60E-04	0
SPBC1271.09	tgp1	plasma membrane glycerophosphodiester transmembrane transporter (predicted)	0.36	5.26	3.50E-06	7.58E-04	-0.04
SPCPB1C11.02	SPCPB1C11.02	amino acid transmembrane transporter (predicted)	0.35	4.97	9.43E-06	1.53E-03	0
SPAC18B11.04	ncs1	neuronal calcium sensor related protein Ncs1	0.34	4.86	1.37E-05	1.85E-03	0.02
SPBC4.06	SPBC4.06	acid phosphatase Fmp10 (predicted)	0.34	4.86	1.36E-05	1.85E-03	-0.03
SPBC839.11c	hut1	ER uridine diphosphate-glucose transmembrane transporter Hut1	0.36	4.79	1.73E-05	2.04E-03	0.01
SPAC222.07c	hri2	eIF2 alpha kinase Hri2	0.42	4.75	2.00E-05	2.17E-03	-0.03
SPBC3B9.06c	atg3	autophagy associated protein Atg3	0.47	4.57	3.63E-05	2.65E-03	-0.02
SPBC8D2.01	gsk31	serine/threonine protein kinase Gsk31 (predicted)	0.59	4.58	3.46E-05	2.65E-03	0.09
SPBP8B7.24c	atg8	autophagy associated protein Atg8	0.34	4.42	5.84E-05	3.89E-03	-0.02
SPBP26C9.03c	fet4	plasma membrane iron/zinc ion transmembrane transporter (predicted)	0.32	4.37	6.82E-05	4.33E-03	-0.01
SPBC16E9.17c	rem1	meiosis-specific cyclin Rem1	0.33	4.35	7.26E-05	4.50E-03	-0.08
SPBC12C2.12c	glo1	glyoxalase I	0.29	4.22	1.12E-04	5.88E-03	-0.01
SPAC3A11.05c	kms1	meiotic spindle pole body KASH domain protein Kms1	0.33	4.15	1.41E-04	6.85E-03	-0.07
SPAC23C4.12	hhp2	serine/threonine protein kinase Hhp2	0.36	4.11	1.58E-04	7.32E-03	-0.09
SPBC29A3.18	cyt1	cytochrome c1 Cyt1 (predicted)	0.25	3.94	2.75E-04	1.12E-02	-0.02
SPBC16E9.02c	SPBC16E9.02c	CUE domain protein, human TOLLIP ortholog	0.33	3.88	3.28E-04	1.24E-02	-0.06
SPBC18E5.07	aim21	barbed end F-actin assembly inhibitor	0.35	3.86	3.47E-04	1.27E-02	-0.03
SPBC6B1.02	ppk30	Ark1/Prk1 family protein kinase Ppk30	0.9	3.84	3.68E-04	1.29E-02	0.1
SPAC1039.03	SPAC1039.03	esterase/lipase, implicated in cellular detoxification (predicted)	0.24	3.8	4.15E-04	1.37E-02	-0.08
SPAC1952.09c	SPAC1952.09c	acetyl-CoA hydrolase (predicted)	0.27	3.79	4.31E-04	1.37E-02	0.07
SPBC354.15	fap1	L-pipecolate oxidase	0.3	3.79	4.32E-04	1.37E-02	0.08

SPBC19C2.02	pmt1	tRNA (cytosine-5-)-methyltransferase Pmt1	0.27	3.74	5.01E-04	1.53E-02	0.02
SPAC1F7.12	yak3	aldose reductase ARK13 family YakC, implicated in cellular detoxification from family members	0.33	3.69	5.85E-04	1.73E-02	-0.02
SPAC22G7.02	kap111	karyopherin/importin beta family nuclear import signal receptor Kap111 (predicted)	0.33	3.66	7.00E-04	1.91E-02	-0.06
SPBC1198.07c	SPBC1198.07c	mannan endo-1,6-alpha-mannosidase (predicted)	0.24	3.58	8.22E-04	2.10E-02	-0.02
SPAC1420.01c	SPAC1420.01c	GATA-like domain protein (predicted)	0.48	3.56	8.60E-04	2.17E-02	0.01
SPBC21D10.07	cmc1	copper-binding protein of the mitochondrial intermembrane space Cmc1 (predicted)	0.35	3.52	9.87E-04	2.29E-02	0.06
SPBC839.07	ibp1	Cdc25 family phosphatase Ibp1, unknown role, implicated in DNA replication	0.69	3.52	9.75E-04	2.29E-02	0.01
SPCC18.09c	hnt3	aprataxin Hnt3	0.44	3.51	1.01E-03	2.32E-02	0.08
SPCC1827.07c	SPCC1827.07c	SPX/EXS domain protein (predicted)	0.2	3.49	1.05E-03	2.40E-02	-0.06
SPBC1683.12	SPBC1683.12	carboxylic acid transmembrane transporter (predicted)	0.38	3.43	1.29E-03	2.57E-02	0.01
SPBC713.07c	SPBC713.07c	vacuolar polyphosphatase (predicted)	0.28	3.43	1.28E-03	2.57E-02	-0.06
SPBC83.19c	SPBC83.19c	Schizosaccharomyces pombe specific protein	0.23	3.47	1.22E-03	2.57E-02	0.02
SPAPB8E5.04c	npc2	Niemann-Pick disease type C2 protein hE1 homolog Npc2 (predicted)	0.24	3.41	1.36E-03	2.60E-02	0
SPBC800.12c	SPBC800.12c	ubiquitin family protein (predicted)	0.28	3.41	1.36E-03	2.60E-02	-0.04
SPAC890.07c	rmt1	type I protein arginine N-methyltransferase Rmt1	0.31	3.35	1.72E-03	3.08E-02	-0.09
SPCC18B5.11c	cds1	replication checkpoint kinase Cds1	0.37	3.31	1.82E-03	3.16E-02	0.01
SPAC18B11.08c	SPAC18B11.08c	UPF0139 family conserved fungal ER membrane protein	0.3	3.28	1.95E-03	3.34E-02	-0.01
SPAC1751.01c	gti1	gluconate transmembrane transporter inducer Gti1	0.46	3.26	2.11E-03	3.51E-02	0.07
SPBC342.03	gas4	spore wall 1,3-beta-glucanosyltransferase Gas4	0.21	3.25	2.12E-03	3.51E-02	-0.03
SPBC83.05	SPBC83.05	mitochondrial RNA-binding protein (predicted)	0.64	3.22	2.31E-03	3.70E-02	0.1
SPAC17G6.15c	fsf1	mitochondrial carrier, serine Fsf1 (predicted)	0.2	3.2	2.45E-03	3.86E-02	0.03
SPBC11C11.06c	SPBC11C11.06c	Schizosaccharomyces specific protein	0.25	3.2	2.49E-03	3.86E-02	0.09
SPBC16A3.14	SPBC16A3.14	superoxide dismutase, mitochondrial ribosomal protein subunit (predicted)	0.2	3.15	2.84E-03	4.28E-02	-0.08
SPBC691.03c	apl3	AP-2 adaptor complex alpha subunit Alp3	0.34	3.15	2.85E-03	4.28E-02	0.03
SPBC1773.16c	SPBC1773.16c	transcription factor, zf-fungal binuclear cluster type(predicted)	0.27	3.13	3.00E-03	4.44E-02	-0.05
SPAC17A2.14	mnr2	vacuolar CorA family magnesium ion transmembrane transporter Mnr2	0.25	3.14	3.06E-03	4.45E-02	-0.01
SPBC8D2.19	mde3	serine/threonine protein kinase, meiotic Mde3	0.69	3.12	3.06E-03	4.45E-02	-0.06
SPBC365.11	grp2	Golgi GRIP domain protein Grp2 (predicted)	0.2	3.11	3.17E-03	4.53E-02	0
SPAC56F8.14c	mug115	Schizosaccharomyces pombe specific protein Mug115	0.36	3.08	3.51E-03	4.86E-02	-0.02
SPBC8D2.18c	SPBC8D2.18c	adenosylhomocysteinase (predicted)	0.22	3.07	3.59E-03	4.94E-02	0.02
SPBC21C3.09c	oaa1	mitochondrial acylpyruvase Oaa1 (predicted)	0.32	3.06	3.71E-03	4.97E-02	0.06
