# Supplementary material for: Global network analysis in Schizosaccharomyces pombe reveals three distinct consequences of the common 1-kb deletion causing juvenile CLN3 disease
Source: Sci Rep. 2021 Mar 18;11:6332. doi: 10.1038/s41598-021-85471-4 (PMC7973434; doi:10.1038/s41598-021-85471-4)
Supplement: Supplementary file 11 — S11: Supplementary Table 11. [file 41598_2021_85471_MOESM11_ESM.pdf]

Global network analysis in *Schizosaccharomyces pombe* reveals three distinct consequences of the common 1-kb deletion causing juvenile CLN3 disease

Christopher J. Minnis[1,2], StJohn Townsend[3,4], Julia Petschnigg[1], Elisa Tinelli[1], Jürg Bähler[3], Claire Russell[2], Sara E. Mole[1]

[1]MRC Laboratory for Molecular Cell Biology and Great Ormond Street Institute of Child Health, University College London, London WC1E 6BT, UK
[2]Dept. Comparative Biomedical Sciences, Royal Veterinary College, Royal College Street, London NW1 0TU, UK
[3]Institute of Healthy Ageing, Department of Genetics, Evolution and Environment, University College London, London WC1E 6BT, UK
[4]The Molecular Biology of Metabolism Laboratory, The Francis Crick Institute, London, NW1 1AT, United Kingdom

*Corresponding author: christopher.minnis.15@ucl.ac.uk

Supplementary table 1 : Summary of MONDO disease terms and their corresponding genes for each btn1 mutant strains

					btn1(102-208del)				btn1(D363G)				btn1∆
**Disease terms**	**MONDO ID**	**# genes**	**Systematic ID**	**Gene name**	**Product description**	**# genes**	**Systematic ID**	**Gene name**	**Product description**	**# genes**	**Systematic ID**	**Gene name**	**Product description**
kidney disease	MONDO:0005240	16	SPAC23A1.03	apt1	adenine phosphoribosyltransferase (APRT) Apt1	4	SPAC2C4.07c	dis32	3'-5'-exoribonuclease activity Dis3L2	6	SPAC2C4.07c	dis32	3'-5'-exoribonuclease activity Dis3L2
			SPBC2D10.16	mhf1	CENP-S ortholog, FANCM-MHF complex subunit Mhf1		SPAC23A1.03	apt1	adenine phosphoribosyltransferase (APRT) Apt1		SPAC23A1.03	apt1	adenine phosphoribosyltransferase (APRT) Apt1
			SPCC16C4.09	sts5	cytoplasmic P body 3'-5'-exoribonuclease, Dis3L2-related (predicted)		SPAC25B8.06c	dia4	mitochondrial serine-tRNA ligase (predicted)		SPAC4H3.13	pcc1	EKC/KEOPS complex subunit Pcc1 (predicted)
			SPCC18B5.11c	cds1	DNA replication checkpoint kinase Cds1		SPBC21C3.11	ubx4	UBX domain protein Ubx4 (predicted)		SPACUNK4.10	gor1	glyoxylate reductase (predicted)
			SPAC4H3.13	pcc1	EKC/KEOPS complex subunit Pcc1 (predicted)						SPAC25B8.06c	dia4	mitochondrial serine-tRNA ligase (predicted)
			SPBPB2B2.13	gal1	galactokinase Gal1						SPBC21C3.11	ubx4	UBX domain protein Ubx4 (predicted)
			SPAC1805.15c	pub2	HECT-type ubiquitin-protein ligase E3 Pub2								
			SPBC16E9.11c	pub3	HECT-type ubiquitin-protein ligase E3 Pub3 (predicted)								
			SPBC24C6.08c	bhd1	Lst4-Lst7 complex subunit, folliculin Bhd1/Lst7								
			SPAC25B8.06c	dia4	mitochondrial serine-tRNA ligase (predicted)								
			SPAC6F12.06	rdi1	Rho GDP dissociation inhibitor Rdi1 (predicted)								
			SPBC4B4.03	rsc1	RSC complex subunit Rsc1								
			SPBC30D10.10c	tor1	serine/threonine protein kinase Tor1								
			SPAC823.05c	tlg2	SNARE Tlg2 (predicted)								
			SPCC364.02c	bis1	splicing factor Bis1								
			SPAC688.06c	slx4	structure-specific endonuclease subunit Slx4								
eye disease	MONDO:0005328	15	SPBC3E7.01	fab1	1-phosphatidylinositol-3-phosphate 5-kinase Fab1	6	SPAC3A11.08	pcu4	cullin 4	6	SPAC3A11.08	pcu4	cullin 4
			SPCC18.09c	hnt3	aprataxin Hnt3		SPBC651.03c	gyp10	GTPase activating protein Gyp10		SPCC1919.10c	myo52	myosin type V
			SPBC21B10.03c	ath1	ataxin-2 homolog		SPAC11G7.03	idh1	isocitrate dehydrogenase (NAD+) subunit 1 Idh1		SPAC1071.03c	sil1	nucleotide exchange factor for the ER lumenal Hsp70 chaperone, Sil1 (predicted)
			SPBC28E12.06c	lvs1	beige protein homolog Lvs1		SPCC1919.10c	myo52	myosin type V		SPAC823.03	ppk15	serine/threonine protein kinase Ppk15 (predicted)
			SPAC3A11.08	pcu4	cullin 4		SPAC23A1.19c	hrq1	RecQ type DNA helicase Hrq1 (predicted)		SPCP31B10.07	eft202	translation elongation factor 2 (EF-2) Eft2,B
			SPBPB2B2.13	gal1	galactokinase Gal1		SPAC23G3.08c	ubp7	ubiquitin C-terminal hydrolase Ubp7		SPAC23G3.08c	ubp7	ubiquitin C-terminal hydrolase Ubp7
			SPBC211.06	gfh1	gamma tubulin complex GPC4 subunit Gfh1								
			SPBC17A3.09c	aim22	lipoate-protein ligase A (predicted)								
			SPBC530.10c	anc1	mitochondrial carrier, ATP:ADP antiporter Anc1								
			SPBC119.06	sco1	mitochondrial copper chaperone for cytochrome c oxidase Sco1 (predicted)								
			SPAC1F3.10c	oct1	mitochondrial intermediate peptidase Oct1 (predicted)								
			SPBC543.09	yta12	mitochondrial m-AAA protease Yta12 (predicted)								
			SPAC23A1.19c	hrq1	RecQ type DNA helicase Hrq1 (predicted)								
			SPCC126.04c	sgf73	SAGA complex deubiquitinating submodule subunit Sgf73								
			SPBC776.02c	dis2	serine/threonine protein phosphatase PP1, Dis2								
inborn mitochondrial metabolism disorder	MONDO:0004069	13	SPBC3H7.03c	kgd1	oglutarate dehydrogenase (lipoamide) (e1 component of oxoglutarate dehydrogenase complex) (predi	4	SPAC14C4.14	atp1	F1-FO ATP synthase alpha subunit	3	SPAPB2B4.02	grx5	mitochondrial [2Fe-2S] cluster assembly and transfer glutaredoxin Grx5
			SPBC29A3.18	cyt1	cytochrome c1 Cyt1 (predicted)		SPAPB2B4.02	grx5	mitochondrial [2Fe-2S] cluster assembly and transfer glutaredoxin Grx5		SPAC25B8.06c	dia4	mitochondrial serine-tRNA ligase (predicted)
			SPBC17A3.09c	aim22	lipoate-protein ligase A (predicted)		SPBC1703.13c	SPBC1703.13c	mitochondrial carrier, inorganic phosphate (predicted)		SPBC30D10.13c	pdb1	pyruvate dehydrogenase e1 component beta subunit Pdb1
			SPBC530.10c	anc1	mitochondrial carrier, ATP:ADP antiporter Anc1		SPAC25B8.06c	dia4	mitochondrial serine-tRNA ligase (predicted)				
			SPAC823.10c	hem25	mitochondrial carrier, glycine Hem25 (predicted)								
			SPAC17G6.15c	fsf1	mitochondrial carrier, serine Fsf1 (predicted)								
			SPBC119.06	sco1	mitochondrial copper chaperone for cytochrome c oxidase Sco1 (predicted)								
			SPAC30C2.06c	dml1	mitochondrial inheritance GTPase, tubulin-like (predicted)								
			SPAC1F3.10c	oct1	mitochondrial intermediate peptidase Oct1 (predicted)								
			SPBC543.09	yta12	mitochondrial m-AAA protease Yta12 (predicted)								
			SPCC16C4.01	sif2	mitochondrial protein, involved in mitochondrial gene expression (predicted)								
			SPAC25B8.06c	dia4	mitochondrial serine-tRNA ligase (predicted)								
			SPAC222.05c	mss1	mitochondrial tRNA wobble uridine modification GTPase Mss1 (predicted)								
epilepsy	MONDO:0005027	12	SPBC3H7.03c	kgd1	oglutarate dehydrogenase (lipoamide) (e1 component of oxoglutarate dehydrogenase complex) (predi	6	SPCC757.07c	ctt1	catalase	7	SPAC3A11.08	pcu4	cullin 4
			SPBC1683.12	SPBC1683.12	carboxylic acid transmembrane transporter (predicted)		SPAC15A10.06	SPAC15A10.06	CPA1 sodium ion/proton antiporter (predicted)		SPAC1834.05	alg9	mannosyltransferase complex subunit Alg9 (predicted)
			SPAC15A10.06	SPAC15A10.06	CPA1 sodium ion/proton antiporter (predicted)		SPAC3A11.08	pcu4	cullin 4		SPCC1919.10c	myo52	myosin type V
			SPAC3A11.08	pcu4	cullin 4		SPBC651.03c	gyp10	GTPase activating protein Gyp10		SPBC2G2.01c	liz1	plasma membrane pantothenate transmembrane transporter Liz1
			SPBC2A9.06c	nus1	di-trans,poly-cis-decaprenylcistransferase Nus1		SPAC1834.05	alg9	mannosyltransferase complex subunit Alg9 (predicted)		SPBC30D10.13c	pdb1	pyruvate dehydrogenase e1 component beta subunit Pdb1
			SPCC757.13	SPCC757.13	dipeptide transmembrane transporter (predicted)		SPCC1919.10c	myo52	myosin type V		SPAC823.03	ppk15	serine/threonine protein kinase Ppk15 (predicted)
			SPAC22F8.04	pet1	Golgi phosphoenolpyruvate transmembrane transporter Pet1						SPBC800.05c	atb2	tubulin alpha 2
			SPAC13G6.03	gpi7	GPI anchor biosynthesis protein Gpi7 (predicted)								
			SPAC1834.05	alg9	mannosyltransferase complex subunit Alg9 (predicted)								
			SPBC119.06	sco1	mitochondrial copper chaperone for cytochrome c oxidase Sco1 (predicted)								
			SPBC30D10.10c	tor1	serine/threonine protein kinase Tor1								
			SPBC839.15c	tef103	translation elongation factor EF-1 alpha Ef1a-c								
autosomal recessive disease	MONDO:0006025	12	SPCC18.09c	hnt3	aprataxin Hnt3	5	SPAC3A11.08	pcu4	cullin 4	6	SPAC3A11.08	pcu4	cullin 4
			SPBC28E12.06c	lvs1	beige protein homolog Lvs1		SPBC651.03c	gyp10	GTPase activating protein Gyp10		SPAC4H3.13	pcc1	EKC/KEOPS complex subunit Pcc1 (predicted)
			SPAC3A11.08	pcu4	cullin 4		SPAPB2B4.02	grx5	mitochondrial [2Fe-2S] cluster assembly and transfer glutaredoxin Grx5		SPAPB2B4.02	grx5	mitochondrial [2Fe-2S] cluster assembly and transfer glutaredoxin Grx5
			SPAC222.07c	hri2	eIF2 alpha kinase Hri2		SPBC1703.13c	SPBC1703.13c	mitochondrial carrier, inorganic phosphate (predicted)		SPAC1071.03c	sil1	nucleotide exchange factor for the ER lumenal Hsp70 chaperone, Sil1 (predicted)
			SPAC4H3.13	pcc1	EKC/KEOPS complex subunit Pcc1 (predicted)		SPAC23A1.19c	hrq1	RecQ type DNA helicase Hrq1 (predicted)		SPAC823.03	ppk15	serine/threonine protein kinase Ppk15 (predicted)
			SPAC22F8.04	pet1	Golgi phosphoenolpyruvate transmembrane transporter Pet1						SPCC320.05	SPCC320.05	sulfate transmembrane transporter (predicted)
			SPAC3A11.05c	kms1	meiotic spindle pole body KASH domain protein Kms1								
			SPAC823.10c	hem25	mitochondrial carrier, glycine Hem25 (predicted)								
			SPBC543.09	yta12	mitochondrial m-AAA protease Yta12 (predicted)								
			SPAPB8E5.04c	npc2	Niemann-Pick disease type C2 protein hE1 homolog Npc2 (predicted)								
			SPBC1105.10	rav1	RAVE complex subunit Rav1								
			SPAC23A1.19c	hrq1	RecQ type DNA helicase Hrq1 (predicted)								
			SPCC18B5.11c	cds1	DNA replication checkpoint kinase Cds1		SPAC2C4.07c	dis32	3'-5'-exoribonuclease activity Dis3L2		SPAC2C4.07c	dis32	3'-5'-exoribonuclease activity Dis3L2
			SPBC24C6.08c	bhd1	Lst4-Lst7 complex subunit, folliculin Bhd1/Lst7		SPBC1778.06c	fim1	fimbrin		SPBC8D2.04	hht2	histone H3 h3.2
			SPAC458.05	pik3	phosphatidylinositol 3-kinase Pik3		SPBC3D6.04c	mad1	mitotic spindle checkpoint protein Mad1		SPBC3D6.04c	mad1	mitotic spindle checkpoint protein Mad1
			SPAC23A1.19c	hrq1	RecQ type DNA helicase Hrq1 (predicted)		SPAC144.04c	spe1	ornithine decarboxylase Spe1 (predicted)		SPAC144.04c	spe1	ornithine decarboxylase Spe1 (predicted)
			SPBC4B4.03	rsc1	RSC complex subunit Rsc1		SPAC458.05	pik3	phosphatidylinositol 3-kinase Pik3		SPAC458.05	pik3	phosphatidylinositol 3-kinase Pik3

cancer	MONDO:0004992	11	SPBC30D10.10c	tor1	serine/threonine protein kinase Tor1	7	SPAC23A1.19c	hrq1	RecQ type DNA helicase Hrq1 (predicted)	7	SPCC736.09c	tfx1	TRAX
			SPAC1D4.01	tls1	splicing factor Tls1		SPBC21C3.11	ubx4	UBX domain protein Ubx4 (predicted)		SPBC21C3.11	ubx4	UBX domain protein Ubx4 (predicted)
			SPBC646.09c	int6	translation initiation factor eIF3e								
			SPAC30.03c	tsn1	translin								
			SPCC736.09c	tfx1	TRAX								
			SPAC4F10.15c	wsp1	WASp homolog								
skin disease	MONDO:0005093	11	SPCC18.09c	hnt3	aprataxin Hnt3	5	SPBC11C11.02	imp2	F-BAR domain protein Imp2	3	SPBC11C11.02	imp2	F-BAR domain protein Imp2
			SPBC28E12.06c	lvs1	beige protein homolog Lvs1		SPBC12D12.05c	SPBC12D12.05c	mitochondrial carrier, ATP:ADP antiporter (predicted)		SPBC12D12.05c	SPBC12D12.05c	mitochondrial carrier, ATP:ADP antiporter (predicted)
			SPBC2D10.16	mhf1	CENP-S ortholog, FANCM-MHF complex subunit Mhf1		SPCC1919.10c	myo52	myosin type V		SPCC1919.10c	myo52	myosin type V
			SPBC11C11.02	imp2	F-BAR domain protein Imp2		SPAC23A1.19c	hrq1	RecQ type DNA helicase Hrq1 (predicted)				
			SPAC14C4.05c	man1	LEM domain nuclear inner membrane protein Man1, Sad1 interacting factor		SPAC4F10.15c	wsp1	WASp homolog				
			SPBC24C6.08c	bhd1	Lst4-Lst7 complex subunit, folliculin Bhd1/Lst7								
			SPBC839.06	cta3	P-type ATPase, potassium exporting Cta3								
			SPAC23A1.19c	hrq1	RecQ type DNA helicase Hrq1 (predicted)								
			SPBC776.02c	dis2	serine/threonine protein phosphatase PP1, Dis2								
			SPAC688.06c	slx4	structure-specific endonuclease subunit Slx4								
			SPAC4F10.15c	wsp1	WASp homolog								
autosomal dominant disease	MONDO:0000426	10	SPBC21B10.03c	ath1	ataxin-2 homolog	1	SPBC1778.06c	fim1	fimbrin	3	SPBC2G2.01c	liz1	plasma membrane pantothenate transmembrane transporter Liz1
			SPBC1683.12	SPBC1683.12	carboxylic acid transmembrane transporter (predicted)						SPAC823.03	ppk15	serine/threonine protein kinase Ppk15 (predicted)
			SPBC2A9.06c	nus1	di-trans,poly-cis-decaprenylcistransferase Nus1						SPCP31B10.07	eft202	translation elongation factor 2 (EF-2) Eft2,B
			SPCC18B5.11c	cds1	DNA replication checkpoint kinase Cds1								
			SPBC24C6.08c	bhd1	Lst4-Lst7 complex subunit, folliculin Bhd1/Lst7								
			SPBC530.10c	anc1	mitochondrial carrier, ATP:ADP antiporter Anc1								
			SPBC543.09	yta12	mitochondrial m-AAA protease Yta12 (predicted)								
			SPAC644.06c	cdr1	NIM1 family serine/threonine protein kinase Cdr1/Nim1								
			SPBC1105.10	rav1	RAVE complex subunit Rav1								
			SPCC126.04c	sgf73	SAGA complex deubiquitinating submodule subunit Sgf73								
hematologic disease	MONDO:0005570	10	SPBC18H10.13	rps1402	40S ribosomal protein S14 (predicted)	6	SPBC18H10.13	rps1402	40S ribosomal protein S14 (predicted)	2	SPCC285.15c	rps2802	40S ribosomal protein S28, Rps2802
			SPBC17G9.10	rpl1102	60S ribosomal protein L11 (predicted)		SPBC839.05c	rps1701	40S ribosomal protein S17 (predicted)		SPAPB2B4.02	grx5	mitochondrial [2Fe-2S] cluster assembly and transfer glutaredoxin Grx5
			SPBC28E12.06c	lvs1	beige protein homolog Lvs1		SPCC285.15c	rps2802	40S ribosomal protein S28, Rps2802				
			SPBC2D10.16	mhf1	CENP-S ortholog, FANCM-MHF complex subunit Mhf1		SPAPB2B4.02	grx5	mitochondrial [2Fe-2S] cluster assembly and transfer glutaredoxin Grx5				
			SPBC4F6.05c	emp46	lectin family glycoprotein receptor Emp46 (predicted)		SPBC725.15	ura5	orotate phosphoribosyltransferase Ura5				
			SPAC823.10c	hem25	mitochondrial carrier, glycine Hem25 (predicted)		SPAC4F10.15c	wsp1	WASp homolog				
			SPBC725.15	ura5	orotate phosphoribosyltransferase Ura5								
			SPAC688.06c	slx4	structure-specific endonuclease subunit Slx4								
			SPCC1183.06	ung1	uracil DNA N-glycosylase Ung1								
			SPAC4F10.15c	wsp1	WASp homolog								
carbohydrate metabolism disease	MONDO:0037792	3	SPAC1556.08c	cbs2	AMP-activated protein kinase gamma subunit Cbs2	0				2	SPACUNK4.10	gor1	glyoxylate reductase (predicted)
			SPBPB2B2.13	gal1	galactokinase Gal1						SPBC30D10.13c	pdb1	pyruvate dehydrogenase e1 component beta subunit Pdb1
			SPAC22F8.04	pet1	Golgi phosphoenolpyruvate transmembrane transporter Pet1								
immune system disease	MONDO:0005046	8	SPBC28E12.06c	lvs1	beige protein homolog Lvs1	4	SPBC685.06	rps001	40S ribosomal protein S0A (p40)	2	SPBC11C11.02	imp2	F-BAR domain protein Imp2
			SPBC11C11.02	imp2	F-BAR domain protein Imp2		SPBC11C11.02	imp2	F-BAR domain protein Imp2		SPBC12D12.05c	SPBC12D12.05c	mitochondrial carrier, ATP:ADP antiporter (predicted)
			SPAC22F8.04	pet1	Golgi phosphoenolpyruvate transmembrane transporter Pet1		SPBC12D12.05c	SPBC12D12.05c	mitochondrial carrier, ATP:ADP antiporter (predicted)				
			SPAPB8E5.04c	npc2	Niemann-Pick disease type C2 protein hE1 homolog Npc2 (predicted)		SPAC4F10.15c	wsp1	WASp homolog				
			SPCC1919.05	ski3	Ski complex TPR repeat subunit Ski3 (predicted)								
			SPCC364.02c	bis1	splicing factor Bis1								
			SPCC1183.06	ung1	uracil DNA N-glycosylase Ung1								
			SPAC4F10.15c	wsp1	WASp homolog								
peripheral neuropathy	MONDO:0005244	6	SPBC3H7.03c	kgd1	oglutarate dehydrogenase (lipoamide) (e1 component of oxoglutarate dehydrogenase complex) (predi	0				2	SPAC1071.03c	sil1	nucleotide exchange factor for the ER lumenal Hsp70 chaperone, Sil1 (predicted)
			SPBC21B10.03c	ath1	ataxin-2 homolog						SPBC30D10.13c	pdb1	pyruvate dehydrogenase e1 component beta subunit Pdb1
			SPBC28E12.06c	lvs1	beige protein homolog Lvs1								
			SPBC17A3.09c	aim22	lipoate-protein ligase A (predicted)								
			SPBC119.06	sco1	mitochondrial copper chaperone for cytochrome c oxidase Sco1 (predicted)								
			SPBC1105.10	rav1	RAVE complex subunit Rav1								
inherited lipid metabolism disorder	MONDO:0002525	7	SPBC27B12.03c	erg32	C-5 sterol desaturase Erg32	3	SPBC1734.12c	alg12	lichyl pyrophosphate Man7GlcNAc2 alpha-1,6-mannosyltransferase Alg12 (predict	3	SPBC1734.12c	alg12	lichyl pyrophosphate Man7GlcNAc2 alpha-1,6-mannosyltransferase Alg12 (predicte
			SPBC1734.12c	alg12	dolichyl pyrophosphate Man7GlcNAc2 alpha-1,6-mannosyltransferase Alg12 (predicted)		SPAC1834.05	alg9	mannosyltransferase complex subunit Alg9 (predicted)		SPAC1834.05	alg9	mannosyltransferase complex subunit Alg9 (predicted)
			SPAC13G6.03	gpi7	GPI anchor biosynthesis protein Gpi7 (predicted)		SPAPB2B4.02	grx5	mitochondrial [2Fe-2S] cluster assembly and transfer glutaredoxin Grx5		SPAPB2B4.02	grx5	mitochondrial [2Fe-2S] cluster assembly and transfer glutaredoxin Grx5
			SPBC17A3.09c	aim22	lipoate-protein ligase A (predicted)								
			SPAC1834.05	alg9	mannosyltransferase complex subunit Alg9 (predicted)								
			SPBC530.10c	anc1	mitochondrial carrier, ATP:ADP antiporter Anc1								
			SPAPB8E5.04c	npc2	Niemann-Pick disease type C2 protein hE1 homolog Npc2 (predicted)								
anemia (disease)	MONDO:0002280	6	SPBC18H10.13	rps1402	40S ribosomal protein S14 (predicted)	5	SPBC18H10.13	rps1402	40S ribosomal protein S14 (predicted)	2	SPCC285.15c	rps2802	40S ribosomal protein S28, Rps2802
			SPBC17G9.10	rpl1102	60S ribosomal protein L11 (predicted)		SPBC839.05c	rps1701	40S ribosomal protein S17 (predicted)		SPAPB2B4.02	grx5	mitochondrial [2Fe-2S] cluster assembly and transfer glutaredoxin Grx5
			SPBC2D10.16	mhf1	CENP-S ortholog, FANCM-MHF complex subunit Mhf1		SPCC285.15c	rps2802	40S ribosomal protein S28, Rps2802				
			SPAC823.10c	hem25	mitochondrial carrier, glycine Hem25 (predicted)		SPAPB2B4.02	grx5	mitochondrial [2Fe-2S] cluster assembly and transfer glutaredoxin Grx5				
			SPBC725.15	ura5	orotate phosphoribosyltransferase Ura5		SPBC725.15	ura5	orotate phosphoribosyltransferase Ura5				
			SPAC688.06c	slx4	structure-specific endonuclease subunit Slx4								
bone development disease	MONDO:0005497	6	SPBC2D10.16	mhf1	CENP-S ortholog, FANCM-MHF complex subunit Mhf1	3	SPBC1778.06c	fim1	fimbrin	1	SPCC1919.10c	myo52	myosin type V
			SPAC222.07c	hri2	eIF2 alpha kinase Hri2		SPCC1919.10c	myo52	myosin type V				
			SPAC14C4.05c	man1	LEM domain nuclear inner membrane protein Man1, Sad1 interacting factor		SPAC23A1.19c	hrq1	RecQ type DNA helicase Hrq1 (predicted)				
			SPAC23A1.19c	hrq1	RecQ type DNA helicase Hrq1 (predicted)								
			SPAC16C9.02c	mta1	S-methyl-5-thioadenosine phosphorylase Mta1								
			SPAC688.06c	slx4	structure-specific endonuclease subunit Slx4								
digestive system disease	MONDO:0004335	6	SPCP1E11.05c	are2	acyl-coA-sterol acyltransferase Are2	3	SPCP1E11.05c	are2	acyl-coA-sterol acyltransferase Are2	3	SPCP1E11.05c	are2	acyl-coA-sterol acyltransferase Are2
			SPAC222.07c	hri2	eIF2 alpha kinase Hri2		SPCC1919.10c	myo52	myosin type V		SPCC1919.10c	myo52	myosin type V
			SPBC24C6.08c	bhd1	Lst4-Lst7 complex subunit, folliculin Bhd1/Lst7		SPAC144.04c	spe1	ornithine decarboxylase Spe1 (predicted)		SPAC144.04c	spe1	ornithine decarboxylase Spe1 (predicted)
			SPBC1105.10	rav1	RAVE complex subunit Rav1								
			SPCC1919.05	ski3	Ski complex TPR repeat subunit Ski3 (predicted)								

			SPAC688.06c	slx4	structure-specific endonuclease subunit Slx4								
heart disease	MONDO:0005267	6	SPAC1556.08c<br>SPBC530.10c<br>SPAC1F3.10c<br>SPAC222.05c<br>SPBC776.02c<br>SPCC364.02c	cbs2<br>anc1<br>oct1<br>mss1<br>dis2<br>bis1	AMP-activated protein kinase gamma subunit Cbs2<br>mitochondrial carrier, ATP:ADP antiporter Anc1<br>mitochondrial intermediate peptidase Oct1 (predicted)<br>mitochondrial tRNA wobble uridine modification GTPase Mss1 (predicted)<br>serine/threonine protein phosphatase PP1, Dis2<br>splicing factor Bis1	1	SPBC1703.13c	SPBC1703.13c	mitochondrial carrier, inorganic phosphate (predicted)	0			
neurodegenerative disease	MONDO:0005559	5	SPCC18.09c<br>SPBC21B10.03c<br>SPBC28E12.06c<br>SPBC543.09<br>SPCC126.04c	hnt3<br>ath1<br>lvs1<br>yta12<br>sgf73	aprataxin Hnt3<br>ataxin-2 homolog<br>beige protein homolog Lvs1<br>mitochondrial m-AAA protease Yta12 (predicted)<br>SAGA complex deubiquitinating submodule subunit Sgf73	0				3	SPAC19G12.08<br>SPAC1071.03c<br>SPCP31B10.07	scs7<br>sil1<br>eft202	ER sphingosine hydroxylase Scs7<br>nucleotide exchange factor for the ER lumenal Hsp70 chaperone, Sil1 (predicted)<br>translation elongation factor 2 (EF-2) Eft2,B
cognitive disorder	MONDO:0002039	4	SPCC18.09c<br>SPBC21B10.03c<br>SPBC543.09<br>SPCC126.04c	hnt3<br>ath1<br>yta12<br>sgf73	aprataxin Hnt3<br>ataxin-2 homolog<br>mitochondrial m-AAA protease Yta12 (predicted)<br>SAGA complex deubiquitinating submodule subunit Sgf73	0				2	SPAC1071.03c<br>SPCP31B10.07	sil1<br>eft202	nucleotide exchange factor for the ER lumenal Hsp70 chaperone, Sil1 (predicted)<br>translation elongation factor 2 (EF-2) Eft2,B
lysosomal storage disease	MONDO:0002561	4	SPBC28E12.06c<br>SPBC1683.12<br>SPCC757.13<br>SPAPB8E5.04c	lvs1<br>SPBC1683.12<br>SPCC757.13<br>npc2	beige protein homolog Lvs1<br>carboxylic acid transmembrane transporter (predicted)<br>dipeptide transmembrane transporter (predicted)<br>Niemann-Pick disease type C2 protein hE1 homolog Npc2 (predicted)	0				1	SPBC2G2.01c	liz1	plasma membrane pantothenate transmembrane transporter Liz1
myopathy	MONDO:0005336	4	SPBC530.10c<br>SPBC119.06<br>SPAC30C2.06c<br>SPBC839.06	anc1<br>sco1<br>dml1<br>cta3	mitochondrial carrier, ATP:ADP antiporter Anc1<br>mitochondrial copper chaperone for cytochrome c oxidase Sco1 (predicted)<br>mitochondrial inheritance GTPase, tubulin-like (predicted)<br>P-type ATPase, potassium exporting Cta3	0				0			
premature aging syndrome	MONDO:0019303	4	SPBC17G9.10<br>SPBC2D10.16<br>SPAC23A1.19c<br>SPAC688.06c	rpl1102<br>mhf1<br>hrq1<br>slx4	60S ribosomal protein L11 (predicted)<br>CENP-S ortholog, FANCM-MHF complex subunit Mhf1<br>RecQ type DNA helicase Hrq1 (predicted)<br>structure-specific endonuclease subunit Slx4	4	SPBC839.05c<br>SPCC285.15c<br>SPBC12D12.05c<br>SPAC23A1.19c	rps1701<br>rps2802<br>SPBC12D12.05c<br>hrq1	40S ribosomal protein S17 (predicted)<br>40S ribosomal protein S28, Rps2802<br>mitochondrial carrier, ATP:ADP antiporter (predicted)<br>RecQ type DNA helicase Hrq1 (predicted)	2	SPCC285.15c<br>SPBC12D12.05c	rps2802<br>SPBC12D12.05c	40S ribosomal protein S28, Rps2802<br>mitochondrial carrier, ATP:ADP antiporter (predicted)
DNA repair disease	MONDO:0021190	3	SPCC18.09c<br>SPBC2D10.16<br>SPAC688.06c	hnt3<br>mhf1<br>slx4	aprataxin Hnt3<br>CENP-S ortholog, FANCM-MHF complex subunit Mhf1<br>structure-specific endonuclease subunit Slx4	0				0			
nborn disorder of purine or pyrimidine metabolis	MONDO:0019254	2	SPAC23A1.03<br>SPBC725.15	apt1<br>ura5	adenine phosphoribosyltransferase (APRT) Apt1<br>orotate phosphoribosyltransferase Ura5	4	SPAC14C4.14<br>SPAPB2B4.02<br>SPBC1703.13c<br>SPAC25B8.06c	atp1<br>grx5<br>SPBC1703.13c<br>dia4	F1-FO ATP synthase alpha subunit<br>mitochondrial [2Fe-2S] cluster assembly and transfer glutaredoxin Grx5<br>mitochondrial carrier, inorganic phosphate (predicted)<br>mitochondrial serine-tRNA ligase (predicted	1	SPAC23A1.03	apt1	adenine phosphoribosyltransferase (APRT) Apt1
inherited amino acid metabolic disorder	MONDO:0004736	3	SPAC23A1.03<br>SPBC8D2.18c<br>SPBC28E12.06c	apt1<br>SPBC8D2.18c<br>lvs1	adenine phosphoribosyltransferase (APRT) Apt1<br>adenosylhomocysteinase (predicted)<br>beige protein homolog Lvs1	2	SPAC23A1.03<br>SPCC1919.10c	apt1<br>myo52	adenine phosphoribosyltransferase (APRT) Apt1<br>myosin type V	2	SPAC23A1.03<br>SPCC1919.10c	apt1<br>myo52	adenine phosphoribosyltransferase (APRT) Apt1<br>myosin type V
nonsyndromic genetic deafness	MONDO:0019497	3	SPBC1683.12<br>SPAC3A11.05c<br>SPBC1105.10	SPBC1683.12<br>kms1<br>rav1	carboxylic acid transmembrane transporter (predicted)<br>meiotic spindle pole body KASH domain protein Kms1<br>RAVE complex subunit Rav1	0				2	SPBC2G2.01c<br>SPCC320.05	liz1<br>SPCC320.05	plasma membrane pantothenate transmembrane transporter Liz1<br>sulfate transmembrane transporter (predicted)
diabetes mellitus (disease)	MONDO:0005015	2	SPAC222.07c<br>SPBC1105.10	hri2<br>rav1	eIF2 alpha kinase Hri2<br>RAVE complex subunit Rav1	0				0			
hepatobiliary disease	MONDO:0002515	1	SPCC1919.05	ski3	Ski complex TPR repeat subunit Ski3 (predicted)	1	SPCC1919.10c	myo52	myosin type V	1	SPCC1919.10c	myo52	myosin type V
dystonic disorder	MONDO:0003441	0				1	SPCC1919.10c	myo52	myosin type V	2	SPCC1919.10c<br>SPBC800.05c	myo52<br>atb2	myosin type V<br>tubulin alpha 2
peroxisomal disease	MONDO:0019053	0				1	SPCC757.07c	ctt1	catalase	0			
