# Supplementary material for: Global network analysis in Schizosaccharomyces pombe reveals three distinct consequences of the common 1-kb deletion causing juvenile CLN3 disease
Source: Sci Rep. 2021 Mar 18;11:6332. doi: 10.1038/s41598-021-85471-4 (PMC7973434; doi:10.1038/s41598-021-85471-4)
Supplement: Supplementary file 12 — S12: Supplementary Table 12. [file 41598_2021_85471_MOESM12_ESM.pdf]

# Global network analysis in *Schizosaccharomyces pombe* reveals three distinct consequences of the common 1-kb deletion causing juvenile CLN3 disease

Christopher J. Minnis[1,2], StJohn Townsend[3,4], Julia Petschnigg[1], Elisa Tinelli[1], Jürg Bähler[3], Claire Russell[2], Sara E. Mole[1]

*[1]MRC Laboratory for Molecular Cell Biology and Great Ormond Street Institute of Child Health, University College London, London WC1E 6BT, UK*
*[2]Dept. Comparative Biomedical Sciences, Royal Veterinary College, Royal College Street, London NW1 0TU, UK*
*[3]Institute of Healthy Ageing, Department of Genetics, Evolution and Environment, University College London, London WC1E 6BT, UK*
*[4]The Molecular Biology of Metabolism Laboratory, The Francis Crick Institute, London, NW1 1AT, United Kingdom*

*Corresponding author: christopher.minnis.15@ucl.ac.uk

Supplementary table 1: Summary of MONDO disease terms and their corresponding genes for btn1(102-208del) unique interactions

| Disease terms | MONDO ID | # genes | Systematic ID | Gene name | Product description |
|---|---|---|---|---|---|
| anemia (disease) | MONDO:0002280 | 1 | SPAC823.10c | hem25 | mitochondrial carrier, glycine Hem25 (predicted) |
| autosomal dominant disease | MONDO:0000426 | 4 | SPBC21B10.03c | ath1 | ataxin-2 homolog |
| | | | SPBC1683.12 | SPBC1683.12 | carboxylic acid transmembrane transporter (predicted) |
| | | | SPCC18B5.11c | cds1 | DNA replication checkpoint kinase Cds1 |
| | | | SPBC1105.10 | rav1 | RAVE complex subunit Rav1 |
| autosomal recessive disease | MONDO:0006025 | 7 | SPCC18.09c | hnt3 | aprataxin Hnt3 |
| | | | SPAC222.07c | hri2 | eIF2 alpha kinase Hri2 |
| | | | SPAC3A11.05c | kms1 | meiotic spindle pole body KASH domain protein Kms1 |
| | | | SPAC823.10c | hem25 | mitochondrial carrier, glycine Hem25 (predicted) |
| | | | SPAPB8E5.04c | npc2 | Niemann-Pick disease type C2 protein hE1 homolog Npc2 (predicted) |
| | | | SPBC1105.10 | rav1 | RAVE complex subunit Rav1 |
| | | | SPBC713.07c | SPBC713.07c | vacuolar polyphosphatase (predicted) |
| bone development disease | MONDO:0005497 | 1 | SPAC222.07c | hri2 | eIF2 alpha kinase Hri2 |
| cancer | MONDO:0004992 | 2 | SPCC18B5.11c | cds1 | DNA replication checkpoint kinase Cds1 |
| | | | SPBC4B4.03 | rsc1 | RSC complex subunit Rsc1 |
| carbohydrate metabolism disease | MONDO:0037792 | 1 | SPBPB2B2.13 | gal1 | galactokinase Gal1 |
| cognitive disorder | MONDO:0002039 | 3 | SPCC18.09c | hnt3 | aprataxin Hnt3 |
| | | | SPBC21B10.03c | ath1 | ataxin-2 homolog |
| | | | SPCC1827.07c | SPCC1827.07c | SPX/EXS domain protein (predicted) |
| diabetes mellitus (disease) | MONDO:0005015 | 2 | SPAC222.07c | hri2 | eIF2 alpha kinase Hri2 |
| | | | SPBC1105.10 | rav1 | RAVE complex subunit Rav1 |
| digestive system disease | MONDO:0004335 | 3 | SPAC222.07c | hri2 | eIF2 alpha kinase Hri2 |
| | | | SPBC1105.10 | rav1 | RAVE complex subunit Rav1 |
| | | | SPCC1919.05 | ski3 | Ski complex TPR repeat subunit Ski3 (predicted) |
| DNA repair disease | MONDO:0021190 | 1 | SPCC18.09c | hnt3 | aprataxin Hnt3 |
| epilepsy | MONDO:0005027 | 4 | SPAC8E11.02c | rad24 | 14-3-3 protein Rad24 |
| | | | SPBC1683.12 | SPBC1683.12 | carboxylic acid transmembrane transporter (predicted) |
| | | | SPBC839.15c | tef103 | translation elongation factor EF-1 alpha Ef1a-c |
| | | | SPBC713.07c | SPBC713.07c | vacuolar polyphosphatase (predicted) |
| eye disease | MONDO:0005328 | 7 | SPBC3E7.01 | fab1 | 1-phosphatidylinositol-3-phosphate 5-kinase Fab1 |
| | | | SPCC18.09c | hnt3 | aprataxin Hnt3 |
| | | | SPBC21B10.03c | ath1 | ataxin-2 homolog |
| | | | SPBPB2B2.13 | gal1 | galactokinase Gal1 |
| | | | SPBC211.06 | gfh1 | gamma tubulin complex GPC4 subunit Gfh1 |
| | | | SPBC17A3.09c | aim22 | lipoate-protein ligase A (predicted) |
| | | | SPBC713.07c | SPBC713.07c | vacuolar polyphosphatase (predicted) |
| heart disease | MONDO:0005267 | 1 | SPAC222.05c | mss1 | mitochondrial tRNA wobble uridine modification GTPase Mss1 (predicted) |
| hematologic disease | MONDO:0005570 | 1 | SPAC823.10c | hem25 | mitochondrial carrier, glycine Hem25 (predicted) |
| hepatobiliary disease | MONDO:0002515 | 1 | SPCC1919.05 | ski3 | Ski complex TPR repeat subunit Ski3 (predicted) |
| immune system disease | MONDO:0005046 | 3 | SPAPB8E5.04c | npc2 | Niemann-Pick disease type C2 protein hE1 homolog Npc2 (predicted) |
| | | | SPCC1919.05 | ski3 | Ski complex TPR repeat subunit Ski3 (predicted) |
| | | | SPBC713.07c | SPBC713.07c | vacuolar polyphosphatase (predicted) |
| inborn disorder of purine or pyrimidine metabolism | MONDO:0019254 | 4 | SPBC29A3.18 | cyt1 | cytochrome c1 Cyt1 (predicted) |
| | | | SPBC17A3.09c | aim22 | lipoate-protein ligase A (predicted) |

| | | | | | |
|---|---|---|---|---|---|
| | | | SPAC17G6.15c | fsf1 | mitochondrial carrier, serine Fsf1 (predicted) |
| | | | SPAC222.05c | mss1 | mitochondrial tRNA wobble uridine modification GTPase Mss1 (predicted) |
| inborn mitochondrial metabolism disorder | MONDO:0004069 | 5 | SPBC29A3.18 | cyt1 | cytochrome c1 Cyt1 (predicted) |
| | | | SPBC17A3.09c | aim22 | lipoate-protein ligase A (predicted) |
| | | | SPAC823.10c | hem25 | mitochondrial carrier, glycine Hem25 (predicted) |
| | | | SPAC17G6.15c | fsf1 | mitochondrial carrier, serine Fsf1 (predicted) |
| | | | SPAC222.05c | mss1 | mitochondrial tRNA wobble uridine modification GTPase Mss1 (predicted) |
| inherited amino acid metabolic disorder | MONDO:0004736 | 1 | SPBC8D2.18c | SPBC8D2.18c | adenosylhomocysteinase (predicted) |
| inherited lipid metabolism disorder | MONDO:0002525 | 4 | SPBC27B12.03c | erg32 | C-5 sterol desaturase Erg32 |
| | | | SPBC17A3.09c | aim22 | lipoate-protein ligase A (predicted) |
| | | | SPAPB8E5.04c | npc2 | Niemann-Pick disease type C2 protein hE1 homolog Npc2 (predicted) |
| | | | SPBC713.07c | SPBC713.07c | vacuolar polyphosphatase (predicted) |
| kidney disease | MONDO:0005240 | 3 | SPCC18B5.11c | cds1 | DNA replication checkpoint kinase Cds1 |
| | | | SPBPB2B2.13 | gal1 | galactokinase Gal1 |
| | | | SPBC4B4.03 | rsc1 | RSC complex subunit Rsc1 |
| lysosomal storage disease | MONDO:0002561 | 3 | SPBC1683.12 | SPBC1683.12 | carboxylic acid transmembrane transporter (predicted) |
| | | | SPAPB8E5.04c | npc2 | Niemann-Pick disease type C2 protein hE1 homolog Npc2 (predicted) |
| | | | SPBC713.07c | SPBC713.07c | vacuolar polyphosphatase (predicted) |
| neurodegenerative disease | MONDO:0005559 | 2 | SPCC18.09c | hnt3 | aprataxin Hnt3 |
| | | | SPBC21B10.03c | ath1 | ataxin-2 homolog |
| nonsyndromic genetic deafness | MONDO:0019497 | 3 | SPBC1683.12 | SPBC1683.12 | carboxylic acid transmembrane transporter (predicted) |
| | | | SPAC3A11.05c | kms1 | meiotic spindle pole body KASH domain protein Kms1 |
| | | | SPBC1105.10 | rav1 | RAVE complex subunit Rav1 |
| peripheral neuropathy | MONDO:0005244 | 4 | SPBC21B10.03c | ath1 | ataxin-2 homolog |
| | | | SPBC17A3.09c | aim22 | lipoate-protein ligase A (predicted) |
| | | | SPBC1105.10 | rav1 | RAVE complex subunit Rav1 |
| | | | SPBC713.07c | SPBC713.07c | vacuolar polyphosphatase (predicted) |
| skin disease | MONDO:0005093 | 1 | SPCC18.09c | hnt3 | aprataxin Hnt3 |
| dystonic disorder | MONDO:0003441 | 0 | | | |
| inborn disorder of the gamma-glutamyl cycle | MONDO:0019241 | 0 | | | |
| inherited porphyria | MONDO:0019142 | 0 | | | |
| myopathy | MONDO:0005336 | 0 | | | |
| obsolete monogenic disease (obsolete MONDO:0000275 | MONDO:0000275 | 0 | | | |
| peroxisomal disease | MONDO:0019053 | 0 | | | |
| premature aging syndrome | MONDO:0019303 | 0 | | | |
| proteostasis deficiencies | MONDO:0021179 | 0 | | | |
