# Supplementary material for: Global network analysis in Schizosaccharomyces pombe reveals three distinct consequences of the common 1-kb deletion causing juvenile CLN3 disease
Source: Sci Rep. 2021 Mar 18;11:6332. doi: 10.1038/s41598-021-85471-4 (PMC7973434; doi:10.1038/s41598-021-85471-4)
Supplement: Supplementary file 13 — S13: Supplementary Table 13. [file 41598_2021_85471_MOESM13_ESM.pdf]

Global network analysis in *Schizosaccharomyces pombe* reveals three distinct consequences of the common 1-kb deletion causing juvenile CLN3 disease

Christopher J. Minnis[1,2], StJohn Townsend[3,4], Julia Petschnigg[1], Elisa Tinelli[1], Jürg Bähler[3], Claire Russell[2], Sara E. Mole[1]

[1]*MRC Laboratory for Molecular Cell Biology and Great Ormond Street Institute of Child Health, University College London, London WC1E 6BT, UK*
[2]*Dept. Comparative Biomedical Sciences, Royal Veterinary College, Royal College Street, London NW1 0TU, UK*
[3]*Institute of Healthy Ageing, Department of Genetics, Evolution and Environment, University College London, London WC1E 6BT, UK*
[4]*The Molecular Biology of Metabolism Laboratory, The Francis Crick Institute, London, NW1 1AT, United Kingdom*

*Corresponding author: christopher.minnis.15@ucl.ac.uk

Supplementary table 1 : Summary of overlapping orthologues for genetic interactions from previously published datasets

Strains	Elshatory 2003				Lurio 2006				Scifo 2013				Lebrum 2011			
	ID	Yeast name	Mouse orthologue	DIOPT conversation rank	ID	Yeast name	Mouse orthologue	DIOPT conversation rank	ID	Yeast name	Human orthologue	DIOPT conversation rank	ID	Yeast name	Human orthologue	DIOPT conversation rank
btn1Δ	None	None	None	None	None	None	None	None	SPBC337.08c	ubi4	UBC	High	SPAC29A4.18	prw1	RBBP4	Moderate
									SPCC1739.14	npp106	NUP93	moderate	SPAC16E8.01	shd1	EP300	Low
									SPCC1919.10c	myo52	MYO10	low	SPAC26H5.05	mga2	CAMTA1	Low
													SPCC1620.04c	fzr3	CDC20	moderate
													SPBC26H8.14c	cox17	COX17	High
													SPCC1919.10c	myo52	MYO6	Low
													SPAC1486.04c	alm1	MTUS1	Low
													SPBC2F12.15c	pfa3	ZDHHC21	moderate
													SPBC336.03	efc25	RASGEF1A	Low
													SPBC1271.12	kes1	OSBPL5	Moderate
btn1(D363G)	None	None	None	None	SPAC26F1.10c	pyp1	Dusp7	Low	SPBC1703.13c	SPBC1703.13c	SLC25A3	high	SPAC29A4.18	prw1	RBBP4	Moderate
									SPCC1739.14	npp106	NUP93	moderate	SPAC26H5.05	mga2	CAMTA1	Low
									SPCC1919.10c	myo52	MYO10	low	SPCC1620.04c	fzr3	CDC20	moderate
													SPCC1919.10c	myo52	MYO6	Low
													SPAC1486.04c	alm1	MTUS1	Low
													SPBC2F12.15c	pfa3	ZDHHC21	Moderate
													SPBC336.03	efc25	RASGEF1A	Low
													SPBC1347.06c	cki1	CSNK1A1	Low
													SPBC1271.12	kes1	OSBPL5	Moderate
													SPAC26F1.10c	pyp1	PTPRM	Low
btn1(102-208del)	None	None	None	None	SPAC26F1.10c	pyp1	Dusp7	Low	SPAC17G6.15c	SPAC17G6.15c	SFXN1	moderate	SPBC8D2.18c	SPBC8D2.18c	AHCYL1	Low
					SPAC26F1.10c	SPAC26F1.10c	Txnip	moderate			SFXN3	moderate	SPAC1556.08c	cbs2	PRKAG2	Moderate
											SFXN4	moderate	SPBC365.11	grp2	KRT72	Low
									SPAC18B11.04	ncs1	KCNIP3	Low	SPBC1604.08c	imp1	KPNA3	Moderate
									SPBC29A3.18	cyt1	CYC1	High	SPBC543.07	pek1	MAP2K5	Low
									SPBC2G5.01	SPBC2G5.01	CCDC47	high	SPBC409.07c	wis1	MAP2K5	Low
									SPBC530.10c	anc1	SLC25A4	High	SPBC16E9.17c	rem1	CCNB1	Moderate
											SLC25A5	High	SPBC26H8.14c	cox17	COX17	High
											SLC25A6	Moderate	SPAC2F7.17	mrf1	MTRF1	Moderate
									SPBC543.09	yta12	AFG3L2	High	SPAC222.05c	mss1	ERAL1	Low
									SPBC839.11c	hut1	SLC35B2	moderate	SPBC2F12.15c	pfa3	ZDHHC21	Moderate
													SPAC1A6.04c	plb1	PLA2G4B	Low
													SPBC336.03	efc25	RASGEF1A	Low
													SPBC3H7.15	hhp1	CSNK1A1	Moderate
													SPAC23C4.12	hhp2	CSNK1A1	High
													SPBC1271.12	kes1	OSBPL5	Moderate
													SPAC26F1.10c	pyp1	PTPRM	Low
unique to *btn1(102-208del)*	None	None	None	None	SPAC26F1.10c	SPAC26F1.10c	Ywhaz	moderate	SPAC17G6.15c	SPAC17G6.15c	SFXN1	moderate	SPAC8E11.02c	rad24	YWHAH	Moderate
											SFXN3	moderate	SPBC8D2.18c	SPBC8D2.18c	AHCYL1	Low
											SFXN4	moderate	SPBC365.11	grp2	KRT72	Low
									SPAC18B11.04	ncs1	KCNIP3	Low	SPBC543.07	pek1	MAP2K5	Low
									SPBC29A3.18	cyt1	CYC1	high	SPBC16E9.17c	rem1	CCNB1	Moderate
									SPBC839.11c	hut1	SLC35B2	moderate	SPAC2F7.17	mrf1	MTRF1	Moderate
													SPAC222.05c	mss1	ERAL1	Low
													SPAC1A6.04c	plb1	PLA2G4B	Low
													SPBC3H7.15	hhp1	CSNK1A1	Moderate
													SPAC23C4.12	hhp2	CSNK1A1	High
